# Interleaved Quadratic Boost DC-DC Converter with Extended Voltage Gain and Reduced Switch Voltage Stress for Photovoltaic Applications

**DOI:** 10.12688/openreseurope.19625.1

**Published:** 2025-02-24

**Authors:** Daniel Ferreira, Armando Cordeiro, Paulo Gambôa, Luis Rocha, Filipe Barata, José Fernando Silva, João F. Martins, Vitor Fernão Pires

**Affiliations:** 1 PolyTechnic University of Lisbon, Department of Electrical Engineering Energy and Automation, Instituto Superior de Engenharia de Lisboa (ISEL), Rua Conselheiro Emídio Navarro, Lisboa, 1, 1959-007, Portugal; 2 Department of Electrical and Computer Engineering (DEEC), Nova University of Lisbon, Faculdade de Ciência e Tecnologia (FCT), 2829-516 Caparica, Portugal, CTS-UNINOVA and LASI, Portugal, LASI, Portugal; 3 Polytechnic Institute of Setubal, Department of Electrical Engineering (DEE), Escola Superior de Tenologia de Setúbal, Campus do IPS, Estefanilha, Setúbal, 2914-508, Portugal; 4 INESC-ID Lisboa, Rua Alves Redol, Lisboa, 9, 1000-029, Portugal; 5 Department of Electrical and Computer Engineering (DEEC), University of Lisbon, Instituto Superior Técnico, Av. Rovisco Pais, Lisboa, 1, 1049-001, Portugal

**Keywords:** DC-DC Converters; Interleaved Quadratic Boost; High-Voltage Gain; High Efficiency; Photovoltaic systems

## Abstract

**Background:**

DC-DC power converters are essential devices in the modern world, playing a crucial role in managing the power supply from different power sources converting and adapting voltage levels. These power converters are fundamental to numerous applications, from charging your mobile phone to powering different types of machinery. Lately, due to climate change problems and the floating nature of most renewable power sources, they are essential to a carbon-free world and zero emissions target.

**Methods:**

Our investigation method was based on an initial theoretical approach using mathematical equations to describe the operation of the electrical circuit and evaluate the performance compared to other topologies, followed by the validation through some computational simulations using MATLAB/SIMULINK software. Next, the operation of the proposed converter was also confirmed by several experimental tests using a laboratory prototype developed exclusively for these tests.

**Results:**

Based on the achieved results, an efficiency analysis was performed showing that in addition to high-voltage gain, from the range of six to eight times the input voltage, the converter maintains a very high efficiency, around 95% to 96% up to a duty cycle of 0.50, where a voltage gain of 5.82 is achieved in a real setup Also, the optimal operating point was identified, based on the duty cycle, where the converter operates at maximum efficiency.

**Conclusions:**

In conclusion, it is possible to claim that the proposed converter presents a stable and efficient operation and has a very high potential for applications that require high-voltage gain, such as photovoltaic solar systems or even electrical vehicles or energy storage systems. Other relevant aspect is the reduced value of capacitors, due to the interleaved operation, leading to reduced stress over capacitors and distributed voltage over them.

## Plain language summary

This paper shows the study, development, and results of a new Direct-Current to Direct-Current (DC-DC) electric power converter topology, designated as interleaved quadratic Boost DC-DC converter topology. The converter topology is capable of achieving significantly higher voltage gains (higher voltage in the output when compared with the input voltage) than most conventional existing topologies. A theoretical approach was introduced in this paper and then validated through some computer simulations (using MATLAB/SIMULINK software). Finally, the performance of the topology was also confirmed in an experimental setup using a practical prototype of the proposed converter. From the experimental results, it was possible to achieve a maximum output voltage gain of over eight times the input voltage. An efficiency analysis (allowing us to identify the energy losses during the operation of the converter) was also performed, showing that the proposed topology converter maintains a very high efficiency, around 95% to 96%. The optimal operating point was also identified, based on the duty cycle (turn-on and turn-off of the power devices at a certain frequency), where the converter operates at maximum efficiency. The results show that the proposed converter has a very high potential for applications that require high-voltage gain, such as photovoltaic solar systems or even electrical vehicles or energy storage systems.

## Introduction

The increasing global demand for energy-efficient and sustainable systems has driven significant advancements in power electronics, particularly in DC-DC conversion technologies ^1^. Traditional Boost converters, while effective in many applications, often have difficulty to achieve the high-voltage gains required in modern power systems, such as photovoltaic solar systems ^2^, electrical vehicles (EV) ^3^, High-Voltage Direct Current (HVDC) power transmission systems ^4^, water pumping systems ^5^, or others. Addressing the limitations of conventional topologies, this work introduces a novel interleaved quadratic DC-DC Boost converter designed to provide significantly higher voltage gain without sacrificing efficiency.

Over the years, numerous DC-DC Boost converter topologies have been developed for different applications in a wide range of emergent multidisciplinary engineering fields, such as renewable energy sources (RES), photovoltaic solar energy conversion, EV, energy storage systems (ESS), fuel cells, among others.

Typically, DC-DC converters can be classified according to different features, such as isolated 6, 7 or non-isolated ^8, 9^, unidirectional ^10, 11^ or bidirectional ^12, 13^, voltage-fed ^14, 15^ or current-fed ^16, 17^, hard-switch ^18, 19^ or soft-switch ^20, 21^, minimum-phase ^22^ or non-minimum-phase ^23^. Most of these DC-DC converters are well represented in 24– 27. Another way to classify the DC-DC converters is specifying their voltage Boost technique. Some of the most well-known techniques are the switched capacitors ^28, 29^, voltage multiplier cells ^30, 31^, switched inductors ^32, 33^, voltage lift ^34, 35^ and multi-stage/-level ^36, 37^ topologies. A review of some of these step-up voltage techniques can be found in 38– 40. Nowadays, engineering research is also focused on the development of converters with higher reliability, higher efficiency, combined with less volume, weight and cost ^41^.

Among the DC-DC converter topologies developed recently that have stood out for the high-voltage gains obtained are those that present quadratic gains. In this way, is possible to highlight some significantly important topologies developed with such features. The solution proposed in 42 is a transformerless high step-up DC-DC converter with a quadratic voltage gain. In this converter, using a duty cycle greater than 0.309 is possible to achieve a higher voltage gain than the classic Boost converter. This solution includes three switches, five diodes, two inductors and three output capacitors. Despite its interest, this solution requires too many components when compared with other solutions. Other quadratic gain topology can be found in 43, where the authors propose a modified classic DC-DC buck-boost converter. Since this topology allows a buck-boost operation, it is only necessary to control one power switch for each operation mode and the additional power switches remain always ON or always OFF. By controlling only one power switch, they developed a setup capable of achieving a quadruple voltage gain for a duty cycle of 0.5. Another similar topology can be found in 44, which created a quadratic high-gain Boost converter, where it was possible to obtain a gain of two times the input voltage at the output with a duty cycle of 0.50. More recently a new DC-DC Boost converter setup with quadratic gain was proposed ^45^. In this solution using a duty cycle of 0.50 is also possible to achieve a triple output voltage. This solution includes one switch, three diodes, two inductors and two output capacitors. Recently, a new quadratic DC-DC Boost converter topology was proposed in 46, which can achieve a quintuple output voltage with a duty cycle of 0.50. This solution requires only one switch, four diodes, two inductors and three capacitors. The main disadvantage of this solution is that the switch must withstand the maximum output voltage.

It is well-known that high-voltage gain is critical in applications with low input voltages, such as those using a reduced number of solar panel strings or where, due to weather variability sometimes produce reduced voltages, and it required to efficiently convert them into much higher output voltages ^47^. Most quadratic DC-DC Boost converters typically offer voltage gains of three to four times the input voltage, which may not be sufficient for advanced applications. The interleaved quadratic Boost topology proposed in this study aims to overcome these limitations by achieving an extended voltage gains over eight times the input voltage, or six times considering a duty cycle of 0.50, providing a more effective solution to integrate additional RES systems. This converter is also characterized by a simple control technique, continuous input and output current, reduced switching voltage stress over the power devices. The proposed solution takes advantage of the interleaved operation, which allows to use multiple circuits (or phases) to process power in parallel. These circuits are operated with time-shifted (interleaved) switching signals to achieve improved performance compared to a single-phase or single-circuit converter. Also, the interleaved operation avoids the need of large output capacitors. The solution is also able to achieve good efficiency according to some preliminary experimental results.

This paper presents the theoretical framework behind the proposed interleaved quadratic DC-DC Boost topology, complemented by some experimental results to confirm the theoretical results and efficiency. This paper is organized into five main sections. Section I is dedicated to the introduction of this subject and importance of DC-DC converters in most modern applications, followed by a brief state-of-the-art over DC-DC converters with quadratic gain. Section II provides a detailed explanation of all the design procedures and considerations on the prototype of the proposed converter. Section III presents a comparison between the proposed converter and other interleaved quadratic Boost DC-DC converters already proposed and implemented in the literature. Section IV is dedicated to presenting and demonstrating the laboratory setup and validation of the results regarding the operation principle, voltage gain obtained and efficiency. Finally, section V presents some conclusions.

## Methods

Our investigation methodology was based on an initial theoretical approach using mathematical equations to describe the operation of the electrical circuit and evaluate the performance compared to other topologies, followed by the validation through some computational simulations using MATLAB/SIMULINK software. Next, the operation of the proposed converter was also confirmed by several experimental tests using a laboratory prototype developed exclusively for these tests. The next subsections are dedicated to show these procedures.

### Power Circuit Layout of the proposed quadratic Boost DC-DC converter

**Figure 1.**
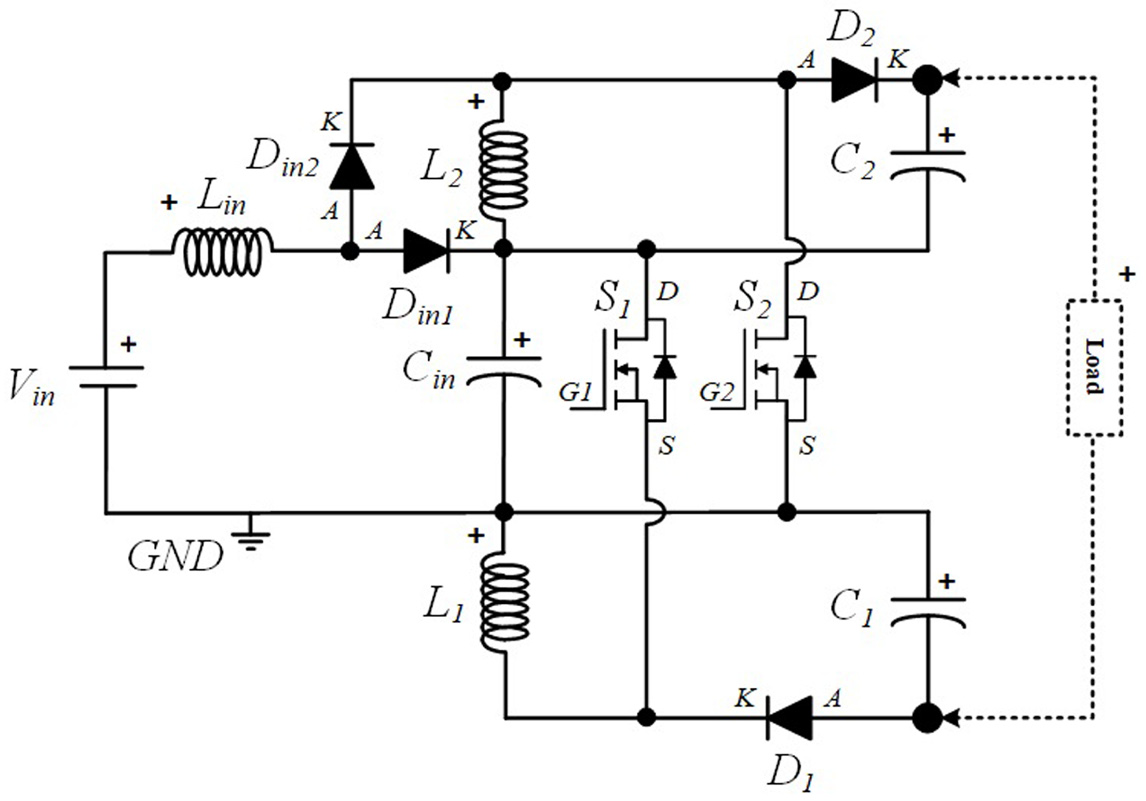
Circuit topology of the proposed interleaved quadratic DC-DC Boost Converter.

Figure 1 shows the diagram of the interleaved quadratic Boost DC-DC converter proposed in this paper. It is a new topology that has never been published before, according to extensive research conducted in the main bibliographic reference resources in the field. The power circuit consists of an input inductor, *L _i_*_*n*_, along with two input diodes, *Din* *1* and *Din* *2*, and a capacitor, *Ci* *n*. Connected to these components are two additional circuits, one at the top and another at the bottom, each consisting of and inductor, *L*_*1*_ and *L*_*2*_, a capacitor, *C*_*1*_ and *C*_*2*_, and a diode, *D*_*1*_ and *D*_*2*_, respectively. Finally, to ensure the capability of voltage regulation and Boost operation, two power MOSFET, *S*_*1*_ and *S*_*2*_, are included, controlled by a command circuit through their gates, represented in the figure as *G*_*1*_ and *G*_*2*_, respectively.

### Operation mode analysis in steady-state

**Figure 2.**
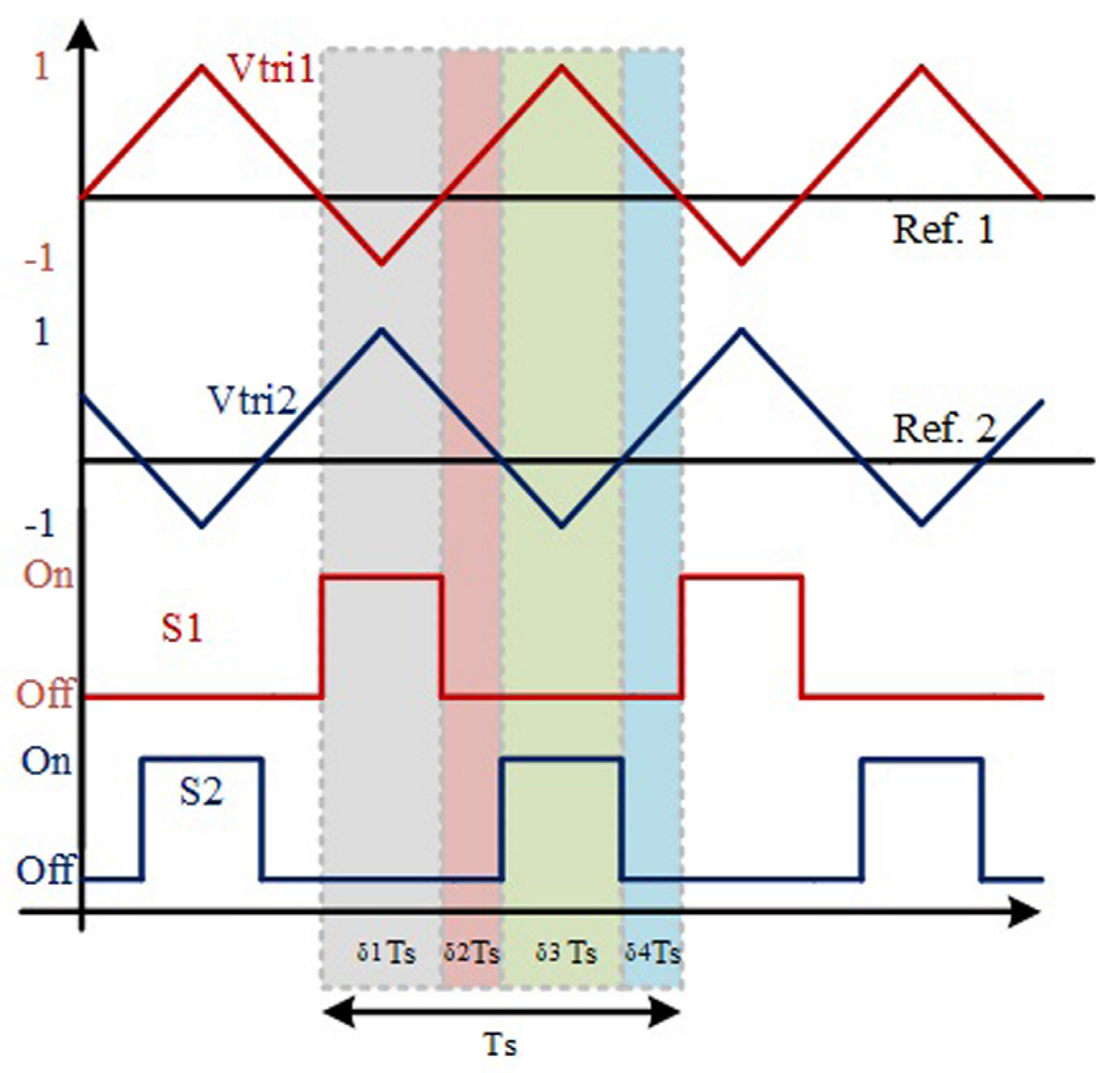
PWM switching strategy of S1 and S2.

The converter under study has four different operation modes, all in continuous conduction mode (CCM), depending on the operation of the two switches, *S*_*1*_ and *S*_*2*_. Although both power semiconductors can operate simultaneously (overlapping the conduction mode) for duty cycles above 0.50, this mode of operation is not advantageous for lower duty cycles, as it generates higher current peaks without resulting in improved voltage gain. Therefore, in the following analysis, it will be considered whether the converter operates with *S*_*1*_ turned ON and *S*_*2*_ turned OFF or *S*_*1*_ turned OFF and *S*_*2*_ turned ON or both switches turned OFF, providing four different operating intervals as explained next. Figure 2 shows a simplified representation of a classic PWM (Pulse-Width-Modulation) control strategy in order to achieve the described operation mode. This is considered an interleaved operation.

In the following figures, the four stationary operation modes are illustrated in detail, where the current flow directions in the different paths are represented with different colours to help understanding the operation principle of the converter.

**Figure 3.**
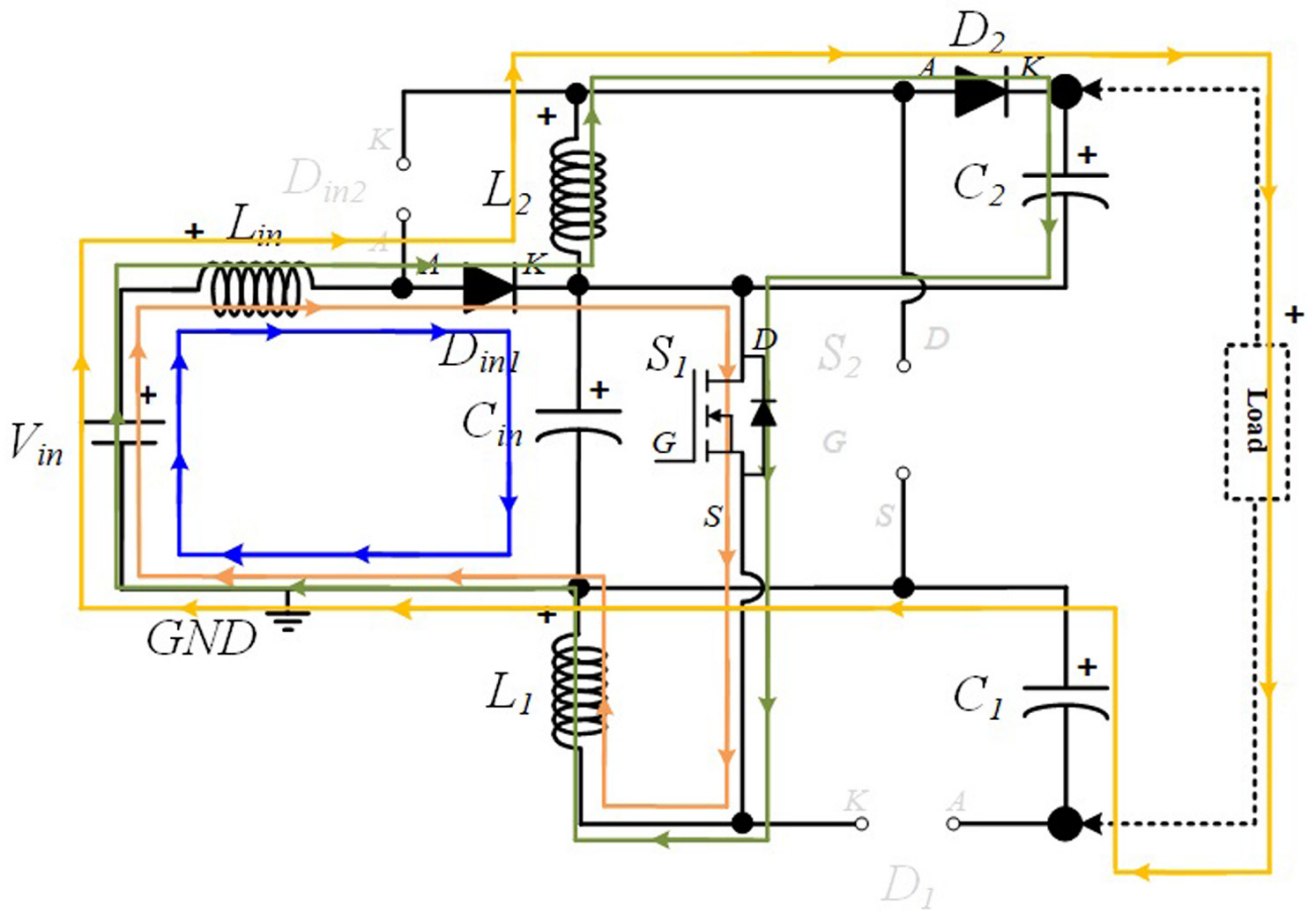
Current flow analysis when S1 turned ON and S2 turned OFF (δ1Ts).

***S1 turned ON and S2 turned OFF ( *δ1Ts*).*** During this operating mode, the input diode *D _in_*_*2*_ is turned OFF, while the input diode *D _in_*_*1*_ is turned on. Also, during this mode, the input inductor *L _i_*_*n*_, discharges the energy accumulated in the previous operating mode over the input capacitor *C _i_*_*n*_ which is in charging mode. Meanwhile, the diode *D*_*1*_ is also turned OFF because the inductor *L*_*1*_ is charging and the capacitor *C*_*1*_ is discharging, creating a reverse voltage over *D*_*1*_. On the other hand, *D*_*2*_ is turned ON, meaning that *L*_*2*_ is discharging the energy previously accumulated, and as a result, *C*_*2*_ is in charging mode. The current flow described is illustrated in Figure 3.

**Figure 4.**
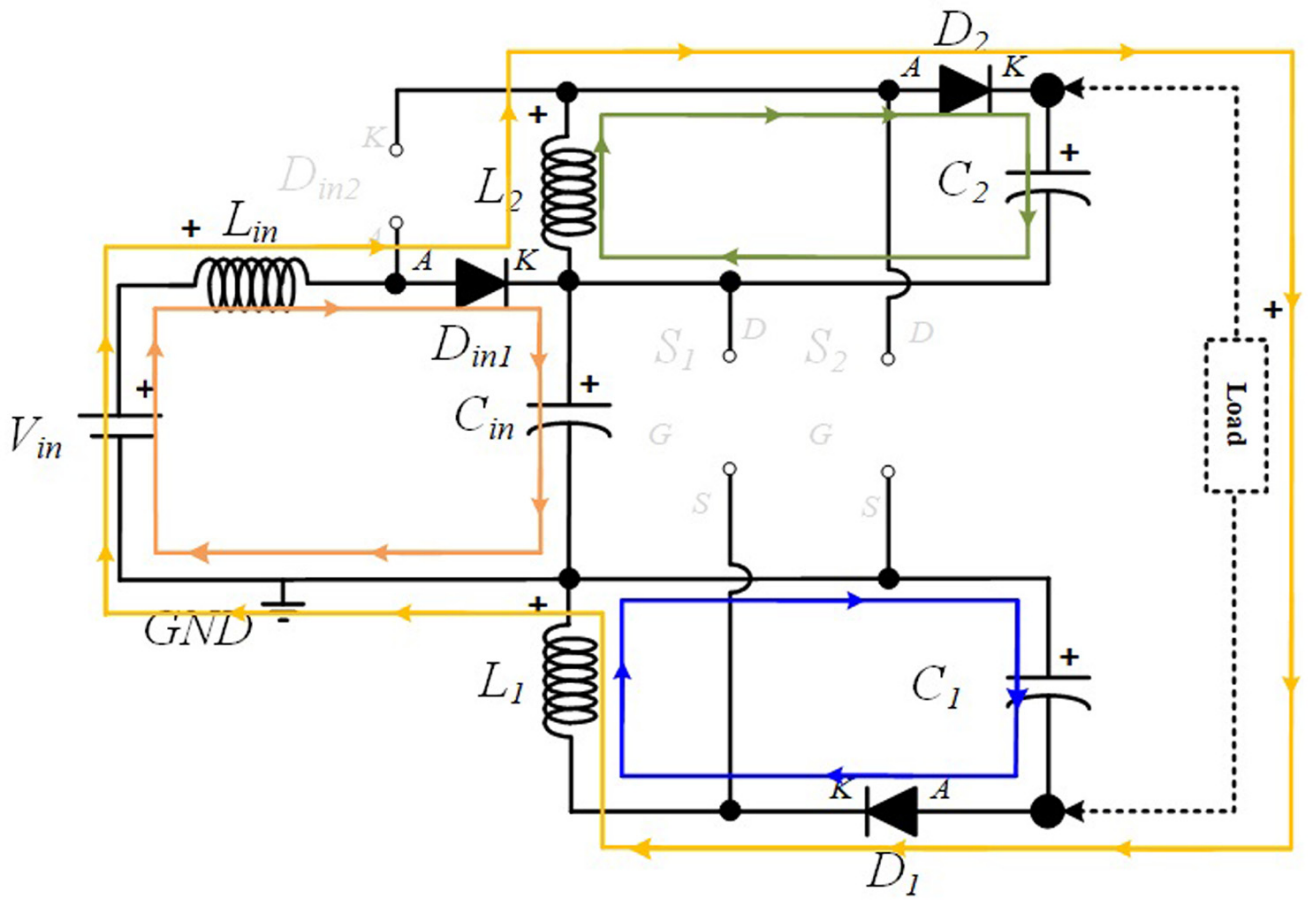
Current flow analysis when S1 and S2 are turned OFF (δ2Ts).

***S1 OFF and S2 OFF ( *δ2Ts*).*** In this operating mode, *D _in_*_*2*_ remains turned off while Din1 remains turned ON. Similar to the previous operating mode, *L _i_*_*n*_ is still discharging and Cin in charging mode. Both *D*_*1*_ and *D*_*2*_ are now turned ON since both inductors, *L*_*1*_ and *L*_*2*_, are discharging the accumulated energy over *C*_*1*_ and *C*_*2*_, respectively. The current paths described can be found in Figure 4.

**Figure 5.**
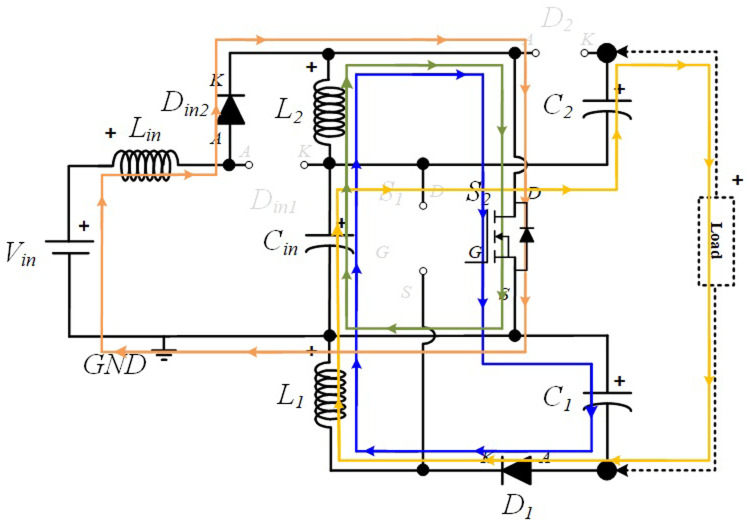
Current flow analysis when S1 is turned OFF and S2 is turned ON (δ3Ts).

***S1 OFF and S2 ON ( *δ3Ts*).*** In this operating mode, after turning ON the switch *S*_*2*_, *D _in_*_*2*_ turns ON to flow the current over the input inductor *L _i_*_*n*_, while *D _in_*_*1*_ turns OFF due to reverse voltage. Thus, *L _i_*_*n*_ is charging, and *C _i_*_*n*_ is discharging the accumulated energy in the previous operating mode over the inductor *L*_*2*_, which is storing energy. As a consequence of passive components polarity, *D*_*2*_ becomes reverse-biased and turned off, while *C*_*2*_ starts to discharge over the load. In the opposite direction, *D*_*1*_ is forced to turn ON to discharging the energy accumulated over the inductor *L*_*1*_ into *C*_*1*_, which is in charging mode. Figure 5 shows the representation of the current path flow described now.

***S1 OFF and S2 OFF ( *δ4Ts*).*** In this operating mode, both switches are turned off and the current’s path flow is the same as the ones presented in the interval *δ2Ts*.

**Figure 6.**
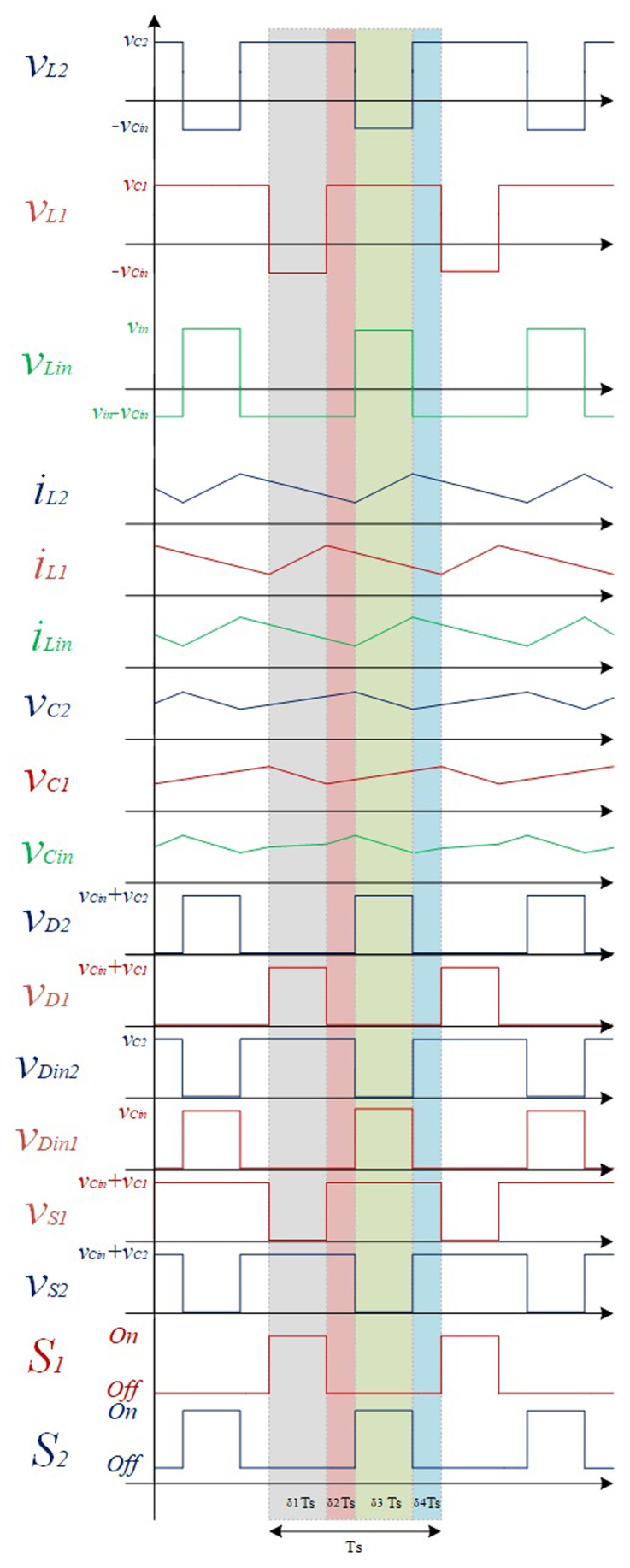
Theoretical wave forms of the proposed DC-DC Converter.

Following the operation modes described previously, it can be observed and concluded that the current in the inductor *L _1_* increases when the switch *S _1_* is turned ON and decreases when *S _1_* is turned OFF. This means that the switching state of *S _1_* does not affect the current in *L _in_* and *L _2_*. On the contrary, the current in the inductors *L _in_* and *L _2_* increase when switch *S _2_* is turned ON and decreases when *S _2_* is turned OFF. This means that the switching state of *S _2_* does not affect the current in *L _1_*. This indicates a partially independent operation of the two power switches, when the switching states of *S _1_* and *S _2_* do not overlap. According to the principle of operation detailed in the previous subsection, it is possible to obtain the theoretical waveforms of the four-operating mode of the proposed converter (see Figure 6). When analysing the evolution of the voltage across each inductor and semiconductor presented in this figure is possible to establish the voltage relationships shown in Table 1.

**Table 1.** Voltage relationship between components.

*Voltage/Time*	*(δ _1_Ts)*	*(δ _3_Ts)*	*(δ _2_ Ts, δ _4_Ts)*
*v _L1_*	*-v _Cin_*	*v _C1_*	*v _C1_*
*v _L2_*	*v _C2_*	*-v _Cin_*	*v _C2_*
*v _Lin_*	*v _in_ - v _Cin_*	*v _in_*	*v _in_ - v _Cin_*
*v _Din1_*	*0*	*v _Cin_*	*0*
*v _Din2_*	*v _C2_*	*0*	*v _C2_*
*v _D1_*	*v _Cin_ + v _C1_*	*0*	*0*
*v _D2_*	*0*	*v _Cin_ + v _C2_*	*0*
*v _S1_*	*0*	*v _Cin_ + v _C1_*	*v _Cin_ + v _C1_*
*v _S2_*	*v _Cin_ + v _C2_*	*0*	*v _Cin_ + v _C2_*

According to Table 1, as result of the analysis of the voltage relationships between components is possible to see that the maximum voltage stress over power devices S _1_ and S _2_ are v _Cin_ + v _C1_ and v _Cin_ + v _C2_, respectively, which is far reduced when compared to most DC-DC converters whose power devices must support the maximum output voltage.

In this way, it is possible to establish the following voltage relationships for each inductor. Assuming ideal components and considering one switching cycle, the relationship between the output and input current, function of the duty cycle, can be obtained through the volt-second relationship of the inductors *L _1_*, *L _2_* and *L _in_*, as presented from ( 1) to ( 3), respectively:


$$\;\delta_{1}(-v_{Cin})=(\delta_{2}+\delta_{3}+\delta_{4})(v_{C1})\;(1)$$

$$\;\delta_{3}(-v_{Cin})=(\delta_{1}+\delta_{2}+\delta_{4})(v_{C2})\;(2)$$

$$\;\delta_{3}(v_{in})=(\delta_{1}+\delta_{2}+\delta_{4})(v_{in}-v_{Cin})\;(3)$$

Knowing that *(δ2 + δ3 + δ4) = (1 – δ1)* and *(δ1 + δ3 + δ4) = (1 – δ3)*, as well as *δ1 = δ3 = δ*; equalizing and solving the Equation (1) to Equation (3) to each capacitor voltage, it is possible to establish the voltage equations listed below from ( 4) to ( 6):


$$\;v_{C1}=\frac{\delta }{1-\delta }v_{Cin}\;(4)$$

$$\;v_{C2}=\frac{\delta }{1-\delta }v_{Cin}\;(5)$$

$$\;v_{Cin}=\frac{1}{1-\delta }v_{in}\;(6)$$

Equalizing and solving Equation (4) to Equation (6) in order to *Vin*, and knowing that *Vout = vCin + vC1 + vC2*, it is possible to establish the expression that characterizes the voltage gain of the proposed interleaved quadratic DC-DC converter ( 7):


$$\;v_{out}=\frac{1+\delta }{{\left(1-\delta \right)}^{2}}v_{in}\;(7)$$

### Design considerations

In this section, the entire design process of the passive components used in the proposed experimental prototype will be presented and discussed. The following characteristics were considered in the design of the prototype: *v _in_* = 50 V, *δ _max_* = 0.5, *R _Load_* = 450 Ω, *P _out(max)_* = 200 W, *v _out_* ( *δ _max_*) = 300 V, *i _out_* ( *δ _max_*) = 300/450 = 0.67 A, *Δi _Lmax_* = 0.5 A, *Δv _Cmax_* = 1 V to 3 V, *f _PWM_* = 50 kHz, efficiency of 95%.

### Inductors design

For the inductors design, the generic adopted expression to define the minimum inductance value is presented in ( 8). This expression is based on the linear variation of the current in the inductor and is well explained in most design chapters about DC-DC converters, such as 48– 50.


$$\;L>\frac{v_{L_{max}}\cdot \delta }{f_{PWM}\cdot \Delta i_{L}}\;(8)$$

Where *v _Lma_*_*x*_ is the maximum voltage applied to the inductor, *δ* is the maximum duty cycle intended for the converter, *f _PWM_* is the switching frequency of the converter, and *Δi*_*L*_ is the maximum current variation (ripple) desired in the inductor. For the input inductor, *L _i_*_*n*_, the following equation can be used:


$$L_{in}>\frac{v_{in}\cdot \delta }{f_{PWM}\cdot \Delta i_{L}}\Rightarrow L_{in}>1mH\;(9)$$

For the remaining inductors, *L _1_* and *L*_*2*_, the following equation can be used:


$$L_{1}=L_{2}>\frac{v_{Cin}\cdot \delta }{f_{PWM}\cdot \Delta i_{L}}\Rightarrow \frac{\frac{v_{in}}{\left(1-\delta \right)}\cdot \delta }{f_{PWM}\cdot \Delta i_{L}}>2mH\;(10)$$

### Ferromagnetic material saturation analysis

The material of the inductors, applied in this prototype, uses a Litz 420x0.08 SE F155 G1 wire type (widely used in high frequency applications, as it reduces losses and the skin effect), a plastic inductor winding support from the CF model -E70-1S and a set of ferrite cores from model E70/33/32DG in “U” shape, from the manufacturer TDK, with type N87 ferrite. Using the manufacturer datasheet, it is possible to obtain some essential parameters (see Table 2) for analyzing the electromagnetic saturation of the ferrite core.

**Table 2.** Magnetic Parameters of the Ferrite Core E70/33/32DG.

Magnetic Parameter	*Value*
Effective magnetic cross section (Ae)	683 mm ^2^
Inductance factor (AL)	250 nH
Effective magnetic length (le)	149 mm
Saturation flow density (B)	390 mT

Thus, to calculate the maximum current value that can cross each inductor, before saturating the ferromagnetic material is possible to estimate the number of turns of each winding, taking into account the desired inductance value, *L*, (H) and the inductance factor, *A*_*L*_, (H) of the material adopted ^48– 51^.


$$\;L=N^{2}A_{L}\Leftrightarrow N=\sqrt{\frac{L}{A_{L}}}\;(11)$$

After applying Equation (11) to each inductor, the values expressed below were obtained from ( 12) and ( 13).


$$N_{L_{1}}=N_{L_{2}}=\sqrt{\frac{2\times 10^{-3}}{500\times 10^{-9}}\;}\approx 63turns\;(12)$$

$$\;N_{Lin}=\sqrt{\frac{1\times 10^{-3}}{500\times 10^{-9}}\;}\approx 45turns\;(13)$$

Then, the permeability of the ferrite adopted, μ, (H/m) can be calculated from Equation (14).


$$A_{L}=\frac{\mu \cdot A_{e}}{l_{e}}\Leftrightarrow \mu =\frac{A_{L}\cdot l_{e}}{A_{e}}=\frac{250\times 10^{-9}\cdot 149\times 10^{-3}}{683\times 10^{-6}}=5,4539\times 10^{-5}H/m\;(14)$$

Finally, using Equation (15) is possible to calculate the maximum current over each inductor prior to saturate the ferrite magnetic material.


$$\;I_{máx}=\frac{B_{máx}\cdot l_{e}}{\mu \cdot N}\;(15)$$

Applying ( 15) for all the inductors, the current values can be calculated.


$$I_{L_{1Máx}}=I_{L2_{Máx}}=\frac{390\times 10^{-3}\cdot 149\times 10^{-3}}{5,4539\times 10^{-5}\cdot 63}=16,91A\;(16)$$

$$I_{L_{in_{Máx}}}=\frac{390\times 10^{-3}\cdot 149\times 10^{-3}}{5,4539\times 10^{-5}\cdot 45}=23,67A\;(17)$$

### Capacitors design

Similarly, for the capacitors design, the generic adopted expression to define the minimum capacitance value is presented in ( 18). This expression is based on the linear variation of the voltage in the capacitors.


$$\;C>\frac{i_{C_{max}}\cdot \delta }{\Delta v_{C}\cdot f_{PWM}}\;(18)$$

Where *i _Cma_*_*x*_ is, generally, the maximum current flowing through the capacitor, *δ* is the maximum duty cycle intended for the converter, *f _PWM_* is the switching frequency of the converter, and *Δv*_*C*_ is the maximum voltage variation (ripple) desired in the capacitor. For the input capacitor is necessary to obtain the input current, which can be obtained based on the output power, efficiency and input voltage ( 19). It was selected a maximum ripple *Δv _Cma_*_*x*_ = 3 V for the input capacitor and *Δv _Cma_*_*x*_ = 1 V for the remaining capacitors.


$$C_{in}>\frac{(i_{out}-(i_{L_{1}}+i_{L_{2}}))\cdot \delta }{\Delta v_{C}\cdot f_{PWM}}\Rightarrow C_{in}>\frac{\frac{P_{in}}{v_{in}}\cdot \delta }{\Delta v_{C}\cdot f_{PWM}}\Rightarrow C_{in}>14\mu F\;(19)$$

For the capacitors *C _1_* and *C _2_*, the following Equation (20) can be used.


$$\;C_{1}=C_{2}>\frac{i_{out}\cdot \delta }{\Delta v_{C}\cdot f_{PWM}}\Rightarrow C_{1}=C_{2}>6.7\;\mu F\;(20)$$

This calculation shows that the proposed interleaved converter requires small capacitor which is an advantage over other topologies.

### Comparison with other interleaved quadratic DC-DC Boost topologies

This section is intended to compare the proposed solution with other interleaved quadratic Boost DC-DC topologies presented in the references ^52– 55^. Table 3 shows a comparison about the number of components needed to achieve the voltage gain obtained by each converter and maximum voltage stress over the power devices.

**Table 3.** Comparison between some different interleaved quadratic Boost DC-DC topologies.

	*Topologies*					
	*Proposed*	*52*	*53*	*54*	*55*	*56*
*Number of Switches*	*2*	*1*	*2*	*2*	*2*	*4*
*Number of Diodes*	*4*	*3*	*6*	*4*	*6*	*2*
*Number of Inductors*	*3*	*2*	*4*	*4*	*4*	*4*
*Number of Capacitors*	*3*	*2*	*4*	*3*	*3*	*2*
Voltage gain *	$\frac{1+\delta }{{\left(1-\delta \right)}^{2}}$	$\frac{1}{{\left(1-\delta \right)}^{2}}$	$\frac{2}{{\left(1-\delta \right)}^{2}}$	$\frac{\left(1+n)(2-d\right)}{{\left(1-\delta \right)}^{2}}$	$\frac{1}{{\left(1-\delta \right)}^{2}}$	$\frac{2n+2}{{\left(1-\delta \right)}^{2}}$
*Maximum Voltage stress over switches*	$\frac{V_{out}-\delta }{{\left(1-\delta \right)}^{2}}V_{in}$	*V _out_*	$\frac{V_{out}}{2}$	$\frac{V_{in}}{{\left(1-\delta \right)}^{2}}$	*V _out_*	$\frac{V_{out}}{2(n+1)}$

* n-winding ratio between coupled inductors

The comparison between these topologies shows that the solution proposed in this paper is not the one that achieves the highest voltage gain, but presents a relative high voltage gain with less components and is one that presents the most reduced voltage stress over the power devices.

**Figure 7.**
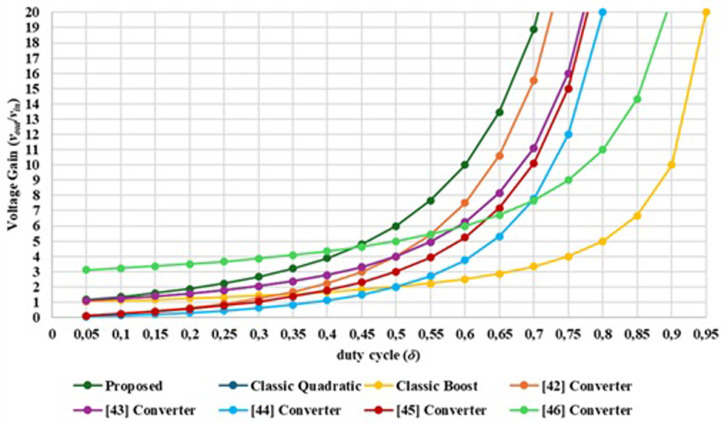
Comparison between the proposed interlevead quadratic DC-DC converter other similar topologies regarding the voltage gain versus the duty cycle.

Figure 7 compares the theoretical voltage gain characteristics of each DC-DC Boost converter topology. As shown in Figure 7, the proposed converter is able to provide a voltage gain output of six times the input voltage for δ = 0.5. The solution presented in 56 presents the best voltage gain bellow 0.45 but the voltage gain rate above 0.45 is smaller than the proposed topology.

### Laboratory validation

**Figure 8.**
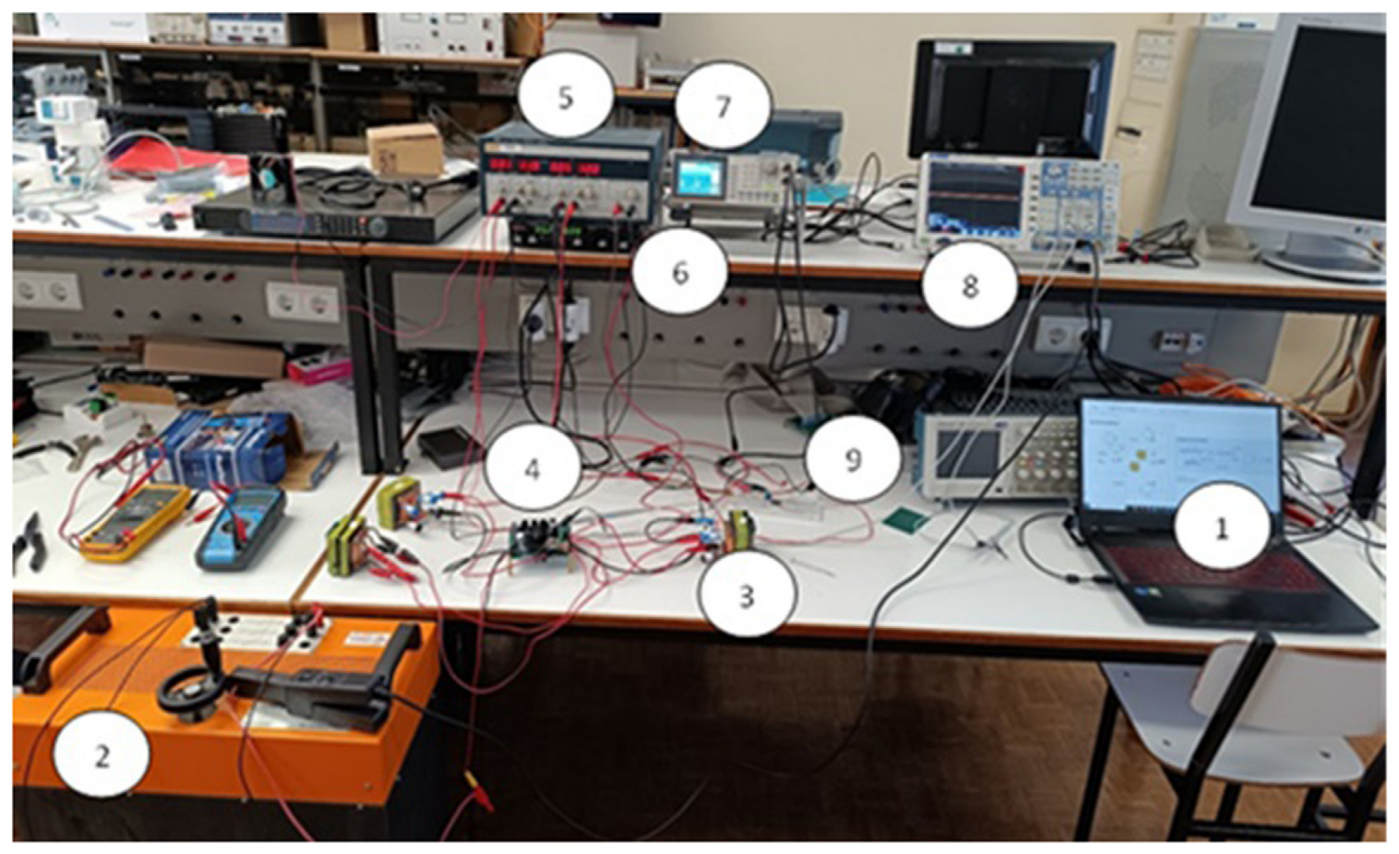
Workbench with the proposed DC-DC converter prototype (1–Laptop with the circuit simulation in MATLAB®/SIMULINK®; 2 – Load resistor; 3 – Inductor; 4 – Printed Circuit Board (PCB) of the power circuit and gate drive circuit; 5 – Power supply for the control circuit; 6 – Power Supply for the power circuit; 7 – Signal generator; 8 – Oscilloscope; 9 – Breadboard with the PWM control circuit).

The practical tests of the proposed converter prototype were carried out considering a maximum output power of 200 W, using a constant load resistance of 450 Ω, an input voltage of 50 V and a switching frequency of 50 kHz. The inductors are *L _1_=L _2_=2mH* and *L _in_=1mH*. The capacitors are *C _in_=22μF* and *C _1_=C _2_=10μF* (normalized values). It should be noted that all practical results were always compared with a computational validation of the converter circuit operation, employing the MATLAB ^®^/SIMULINK ^®^ software. Figure 8 shows a wide-angle photograph of the workbench with the proposed converter prototype and all the essential devices to test the solution.

### Inductors and power devices waveforms

**Figure 9.**
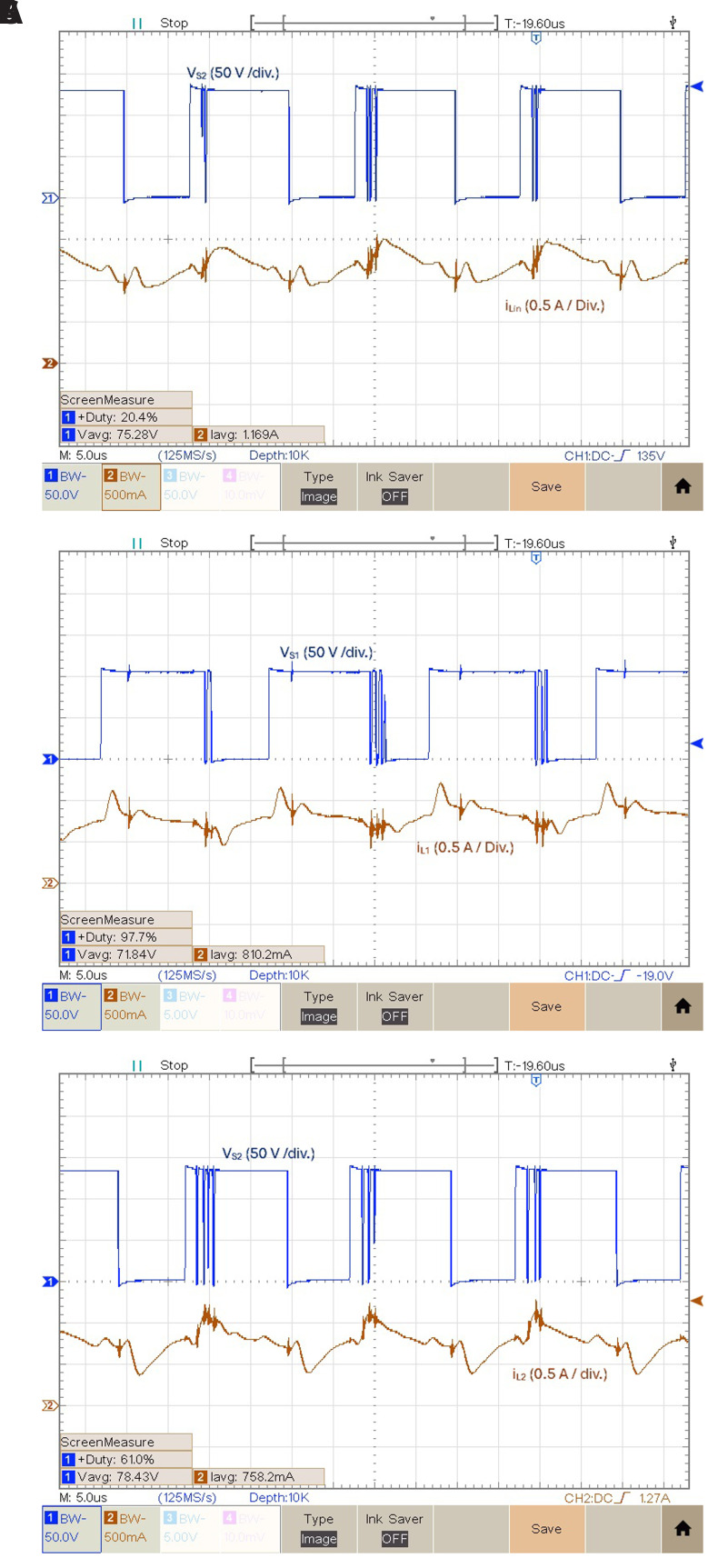
Inductor currents and power devices voltages for *δ* = 0.4: **a**) iLin and vS2; **b**) iL1 and vS1; **c**) iL2 and vS2.

Figure 9 shows the experimental result of the three inductor currents obtained from the power circuit of the proposed converter for δ = 0.4 and relation with the switching of the power devices. The results presented in Figure 9 confirm the dependence between each inductor and one of the power switches, specifically, the state of charge of *L _in_* and *L _2_* depends on the conduction state of S2, and the same situation occurs between *L _1_* and *S _1_*. The mean current values are: *iL _in_* = 1.169 A, *iL _1_* = 810.2 mA and *iL _2_* = 758.2 mA.

### Diodes and power devices waveforms

**Figure 10.**
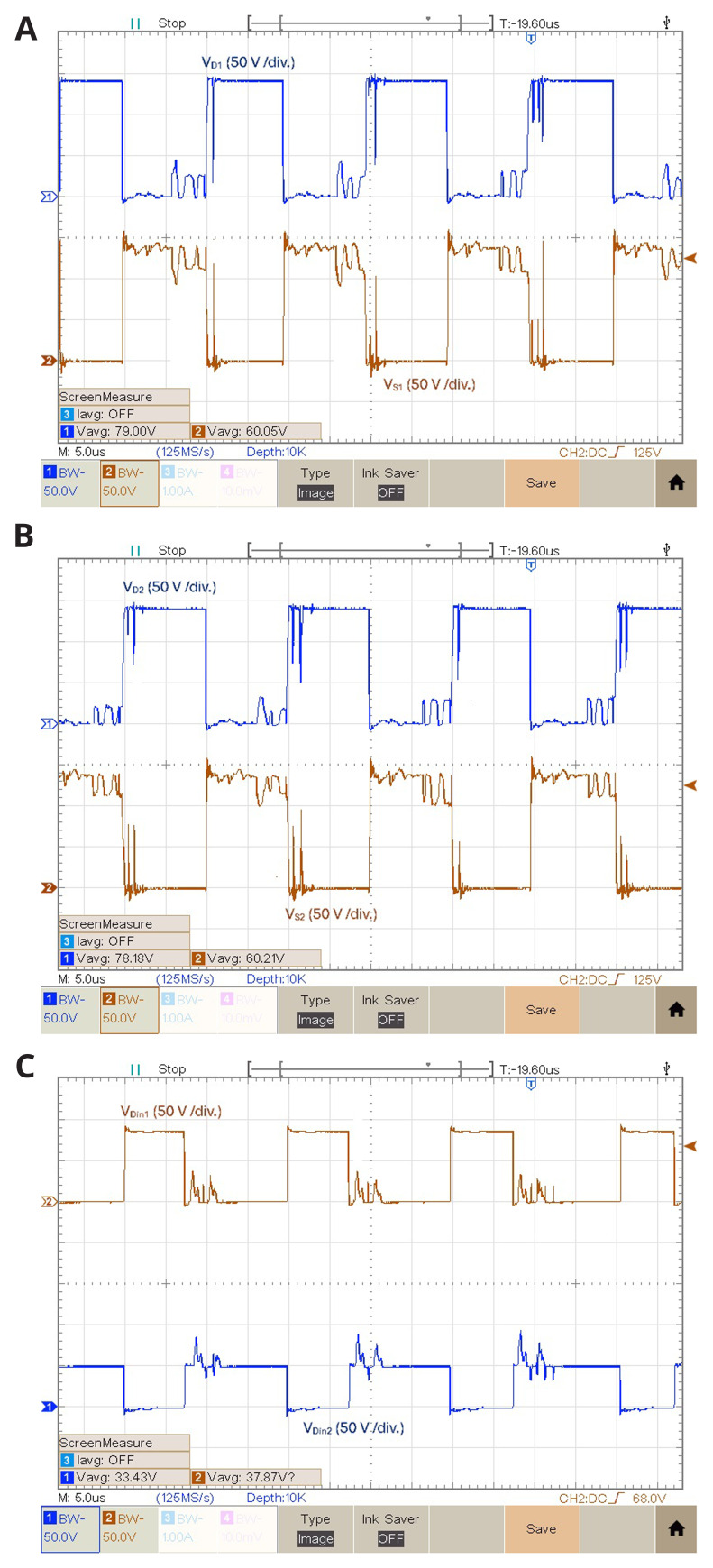
Diodes and power switches voltages for *δ* = 0.4: **a**) vD1 and vS1; **b**) vD2 and vS2; **c**) iLin , vDin1 and vDin2.

Figure 10 illustrates the experimental results of the relationship between the diodes and the power switches voltage for *δ* = 0.4. According to the results shown above ( Figure 10a) and Figure 10b)), the conduction states of *D _1_* and *D _2_* are symmetric to the conduction state of *S _1_* and *S _2_*, respectively. On the other hand, the conduction states of both input diodes *D _in1_* and *D _in2_* are dependent on the *S _2_* (see Figure 10c)) state, just like *i _Lin_*, as concluded before in Figure 8a). Notice that the power devices voltage waveforms are different depending on the experimental result for the same duty cycle, which is probably due to coupling of common mode noise, which is more intensive as the switching frequency increases.

### Capacitors and power devices waveforms

**Figure 11.**
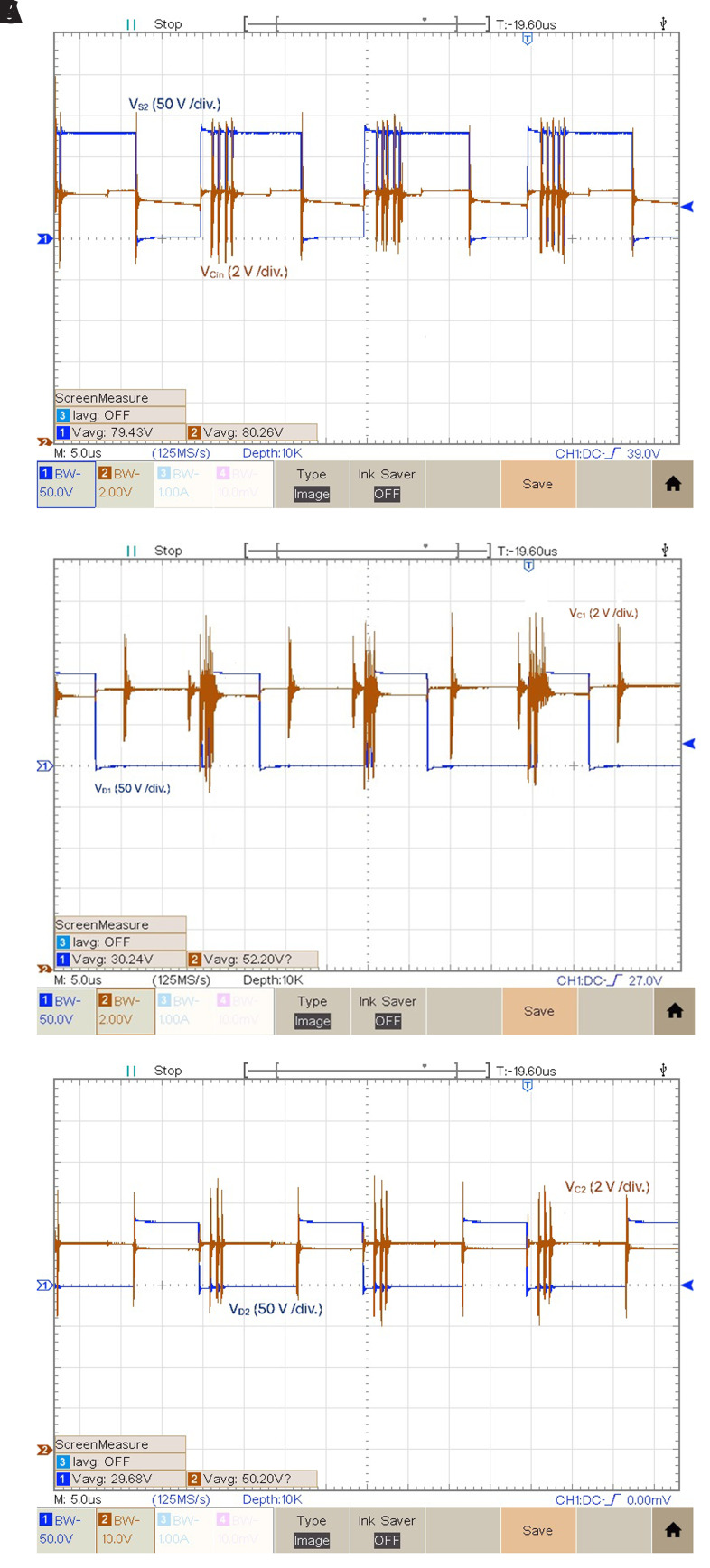
Capacitor and power switches voltages for *δ* = 0.4: **a**) vCin and vS2; **b**) vC1 and vD1; **c**) vC2 and vD2.

Figure 11 shows the experimental results of each capacitor and the power switches voltages for *δ* = 0.4. Interpretating the results shown in Figure 11, and remembering the symmetric relation between *D _1_* and *S*_*1*_, along with *D*_*2*_ and *S*_*2*_, it is clear the correspondence between the state of charge of both *C _i_*_*n*_ and *C*_*2*_ and *S*_*2*_, as well as, between *C*_*1*_ and *S*_*1*_. Making a parallel analysis between the results of Figure 9 and Figure 11, it is also possible to observe when an inductor is discharging the corresponding capacitor is charging and vice-versa. The mean voltage values are: *v _Ci_*_*n*_ = 80.26 V, *v _C_*_*1*_ = 52.20 V and *v _C_*_*2*_ = 50.20 V. Notice that in the following figure, the Ch2 voltage gain is only 2V/div, and the reference is virtually several divisions bellow the minimum visible at the screen (this Peaktech oscilloscope allows this configuration). Thus, the noise is not so high as it seems at first sight.

### Output voltage and voltage gain

**Figure 12.**
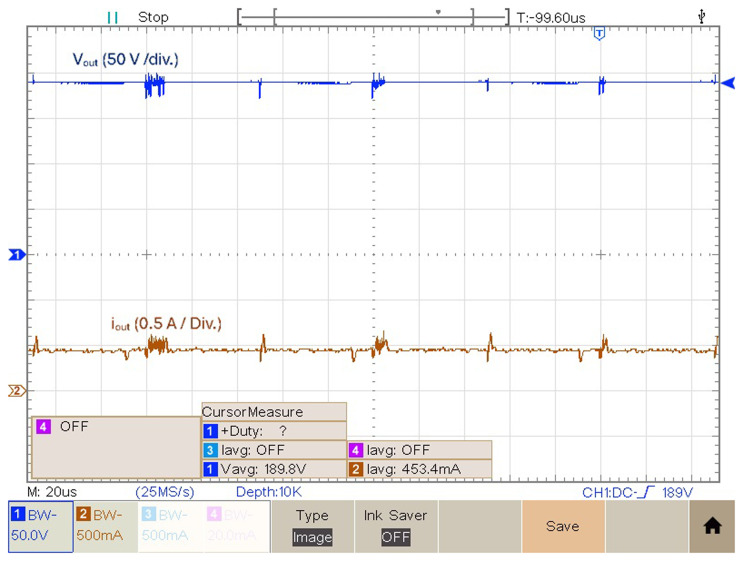
Output voltage and current for a duty cycle, *δ* = 0.4.

In this subsection several experimental results are presented of the output voltage, output current and a voltage gain comparison for different duty cycles. Figure 12 features the experimental voltage and current output result obtained with *δ* = 0.4. Observing Figure 12 it is possible to see that for *δ* = 0.4 the proposed prototype is able to produce an output voltage *v _out_* = 189.8 V, which translates to a voltage gain ( *v _out_/v _in_*) of 3.66. The mean value of the output current is equal to 453.4 mA.

**Figure 13.**
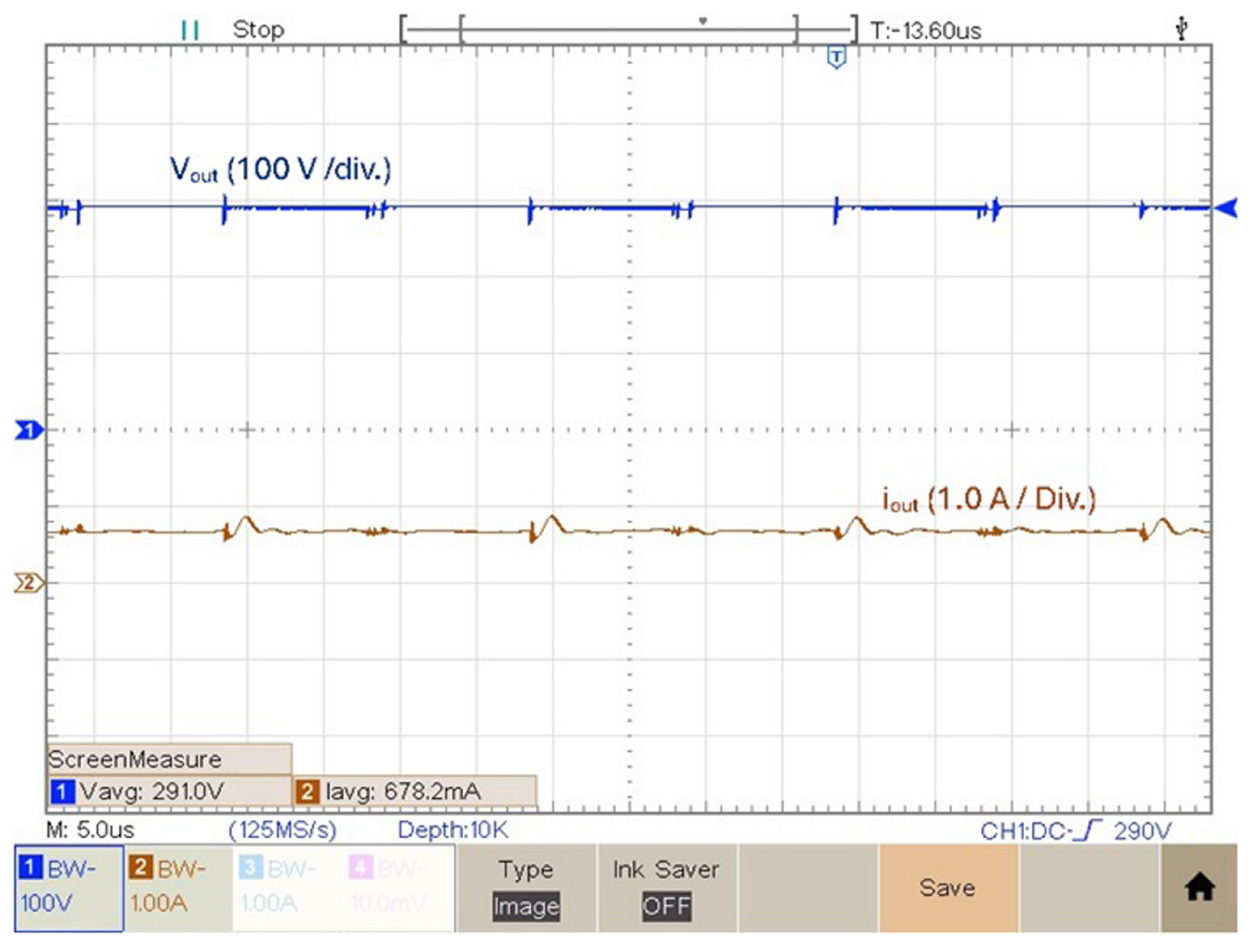
Output voltage and current for a duty cycle, *δ* = 0.5.

Figure 13 shows the experimental result of the output voltage and output current obtained in the prototype for *δ* = 0.5. Observing Figure 13 with this duty cycle is possible to observe an output voltage *v _ou_*_*t*_ = 291.00 V, which translates to a voltage gain ( *v _out_/v _i_*_*n*_) of 5.82. The mean value of the output current is equal to 690.6 mA.

**Figure 14.**
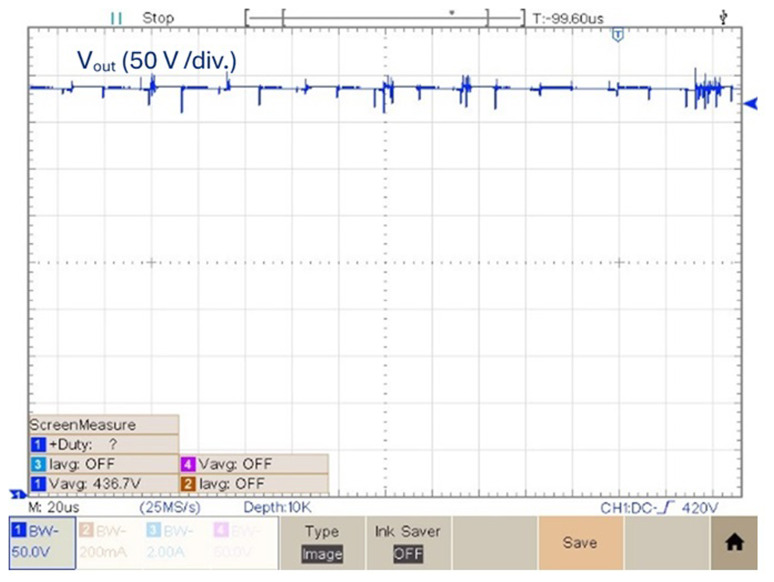
Output voltage for a duty cycle, *δ* = 0.6.

As a final experimental result, the prototype was tested with a duty cycle value *δ* = 0.6. Figure 14 shows the output voltage in this condition. According to Figure 14, the experimental result obtained of the output voltage obtained with a duty cycle *δ* = 0.6 is 436.7 V. Which means it is possible to get a voltage gain ( *v _out_/v _i_*_*n*_) of 8.62. Above this duty cycle is difficult to increase the voltage gain due to increased losses.

## Results

**Figure 15.**
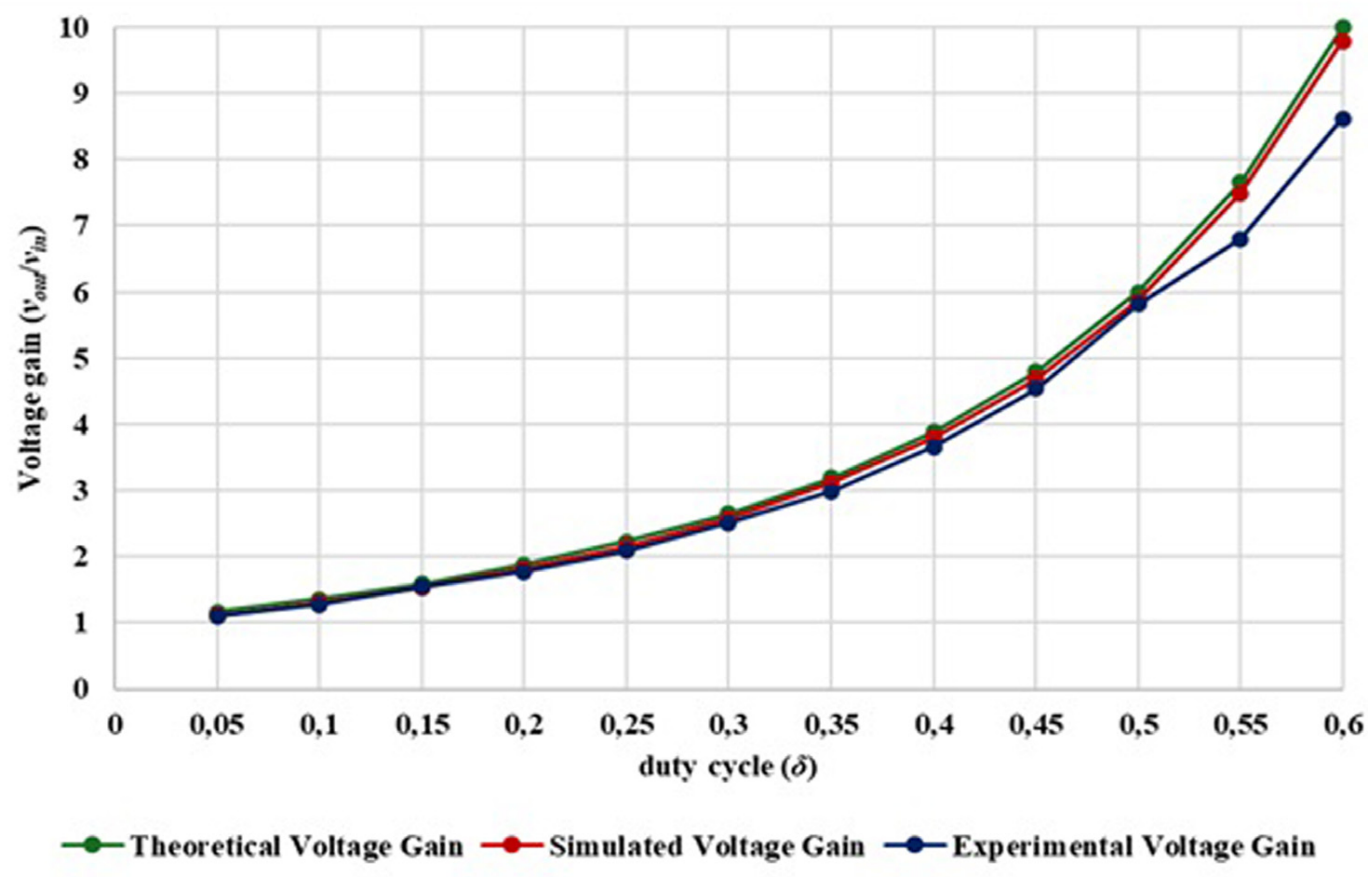
Comparison between theoretical, simulation and experimental voltage gain.

This section discusses the results obtained based on the experimental test observations, oscilloscope waveforms, and measured voltages and currents. Figure 15 compares the theoretical voltage gain of the proposed converter, the computational simulation results (simulated in *MATLAB ^®^/SIMULINK ^®^* using the average power losses available in the *Simscape Power Systems toolbox*) and also the experimental voltage gain obtained.

Analyzing Figure 15 is possible to observe in the duty cycle range from 0.05 to 0.60, the output voltage and voltage gain of the experimental prototype shows a high degree of similarity when compared with the simulation and theoretical calculations. Additionally, it is confirmed that with a duty cycle of 0.20, a gain voltage greater than 1.7 is achieved, with a duty cycle of 0.30, a voltage gains greater than 2.5, with a duty cycle of 0.40, a voltage gains greater than 3.6 and with a duty cycle of 0.50, a gain greater than 5.8 is achieved. A maximum gain of 8.62 was achieved with a duty cycle of 0.60. Behind this duty-cycle is difficult to improve the voltage gain since the losses become extremely high.

**Figure 16.**
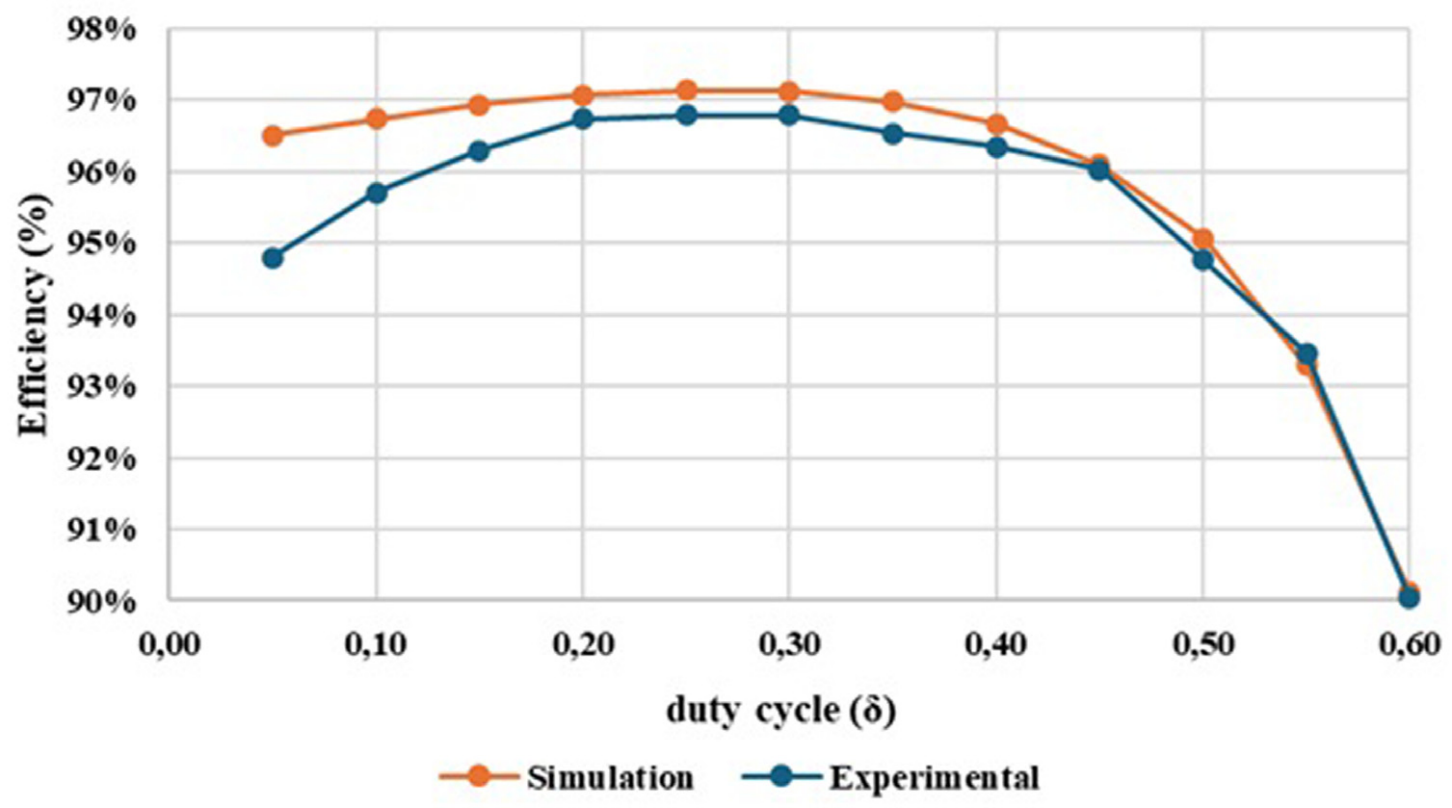
Comparison between the simulation and the experimental results concerning the converter efficiency.

Figure 16 shows the graphical result of the efficiency obtained in both simulation and experimental tests, as function of the converter duty cycle.

After analysing the results presented in Figure 16, a close correlation is observed between the efficiency variation and the duty cycle applied to the converter. For a duty cycle variation from 0.05 to 0.60, an efficiency range around 97% to 90% was obtained in the simulation tests and an efficiency range around 96% to 90% was obtained in the experimental tests. It is also observed that a maximum efficiency of 96.79% was achieved for a duty cycle δ = 0.25.

**Figure 17.**
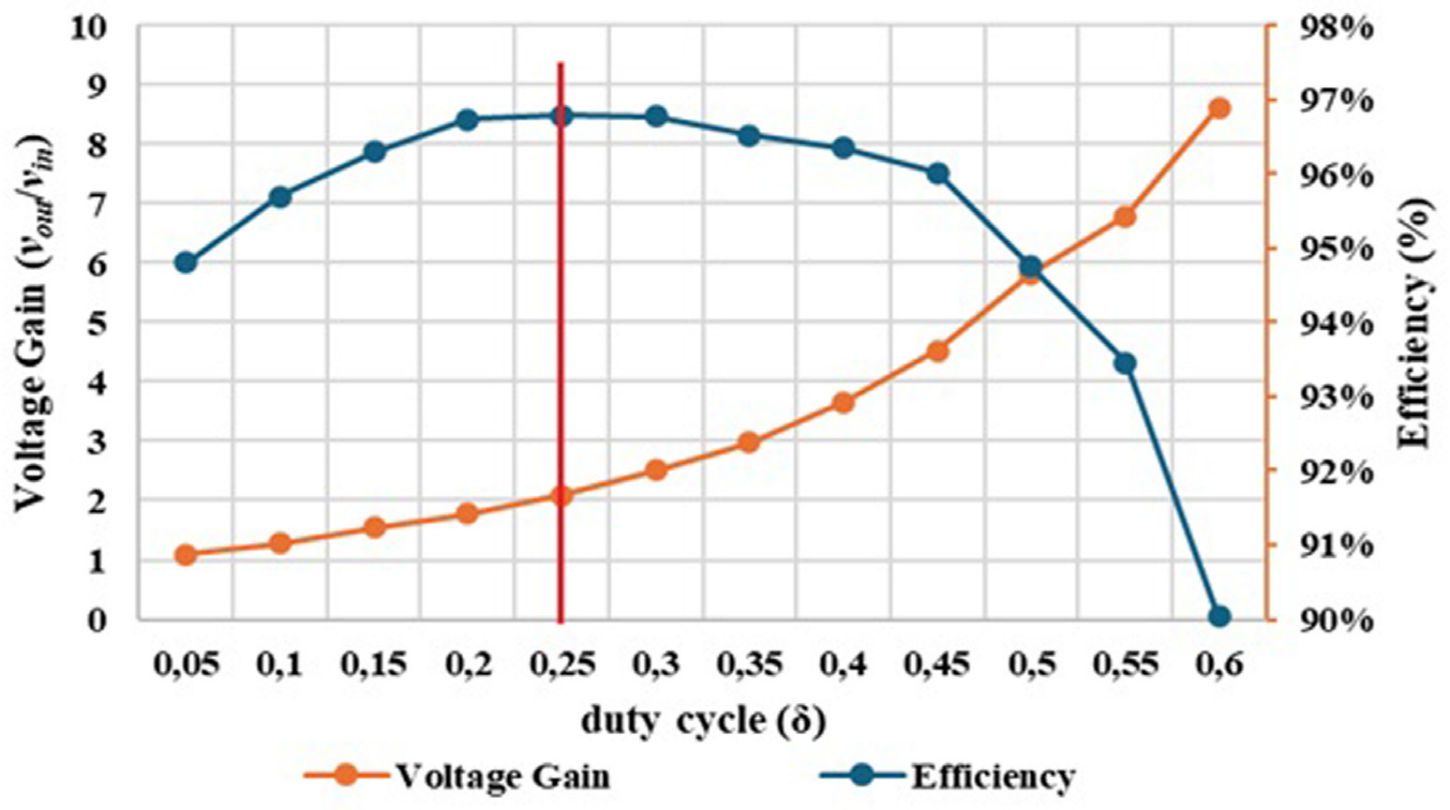
Comparison between experimental voltage gain result and experimental efficiency result. A maximum efficiency of 96.79% was achieved for a duty cycle around δ = 0.25.

Figure 17 illustrates the evolution of the experimental efficiency and voltage gain over the duty cycle. The purpose of this relationship is to evaluate at which output voltage gain value it is possible to achieve the best efficiency, helping us to identify an optimal operating point for the proposed DC-DC converter prototype.

Examining Figure 17 is possible to observe that the optimal operating point of the converter happens with a duty cycle of δ =0.25 which results in a voltage gain of 2.09 (marked in red in the figure). However, it is clear that, up to a duty cycle of 0.60, the converter maintains an efficiency between 90% and 96%, which can be considered quite satisfactory. At the maximum value for which it was designed, with a duty cycle of 0.50, the converter presents a voltage gain of 5.82 and an efficiency of 94.76%. Additionally, there is very little variation in efficiency, as it remains between 96% and 95% until reaching a duty cycle of 0.50.

**Figure 18.**
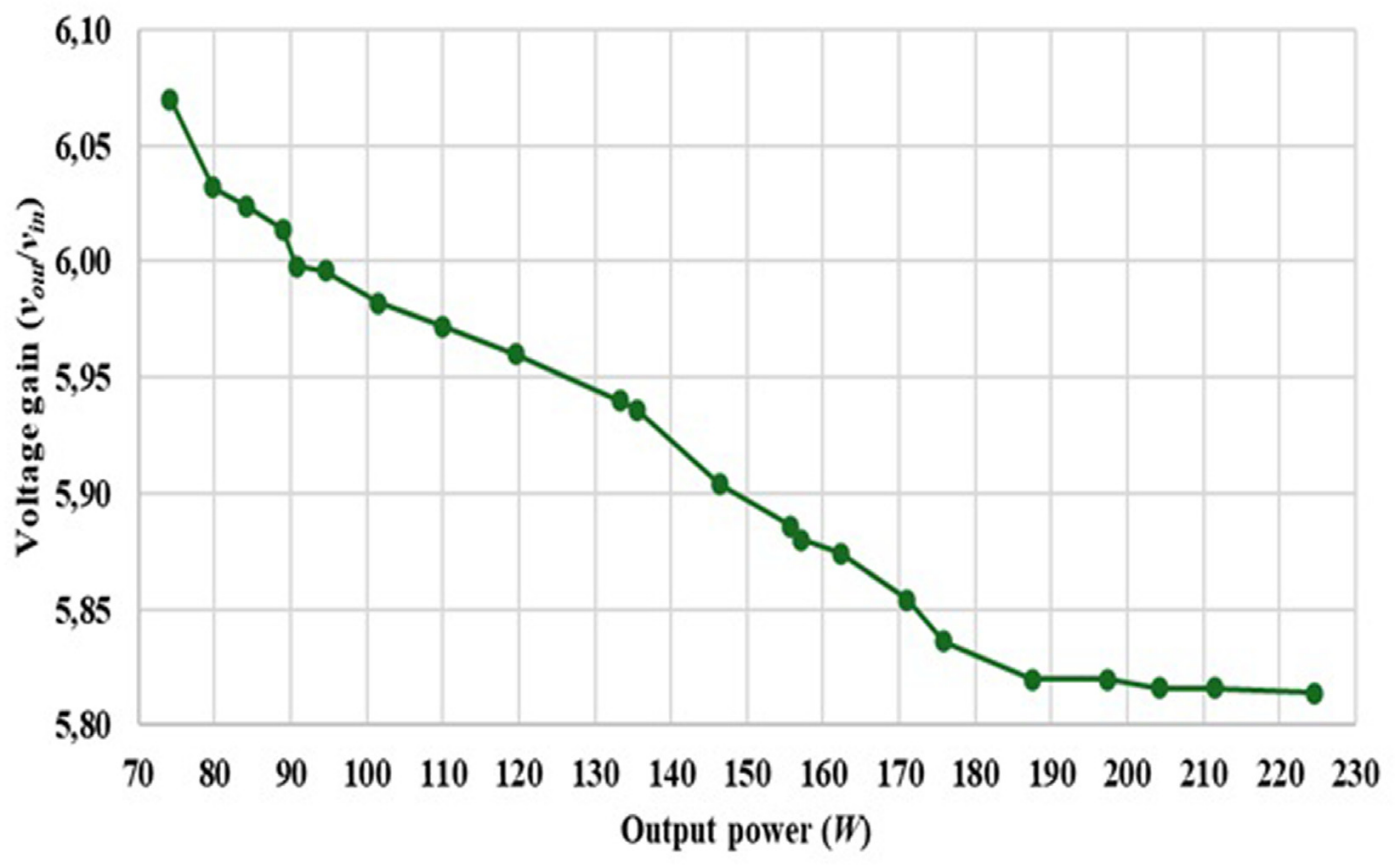
Output voltage gain versus Output Power, considering a fixed duty cycle of 0.50.

Finally, Figure 18 shows the relation between the output power and the output voltage gain of the proposed topology considering a fixed duty cycle of 0.50, showing certain limitations over the output gain due to the several losses.

## Discussion

DC-DC converters play an important role in the integration of different Renewable Energy Sources (RES) into DC distribution networks or as front end devices to connect to DC-AC converters, according to the desired requirements and adopted equipment interface. There is a constant research regarding the design of new DC-DC converter topologies with high-voltage gain ratio and boost ability to extend the operation of RES, and other sources, all over the available voltage ranges, extracting efficiently as much energy as possible. In this paper it was made a brief research about other type of DC-DC converters and it was decided to create a new topology interleaved quadratic DC-DC converter. Quadratic DC-DC converter are some of the topologies that can achieve high voltage gains and are the most suitable for several RES applications due to the variability of most of them, which are dependent on weather conditions, location, distribution system and other aspects. When compared with other topologies in the literature, especially other quadratic DC-DC converters, the proposed topology is the one with higher voltage gain, but present a reduced number of components and the interleaved solution allows to reduce the voltage and current stress over power devices which allows to increase the reliability of the solution. The laboratory prototype was tested in several conditions during several days to evaluate the overall performance, namely the voltage and current stress, robustness, overheating issues, hot spots, electromagnetic noise, efficiency, sensitivity to parameters variation and other aspects. Regarding the electromagnetic noise, some adjustment need to done as future work in the printed circuit board and components, but the overall performance is quite acceptable. This will allow to improve some waveform and interference due to electromagnetic noise. The efficiency was measured in several conditions and real values between 90% (worst conditions) and 96% (best condition) were achieved. Other relevant aspect is the reduced value of capacitors, due to the interleaved operation. This leads to reduced stress over capacitors and distributed voltage. Notice that the output voltage is the sum of the voltage over the three capacitors. A low power laboratory prototype was developed but is possible develop a similar high power converter.

## Conclusions

This paper proposed a new interleaved quadratic DC-DC boost converter topology with high-voltage gain, confirming the theoretical operation principle based on some experimental setup. The experimental results demonstrate that the proposed topology allows higher voltage gains than most well-known quadratic topologies, reaching a voltage gain from six to eight in a real prototype without degrading the efficiency significantly. The proposed converter provides continuous input and output current and a simple PWM control strategy. Furthermore, it was possible to optimize the converter's efficiency by adjusting the duty cycle, which plays a crucial role in minimizing conduction and switching losses. The obtained results indicate that there is an optimal operating point where efficiency is maximized, achieving an efficient balance between voltage gain and associated losses. Additionally, there is a very small variation in efficiency, which remains between 95% and 96% up to a duty cycle of 0.50, where a voltage gain of 5.82 is achieved in a real setup. However, when the voltage gains increase behind this point, the efficiency began to decrease, resulting in an evident trade-off between maximizing voltage gain and energy efficiency. This trade-off is particularly relevant in solar photovoltaic applications, where it is necessary to find an appropriate compromise between voltage gain and efficiency based on the specific requirements of the system.

## Ethics and consent

Ethical approval and consent were not required.

## Data Availability

No data associated with this article.
