# Interleaved Quadratic Boost DC-DC Converter with Extended Voltage Gain and Reduced Switch Voltage Stress for Photovoltaic Applications

**DOI:** 10.12688/openreseurope.19625.2

**Published:** 2025-10-28

**Authors:** Daniel Ferreira, Armando Cordeiro, Paulo Gambôa, Luis Rocha, Filipe Barata, José Fernando Silva, João F. Martins, Vitor Fernão Pires

**Affiliations:** 1 PolyTechnic University of Lisbon, Department of Electrical Engineering Energy and Automation, Instituto Superior de Engenharia de Lisboa (ISEL), Rua Conselheiro Emídio Navarro, Lisboa, 1, 1959-007, Portugal; 2 Department of Electrical and Computer Engineering (DEEC), Nova University of Lisbon, Faculdade de Ciência e Tecnologia (FCT), 2829-516 Caparica, Portugal, CTS-UNINOVA and LASI, Portugal, LASI, Portugal; 3 Polytechnic Institute of Setubal, Department of Electrical Engineering (DEE), Escola Superior de Tenologia de Setúbal, Campus do IPS, Estefanilha, Setúbal, 2914-508, Portugal; 4 INESC-ID Lisboa, Rua Alves Redol, Lisboa, 9, 1000-029, Portugal; 5 Department of Electrical and Computer Engineering (DEEC), University of Lisbon, Instituto Superior Técnico, Av. Rovisco Pais, Lisboa, 1, 1049-001, Portugal

**Keywords:** DC-DC Converters; Interleaved Quadratic Boost; High-Voltage Gain; High Efficiency; Photovoltaic systems

## Abstract

**Background:**

DC-DC power converters are essential devices in the modern world, playing a crucial role in managing the power supply from different power sources converting and adapting voltage levels. These power converters are fundamental to numerous applications, from charging your mobile phone to powering different types of machinery. Lately, due to climate change problems and the floating nature of most renewable power sources, they are essential to a carbon-free world and zero emissions target.

**Methods:**

Our investigation method was based on an initial theoretical approach using mathematical equations to describe the operation of the electrical circuit and evaluate the performance compared to other topologies, followed by the validation through some computational simulations using MATLAB/SIMULINK software. Next, the operation of the proposed converter was also confirmed by several experimental tests using a laboratory prototype developed exclusively for these tests.

**Results:**

Based on the achieved results, an efficiency analysis was performed showing that in addition to high-voltage gain, from the range of six to eight times the input voltage, the converter maintains a very high efficiency, around 95% to 96% up to a duty cycle of 0.50, where a voltage gain of 5.82 is achieved in a real setup Also, the optimal operating point was identified, based on the duty cycle, where the converter operates at maximum efficiency. In PLECS® simulation environment, dynamic tests under PI output voltage control revealed fast transient response and good voltage regulation, while MPPT PV simulations demonstrated effective maximum power extraction and tracking under variable irradiance and temperature conditions.

**Conclusions:**

In conclusion, it is possible to claim that the proposed converter presents a stable and efficient operation and has a very high potential for applications that require high-voltage gain, such as photovoltaic solar systems or even electrical vehicles or energy storage systems. Other relevant aspect is the reduced value of capacitors, due to the interleaved operation, leading to reduced stress over capacitors and distributed voltage over them.

## Plain language summary

This paper shows the study, development, and results of a new Direct-Current to Direct-Current (DC-DC) electric power converter topology, designated as interleaved quadratic Boost DC-DC converter topology. The converter topology is capable of achieving significantly higher voltage gains (higher voltage in the output when compared with the input voltage) than most conventional existing topologies. A theoretical approach was introduced in this paper and then validated through some computer simulations (using MATLAB/SIMULINK software). Finally, the performance of the topology was also confirmed in an experimental setup using a practical prototype of the proposed converter. From the experimental results, it was possible to achieve a maximum output voltage gain of over eight times the input voltage. An efficiency analysis (allowing us to identify the energy losses during the operation of the converter) was also performed, showing that the proposed topology converter maintains a very high efficiency, around 95% to 96%. The optimal operating point was also identified, based on the duty cycle (turn-on and turn-off of the power devices at a certain frequency), where the converter operates at maximum efficiency. The results show that the proposed converter has a very high potential for applications that require high-voltage gain, such as photovoltaic solar systems or even electrical vehicles or energy storage systems.

## Introduction

The increasing global demand for energy-efficient and sustainable systems has driven significant advancements in power electronics, particularly in DC-DC conversion technologies ^1^. Traditional Boost converters, while effective in many applications, often have difficulty to achieve the high-voltage gains required in modern power systems, such as photovoltaic solar systems ^2^, electrical vehicles (EV) ^3^, High-Voltage Direct Current (HVDC) power transmission systems ^4^, water pumping systems ^5^, or others. Addressing the limitations of conventional topologies, this work introduces a novel interleaved quadratic DC-DC Boost converter designed to provide significantly higher voltage gain without sacrificing efficiency.

Over the years, numerous DC-DC Boost converter topologies have been developed for different applications in a wide range of emergent multidisciplinary engineering fields, such as renewable energy sources (RES), photovoltaic solar energy conversion, EV, energy storage systems (ESS), fuel cells, among others.

Typically, DC-DC converters can be classified according to different features, such as isolated ^6, 7^ or non-isolated ^8, 9^, unidirectional ^10, 11^ or bidirectional ^12, 13^, voltage-fed ^14, 15^ or current-fed ^16, 17^, hard-switch ^18, 19^ or soft-switch ^20, 21^, minimum-phase ^22^ or non-minimum-phase ^23^. Most of these DC-DC converters are well represented in 24– 27. Another way to classify the DC-DC converters is specifying their voltage Boost technique. Some of the most well-known techniques are the switched capacitors ^28, 29^, voltage multiplier cells ^30, 31^, switched inductors ^32, 33^, voltage lift ^34, 35^ and multi-stage/-level ^36, 37^ topologies. A review of some of these step-up voltage techniques can be found in 38– 40. Nowadays, engineering research is also focused on the development of converters with higher reliability, higher efficiency, combined with less volume, weight and cost ^41^.

Among the DC-DC converter topologies developed recently that have stood out for the high-voltage gains obtained are those that present quadratic gains. In this way, is possible to highlight some significantly important topologies developed with such features. The solution proposed in 42 is a transformerless high step-up DC-DC converter with a quadratic voltage gain. In this converter, using a duty cycle greater than 0.309 is possible to achieve a higher voltage gain than the classic Boost converter. This solution includes three switches, five diodes, two inductors and three output capacitors. Despite its interest, this solution requires too many components when compared with other solutions. Other quadratic gain topology can be found in 43, where the authors propose a modified classic DC-DC buck-boost converter. Since this topology allows a buck-boost operation, it is only necessary to control one power switch for each operation mode and the additional power switches remain always ON or always OFF. By controlling only one power switch, they developed a setup capable of achieving a quadruple voltage gain for a duty cycle of 0.5. Another similar topology can be found in 44, which created a quadratic high-gain Boost converter, where it was possible to obtain a gain of two times the input voltage at the output with a duty cycle of 0.50. More recently a new DC-DC Boost converter setup with quadratic gain was proposed ^45^. In this solution using a duty cycle of 0.50 is also possible to achieve a triple output voltage. This solution includes one switch, three diodes, two inductors and two output capacitors. Recently, a new quadratic DC-DC Boost converter topology was proposed in 46, which can achieve a quintuple output voltage with a duty cycle of 0.50. This solution requires only one switch, four diodes, two inductors and three capacitors. The main disadvantage of this solution is that the switch must withstand the maximum output voltage.

It is well-known that high-voltage gain is critical in applications with low input voltages, such as those using a reduced number of solar panel strings or where, due to weather variability sometimes produce reduced voltages, and it required to efficiently convert them into much higher output voltages ^47^. Most quadratic DC-DC Boost converters typically offer voltage gains of three to four times the input voltage, which may not be sufficient for advanced applications. The interleaved quadratic Boost topology proposed in this study aims to overcome these limitations by achieving an extended voltage gains over eight times the input voltage, or six times considering a duty cycle of 0.50, providing a more effective solution to integrate additional RES systems. This converter is also characterized by a simple control technique, continuous input and output current, reduced switching voltage stress over the power devices. The proposed solution takes advantage of the interleaved operation, which allows to use multiple circuits (or phases) to process power in parallel. These circuits are operated with time-shifted (interleaved) switching signals to achieve improved performance compared to a single-phase or single-circuit converter. Also, the interleaved operation avoids the need of large output capacitors. The solution is also able to achieve good efficiency according to some preliminary experimental results.

This paper presents the theoretical framework behind the proposed interleaved quadratic DC-DC Boost topology, complemented by some experimental results to confirm the theoretical results and efficiency. This paper is organized into five main sections. Section I is dedicated to the introduction of this subject and importance of DC-DC converters in most modern applications, followed by a brief state-of-the-art over DC-DC converters with quadratic gain. Section II provides a detailed explanation of all the design procedures and considerations on the prototype of the proposed converter. Section III presents a comparison between the proposed converter and other interleaved quadratic Boost DC-DC converters already proposed and implemented in the literature. Section IV is dedicated to presenting and demonstrating the laboratory setup and validation of the results regarding the operation principle, voltage gain obtained and efficiency. Finally, section V presents some conclusions.

## Methods and theoretical analysis

Our investigation methodology was based on an initial theoretical approach using mathematical equations to describe the operation of the electrical circuit and evaluate the performance compared to other topologies, followed by the validation through some computational simulations using MATLAB/SIMULINK software. Next, the operation of the proposed converter was also confirmed by several experimental tests using a laboratory prototype developed exclusively for these tests. The next subsections are dedicated to show these procedures.

### Power circuit layout of the proposed quadratic Boost DC-DC converter

**Figure 1.**
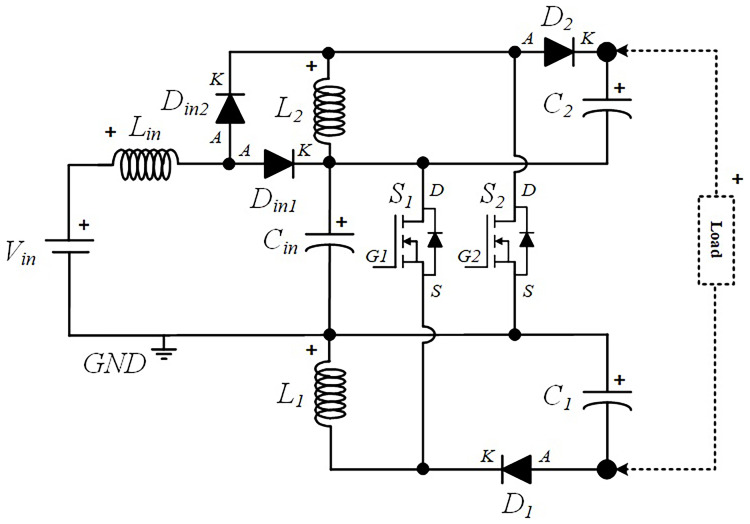
Circuit topology of the proposed interleaved quadratic DC-DC Boost Converter.

Figure 1 shows the diagram of the interleaved quadratic Boost DC-DC converter proposed in this paper. It is a new topology that has never been published before, according to extensive research conducted in the main bibliographic reference resources in the field. The power circuit consists of an input inductor, *L _i_*_*n*_, along with two input diodes, *Din* *1* and *Din* *2*, and a capacitor, *Ci* *n*. Connected to these components are two additional circuits, one at the top and another at the bottom, each consisting of and inductor, *L*_*1*_ and *L*_*2*_, a capacitor, *C*_*1*_ and *C*_*2*_, and a diode, *D*_*1*_ and *D*_*2*_, respectively. Finally, to ensure the capability of voltage regulation and Boost operation, two power MOSFET, *S*_*1*_ and *S*_*2*_, are included, controlled by a command circuit through their gates, represented in the figure as *G*_*1*_ and *G*_*2*_, respectively.

### Operation mode analysis in steady-state

**Figure 2.**
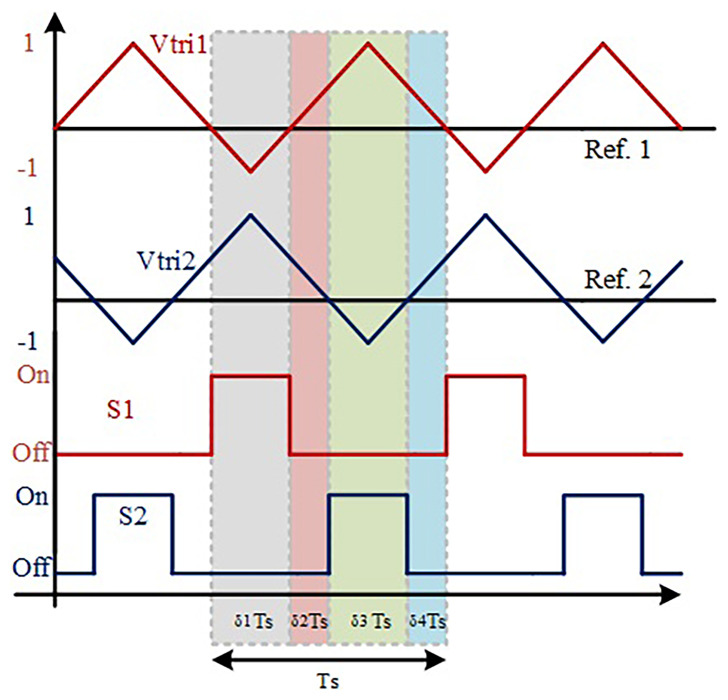
PWM switching strategy of S1 and S2.

The converter under study has four different operation modes, all in continuous conduction mode (CCM), depending on the operation of the two switches, *S*_*1*_ and *S*_*2*_. Although both power semiconductors can operate simultaneously (overlapping the conduction mode) for duty cycles above 0.50, this mode of operation is not advantageous for lower duty cycles, as it generates higher current peaks without resulting in improved voltage gain. Therefore, in the following analysis, it will be considered whether the converter operates with *S*_*1*_ turned ON and *S*_*2*_ turned OFF or *S*_*1*_ turned OFF and *S*_*2*_ turned ON or both switches turned OFF, providing four different operating intervals as explained next. Figure 2 shows a simplified representation of a classic PWM (Pulse-Width-Modulation) control strategy in order to achieve the described operation mode. This is considered an interleaved operation.

In the following figures, the four stationary operation modes are illustrated in detail, where the current flow directions in the different paths are represented with different colours to help understanding the operation principle of the converter.

**Figure 3.**
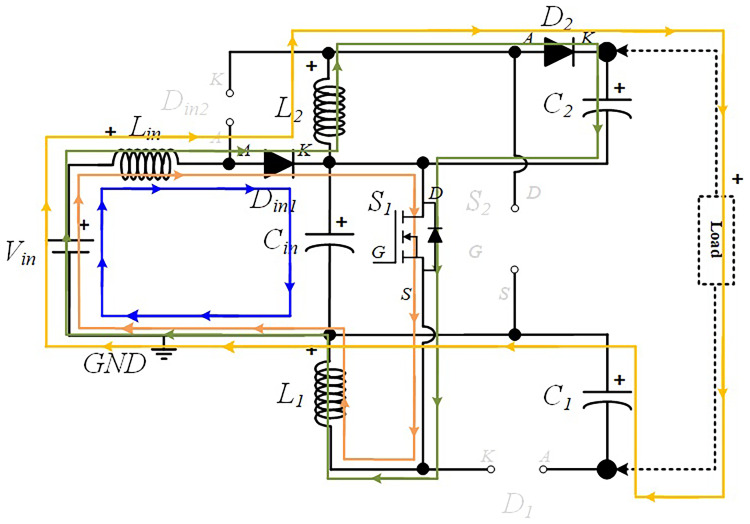
Current flow analysis when S1 turned ON and S2 turned OFF (δ1Ts).

***S1 turned ON and S2 turned OFF ( *δ1Ts*).*** During this operating mode, the input diode *D _in_*_*2*_ is turned OFF, while the input diode *D _in_*_*1*_ is turned on. Also, during this mode, the input inductor *L _i_*_*n*_, discharges the energy accumulated in the previous operating mode over the input capacitor *C _i_*_*n*_ which is in charging mode. Meanwhile, the diode *D*_*1*_ is also turned OFF because the inductor *L*_*1*_ is charging and the capacitor *C*_*1*_ is discharging, creating a reverse voltage over *D*_*1*_. On the other hand, *D*_*2*_ is turned ON, meaning that *L*_*2*_ is discharging the energy previously accumulated, and as a result, *C*_*2*_ is in charging mode. The current flow described is illustrated in Figure 3.

**Figure 4.**
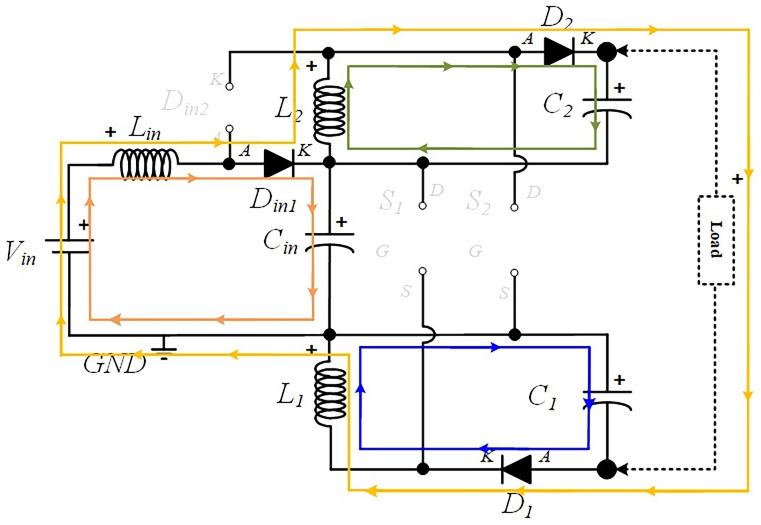
Current flow analysis when S1 and S2 are turned OFF (δ2Ts).

***S1 OFF and S2 OFF ( *δ2Ts*).*** In this operating mode, *D _in_*_*2*_ remains turned off while Din1 remains turned ON. Similar to the previous operating mode, *L _i_*_*n*_ is still discharging and Cin in charging mode. Both *D*_*1*_ and *D*_*2*_ are now turned ON since both inductors, *L*_*1*_ and *L*_*2*_, are discharging the accumulated energy over *C*_*1*_ and *C*_*2*_, respectively. The current paths described can be found in Figure 4.

**Figure 5.**
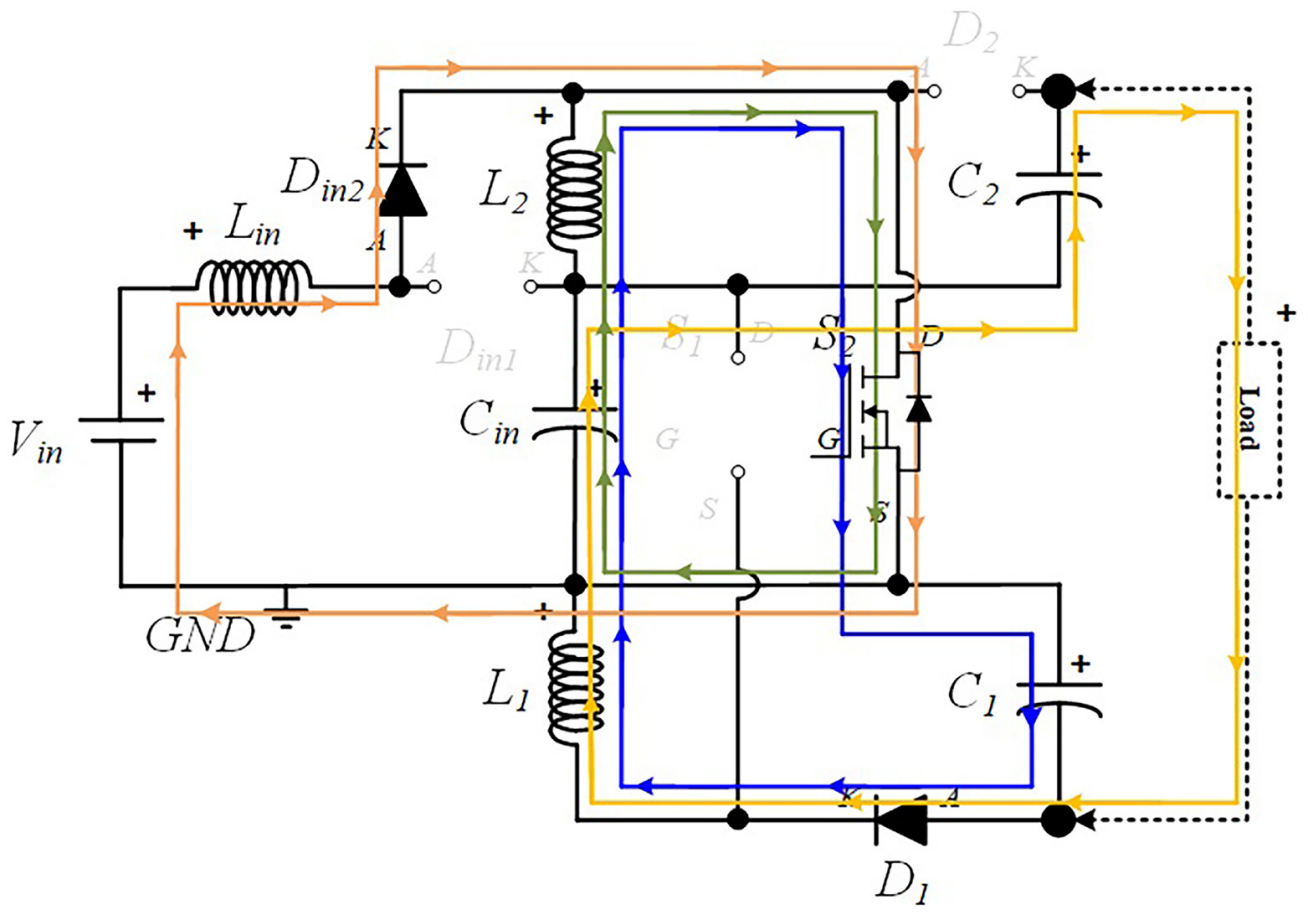
Current flow analysis when S1 is turned OFF and S2 is turned ON (δ3Ts).

***S1 OFF and S2 ON ( *δ3Ts*).*** In this operating mode, after turning ON the switch *S*_*2*_, *D _in_*_*2*_ turns ON to flow the current over the input inductor *L _i_*_*n*_, while *D _in_*_*1*_ turns OFF due to reverse voltage. Thus, *L _i_*_*n*_ is charging, and *C _i_*_*n*_ is discharging the accumulated energy in the previous operating mode over the inductor *L*_*2*_, which is storing energy. As a consequence of passive components polarity, *D*_*2*_ becomes reverse-biased and turned off, while *C*_*2*_ starts to discharge over the load. In the opposite direction, *D*_*1*_ is forced to turn ON to discharging the energy accumulated over the inductor *L*_*1*_ into *C*_*1*_, which is in charging mode. Figure 5 shows the representation of the current path flow described now.

***S1 OFF and S2 OFF ( *δ4Ts*).*** In this operating mode, both switches are turned off and the current’s path flow is the same as the ones presented in the interval *δ2Ts*.

### Component stress analysis

**Figure 6.**
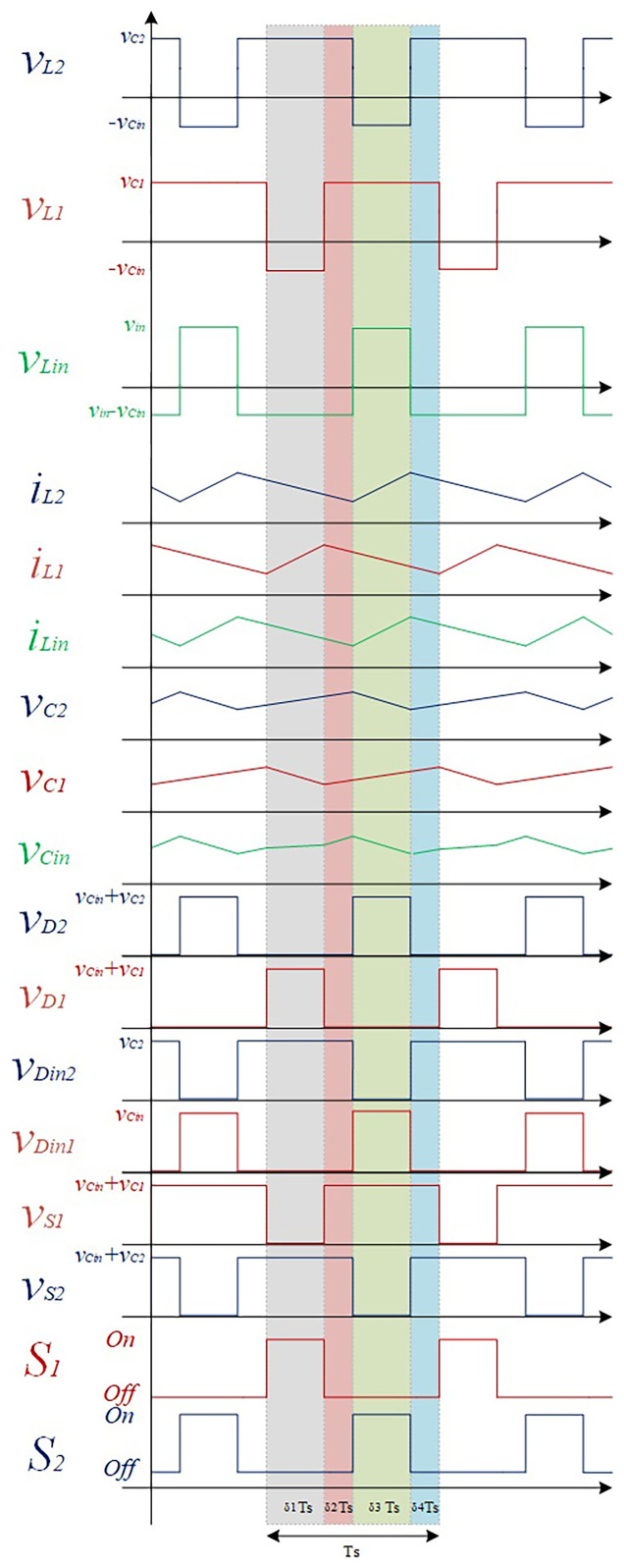
Theoretical wave forms of the proposed DC-DC Converter.

Following the operation modes described previously, it can be observed and concluded that the current in inductor *L _1_* increases when the switch *S _1_* is turned ON and decreases when *S _1_* is turned OFF. This means that the switching state of *S _1_* does not affect the current in *L _in_* and *L _2_*. On the contrary, the current in inductors *L _in_* and *L _2_* increase when switch *S _2_* is turned ON and decreases when *S _2_* is turned OFF. This means that the switching state of *S _2_* does not affect the current in *L _1_*. This indicates a partially independent operation of the two power switches, when the switching states of *S _1_* and *S _2_* do not overlap. According to the principle of operation detailed in the previous subsection, it is possible to obtain the theoretical waveforms of the four-operating mode of the proposed converter (see Figure 6). When analyzing the evolution of the voltage across each inductor and semiconductor presented in this figure is possible to establish the voltage relationships shown in Table 1.

**Table 1.** Voltage relationship between components.

*Voltage/Time*	*(δ _1_Ts)*	*(δ _3_Ts)*	*(δ _2_ Ts, δ _4_Ts)*
*v _L1_*	*-v _Cin_*	*v _C1_*	*v _C1_*
*v _L2_*	*v _C2_*	*-v _Cin_*	*v _C2_*
*v _Lin_*	*v _in_ - v _Cin_*	*v _in_*	*v _in_ - v _Cin_*
*v _Din1_*	*0*	*v _Cin_*	*0*
*v _Din2_*	*v _C2_*	*0*	*v _C2_*
*v _D1_*	*v _Cin_ + v _C1_*	*0*	*0*
*v _D2_*	*0*	*v _Cin_ + v _C2_*	*0*
*v _S1_*	*0*	*v _Cin_ + v _C1_*	*v _Cin_ + v _C1_*
*v _S2_*	*v _Cin_ + v _C2_*	*0*	*v _Cin_ + v _C2_*

According to Table 1, as result of the analysis of the voltage relationships between components it is possible to see that the maximum voltage stress over power devices *S _1_* and *S _2_* are *v _Cin_ + v _C1_* and *v _Cin_ + v _C2_*, respectively, which is far reduced when compared to most DC-DC converters whose power devices must support the maximum output voltage.

### Theoretical voltage gain analysis

In this way, it is possible to establish the following voltage relationships for each inductor. Assuming ideal components and considering one switching cycle, the relationship between the output and input current, function of the duty cycle, can be obtained through the volt-second relationship of the inductors *L*_*1*_, *L*_*2*_ and *L _i_*_*n*_, as presented from ( 1) to ( 3), respectively:


$$\;\delta_{1}(-v_{Cin})=(\delta_{2}+\delta_{3}+\delta_{4})(v_{C1})\;(1)$$

$$\;\delta_{3}(-v_{Cin})=(\delta_{1}+\delta_{2}+\delta_{4})(v_{C2})\;(2)$$

$$\;\delta_{3}(v_{in})=(\delta_{1}+\delta_{2}+\delta_{4})(v_{in}-v_{Cin})\;(3)$$

Knowing that *(δ2 + δ3 + δ4) = (1 – δ1)* and *(δ1 + δ3 + δ4) = (1 – δ3)*, as well as *δ1 = δ3 = δ*; equalizing and solving the Equation (1) to Equation (3) to each capacitor voltage, it is possible to establish the voltage equations listed below from ( 4) to ( 6):


$$\;v_{C1}=\frac{\delta }{1-\delta }v_{Cin}\;(4)$$

$$\;v_{C2}=\frac{\delta }{1-\delta }v_{Cin}\;(5)$$

$$\;v_{Cin}=\frac{1}{1-\delta }v_{in}\;(6)$$

Equalizing and solving Equation (4) to Equation (6) in order to *v _in_*, and knowing that *v _out_ = v _Cin_ + v _C1_ + v _C2_*, it is possible to establish the expression that characterizes the voltage gain of the proposed interleaved quadratic DC-DC converter ( 7):


$$\;v_{out}=\frac{1+\delta }{{\left(1-\delta \right)}^{2}}v_{in}\;(7)$$

### Design considerations

In this section, the entire design process of the passive components used in the proposed experimental prototype will be presented and discussed. The following characteristics were considered in the design of the prototype: *v _in_* = 50 V, *δ _max_* = 0.5, *R _Load_* = 450 Ω, *P _out(max)_* = 200 W, *v _out_* ( *δ _max_*) = 300 V, *i _out_* ( *δ _max_*) = 300/450 = 0.67 A, *Δi _Lmax_* = 0.5 A, *Δv _Cmax_* = 1 V to 3 V, *f _PWM_* = 50 kHz, efficiency of 95%.

### Inductors design

For the inductors design, the generic adopted expression to define the minimum inductance value is presented in ( 8). This expression is based on the linear variation of the current in the inductor and is well explained in most design chapters about DC-DC converters, such as 48– 50.


$$\;L>\frac{v_{L_{max}}\cdot \delta }{f_{PWM}\cdot \Delta i_{L}}\;(8)$$

Where *v _Lma_*_*x*_ is the maximum voltage applied to the inductor, *δ* is the maximum duty cycle intended for the converter, *f _PWM_* is the switching frequency of the converter, and *Δi*_*L*_ is the maximum current variation (ripple) desired in the inductor. For the input inductor, *L _i_*_*n*_, the following equation can be used:


$$L_{in}>\frac{v_{in}\cdot \delta }{f_{PWM}\cdot \Delta i_{L}}\Rightarrow L_{in}>1mH\;(9)$$

For the remaining inductors, *L _1_* and *L*_*2*_, the following equation can be used:


$$L_{1}=L_{2}>\frac{v_{Cin}\cdot \delta }{f_{PWM}\cdot \Delta i_{L}}\Rightarrow \frac{\frac{v_{in}}{\left(1-\delta \right)}\cdot \delta }{f_{PWM}\cdot \Delta i_{L}}>2mH\;(10)$$

### Ferromagnetic material saturation analysis

The material of the inductors, applied in this prototype, uses a Litz 420x0.08 SE F155 G1 wire type (widely used in high frequency applications, as it reduces losses and the skin effect), a plastic inductor winding support from the CF model -E70-1S and a set of ferrite cores from model E70/33/32DG in “U” shape, from the manufacturer TDK, with type N87 ferrite. Using the manufacturer datasheet, it is possible to obtain some essential parameters (see Table 2) for analyzing the electromagnetic saturation of the ferrite core.

**Table 2.** Magnetic Parameters of the Ferrite Core E70/33/32DG.

Magnetic Parameter	*Value*
Effective magnetic cross section (Ae)	683 mm ^2^
Inductance factor (AL)	250 nH
Effective magnetic length (le)	149 mm
Saturation flow density (B)	390 mT

Thus, to calculate the maximum current value that can cross each inductor, before saturating the ferromagnetic material is possible to estimate the number of turns of each winding, taking into account the desired inductance value, *L*, (H) and the inductance factor, *A*_*L*_, (H) of the material adopted ^48– 51^.


$$\;L=N^{2}A_{L}\Leftrightarrow N=\sqrt{\frac{L}{A_{L}}}\;(11)$$

After applying Equation (11) to each inductor, the values expressed below were obtained from ( 12) and ( 13).


$$N_{L_{1}}=N_{L_{2}}=\sqrt{\frac{2\times 10^{-3}}{500\times 10^{-9}}\;}\approx 63turns\;(12)$$

$$\;N_{Lin}=\sqrt{\frac{1\times 10^{-3}}{500\times 10^{-9}}\;}\approx 45turns\;(13)$$

Then, the permeability of the ferrite adopted, μ, (H/m) can be calculated from Equation (14).


$$A_{L}=\frac{\mu \cdot A_{e}}{l_{e}}\Leftrightarrow \mu =\frac{A_{L}\cdot l_{e}}{A_{e}}=\frac{250\times 10^{-9}\cdot 149\times 10^{-3}}{683\times 10^{-6}}=5,4539\times 10^{-5}H/m\;(14)$$

Finally, using Equation (15) is possible to calculate the maximum current over each inductor prior to saturate the ferrite magnetic material.


$$\;I_{máx}=\frac{B_{máx}\cdot l_{e}}{\mu \cdot N}\;(15)$$

Applying ( 15) for all the inductors, the current values can be calculated.


$$I_{L_{1Máx}}=I_{L2_{Máx}}=\frac{390\times 10^{-3}\cdot 149\times 10^{-3}}{5,4539\times 10^{-5}\cdot 63}=16,91A\;(16)$$

$$I_{L_{in_{Máx}}}=\frac{390\times 10^{-3}\cdot 149\times 10^{-3}}{5,4539\times 10^{-5}\cdot 45}=23,67A\;(17)$$

### Capacitors design

Similarly, for the capacitors design, the generic adopted expression to define the minimum capacitance value is presented in ( 18). This expression is based on the linear variation of the voltage in the capacitors.


$$\;C>\frac{i_{C_{max}}\cdot \delta }{\Delta v_{C}\cdot f_{PWM}}\;(18)$$

Where *i _Cma_*_*x*_ is, generally, the maximum current flowing through the capacitor, *δ* is the maximum duty cycle intended for the converter, *f _PWM_* is the switching frequency of the converter, and *Δv*_*C*_ is the maximum voltage variation (ripple) desired in the capacitor. For the input capacitor is necessary to obtain the input current, which can be obtained based on the output power, efficiency and input voltage ( 19). It was selected a maximum ripple *Δv _Cma_*_*x*_ = 3 V for the input capacitor and *Δv _Cma_*_*x*_ = 1 V for the remaining capacitors.


$$C_{in}>\frac{(i_{out}-(i_{L_{1}}+i_{L_{2}}))\cdot \delta }{\Delta v_{C}\cdot f_{PWM}}\Rightarrow C_{in}>\frac{\frac{P_{in}}{v_{in}}\cdot \delta }{\Delta v_{C}\cdot f_{PWM}}\Rightarrow C_{in}>14\mu F\;(19)$$

For the capacitors *C _1_* and *C _2_*, the following Equation (20) can be used.


$$\;C_{1}=C_{2}>\frac{i_{out}\cdot \delta }{\Delta v_{C}\cdot f_{PWM}}\Rightarrow C_{1}=C_{2}>6.7\;\mu F\;(20)$$

These calculations shows that the proposed interleaved converter requires small capacitor which is an advantage over other topologies.

### Power circuit PCB design

A printed circuit board (PCB) allows the integration of multiple passive and active electronic components in an electrically and structurally efficient manner. However, designing PCBs for power electronics requires compliance with several design constraints, such as adequate clearance between traces to prevent electrical arcing and sufficiently wide copper tracks to avoid overheating and excessive resistive losses.

The PCB design followed the guidelines of the IPC-2221 *“Generic Standard on Printed Board Design* *”*, a widely recognized reference also adopted by KiCad ^®^, the software used for this article. KiCad ^®^ was selected for its intuitive interface, reliability, and suitability for rapid prototyping. The PCB was structured into three main functional sections: power circuit, control circuit, and measurement circuit. This modular layout improves clarity for assembly, troubleshooting, and testing. The first step in the PCB design was to define the trace widths based on the maximum expected current, according to Section 6.2 ( *Conductive Material Requirements*) of IPC-2221, expressed by:


$$\;I=k\Delta {Τ}^{0.44}{\left(W\cdot H\right)}^{0.725}\;(21)$$

where *I* is the maximum current (A), *ΔT* is the temperature rise above ambient (°C), *W* is the trace width (mm), *H* is the trace thickness (mm), and *k = 0.048* for external layers.

Assuming a 10 °C temperature rise and a 35 μm copper thickness, the resulting maximum current capacities are approximately 6.53 A for 4 mm traces (power stage), 2.39 A for 1 mm traces, and 1.45 A for 0.5 mm traces (control stage).

During layout design, several IPC-2221 best practices were also applied:

- 45° routing angles were used to minimize electromagnetic interference (Section 10.3.1, *Conductor Routing*).
- Minimum spacing between traces was set to 0.4 mm for 100–300 V and 0.8 mm for voltages above 300 V, according to Section 6.3 ( *Electrical Clearance*) and Table 6.1 ( *Electrical Conductor Spacing*).
- Decoupling capacitors were added near power devices to minimize voltage ripple (Section 6.4.6, *Inductance Considerations*).
- A large ground plane was implemented to reduce electromagnetic noise and improve circuit stability (Sections 6.4.6 and 10.1.2).

**Figure 7.**
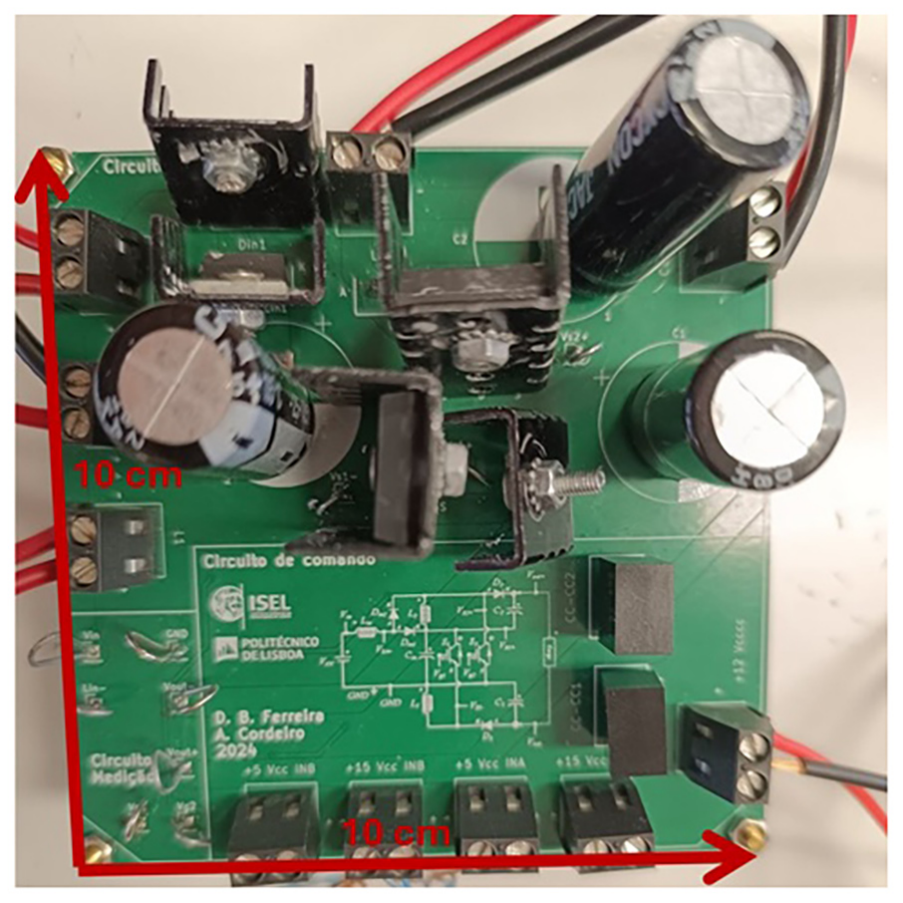
Power circuit PCB (Printed Circuit Board) photo.

Figure 7 shows the fabricated PCB, with all components soldered with tin on the bottom layer of the board.

### Gate driver circuit PCB design

A pulse-width modulation (PWM) strategy was implemented to operate the proposed converter. The open-loop control setup was designed to evaluate the converter’s performance in terms of voltage gain and efficiency. This configuration also serves as a basis for later integration into a closed-loop system with stability analysis. Next sections present PLECS simulations of the converter’s dynamic response using PI output voltage control and MPPT-based PV input regulation.

**Figure 8.**
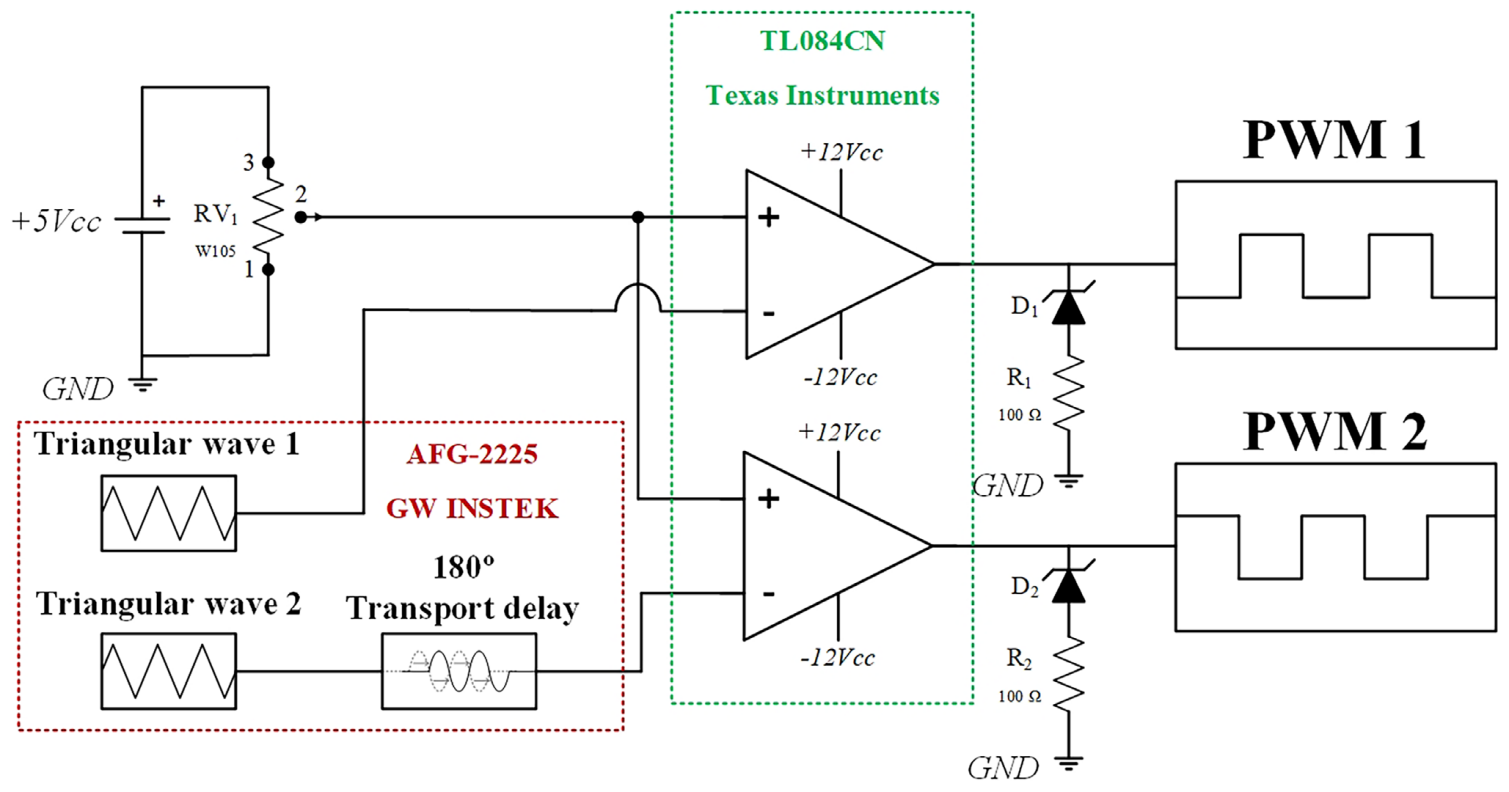
Open-loop PWM circuit diagram.

As shown in Figure 8, the control circuit uses a variable resistor (RV1, 1 MΩ) to adjust the reference command voltage applied to the non-inverting terminals of the operational amplifiers. Two triangular carrier signals (0° and 180° phase-shifted, 0–5 V range) were generated using a GWINSTEK AFG-2225 signal generator to create the interleaved PWM signals.

The comparator stage employs TL084CN operational amplifiers (Texas Instruments), powered from ±7 V. To shift the output range from ±7 V to 0–7 V, each output includes a Zener diode in series with a 100 Ω resistor, clamping the negative voltage near zero.

**Figure 9.**
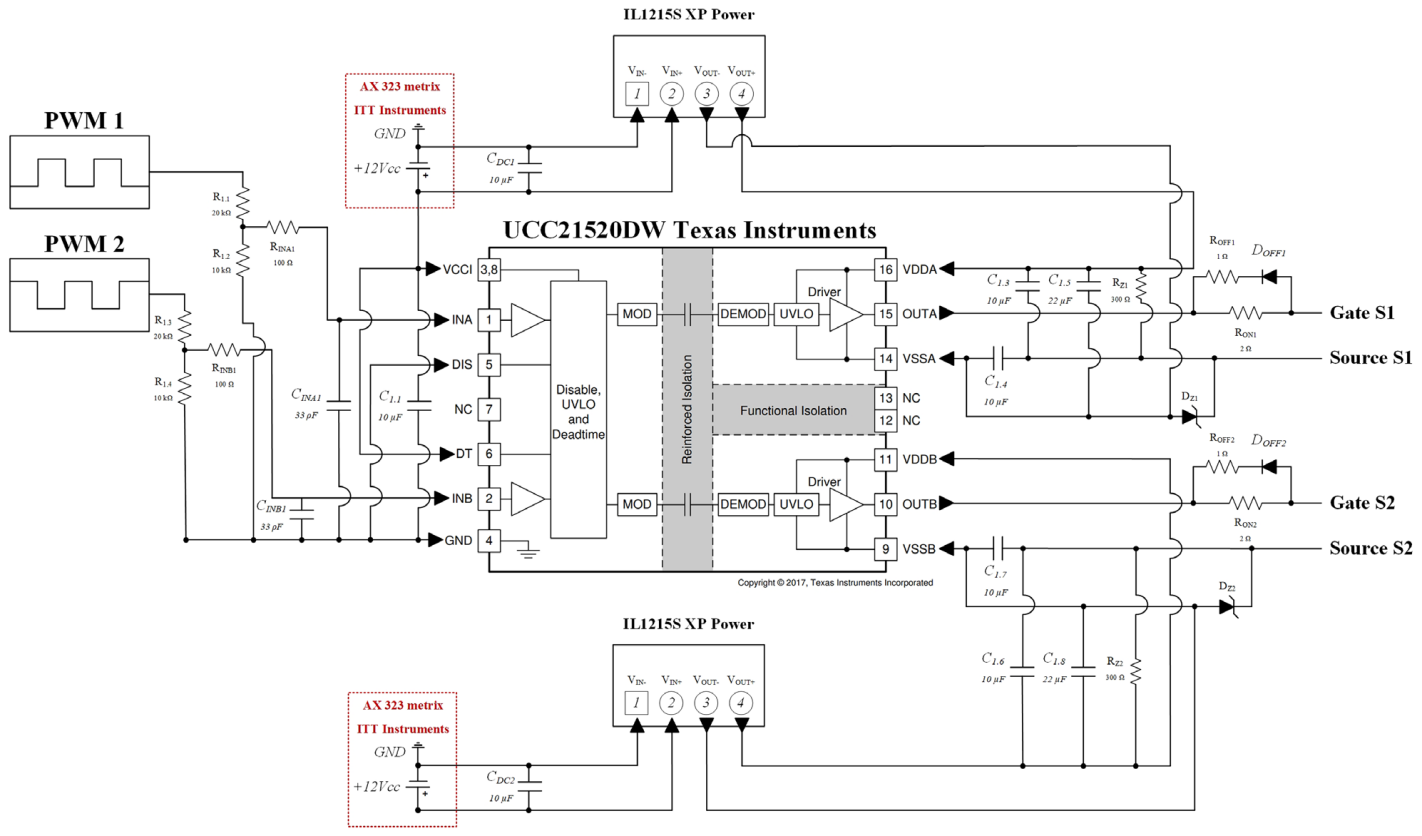
Gate driver circuit diagram used in the PCB (components soldered with tin on the bottom layer).

The gate driver circuit provides galvanic isolation and signal amplification between the control and power stages, as illustrated in Figure 9. It is based on the UCC21520DW isolated dual-channel gate driver (Texas Instruments), which offers up to 5.7 kV isolation, 100 V/ns common-mode transient immunity, 6 A peak drive current, and a 19 ns propagation delay. The internal capacitive isolation technology uses a high-voltage dielectric layer to block DC current while maintaining signal integrity between high- and low-voltage domains.

The driver’s primary supply (VCCI) was provided by an external AX 323 Metrix DC source set to +12 V, while isolated bias voltages for each channel were generated using IL1215S isolated DC-DC converters (XP Power). 10 µF ceramic decoupling capacitors (CDC1, CDC2) were added at each converter input to suppress noise and stabilize supply voltages.

At the PWM input pins (INA, INB), 100 Ω resistors and 33 pF ceramic capacitors form RC filters to suppress high-frequency interference and improve noise immunity, following manufacturer recommendations. Additional bypass capacitors (10 µF and 22 µF) were distributed across the circuit to enhance stability. The DT pin (Pin 6) was tied to VCCI (Pin 3) to allow overlapping outputs, and the DIS pin (Pin 5) was grounded for improved noise immunity.

At the driver outputs (OUTA, OUTB), turn-on (RON = 2 Ω) and turn-off (ROFF = 1 Ω) resistors were added to reduce oscillations from parasitic inductances and minimize switching losses. Additional Zener diodes (DZ1, DZ2, –5.1 V) and 300 Ω resistors (RZ1, RZ2) were placed between the gate and source of each MOSFET to prevent unintended turn-on due to voltage overshoot ( *dv/dt*) or parasitic coupling. This configuration ensures stable operation and protects the power devices from false triggering.

### Comparison with other interleaved quadratic DC-DC Boost topologies

This section is intended to compare the proposed solution with other interleaved quadratic Boost DC-DC topologies presented in the references ^52– 55^. Table 3 shows a comparison between the number of components needed to achieve the voltage gain obtained by each converter and maximum voltage stress over the power devices.

**Table 3.** Comparison between some different interleaved quadratic Boost DC-DC topologies.

	*Topologies*					
	*Proposed*	*52*	*53*	*54*	*55*	*56*
*Number of Switches*	*2*	*1*	*2*	*2*	*2*	*4*
*Number of Diodes*	*4*	*3*	*6*	*4*	*6*	*2*
*Number of Inductors*	*3*	*2*	*4*	*4*	*4*	*4*
*Number of Capacitors*	*3*	*2*	*4*	*3*	*3*	*2*
*Switching Frequency*	*50 kHz*	*20 kHz*	*40 kHz*	*50 kHz*	*20 kHz*	*50 kHz*
Voltage gain *	$\frac{1+\delta }{{\left(1-\delta \right)}^{2}}$	$\frac{1}{{\left(1-\delta \right)}^{2}}$	$\frac{2}{{\left(1-\delta \right)}^{2}}$	$\frac{\left(1+n)(2-d\right)}{{\left(1-\delta \right)}^{2}}$	$\frac{1}{{\left(1-\delta \right)}^{2}}$	$\frac{2n+2}{{\left(1-\delta \right)}^{2}}$
*Maximum Voltage stress over switches*	$\frac{V_{out}-\delta }{{\left(1-\delta \right)}^{2}}V_{in}$	*V _out_*	$\frac{V_{out}}{2}$	$\frac{V_{in}}{{\left(1-\delta \right)}^{2}}$	*V _out_*	$\frac{V_{out}}{2(n+1)}$

* n-winding ratio between coupled inductors

The comparison between these topologies shows that the solution proposed in this article is not the one that achieves the highest voltage gain but presents a relatively high voltage gain with less components and is one that presents the most reduced voltage stress over the power devices.

**Figure 10.**
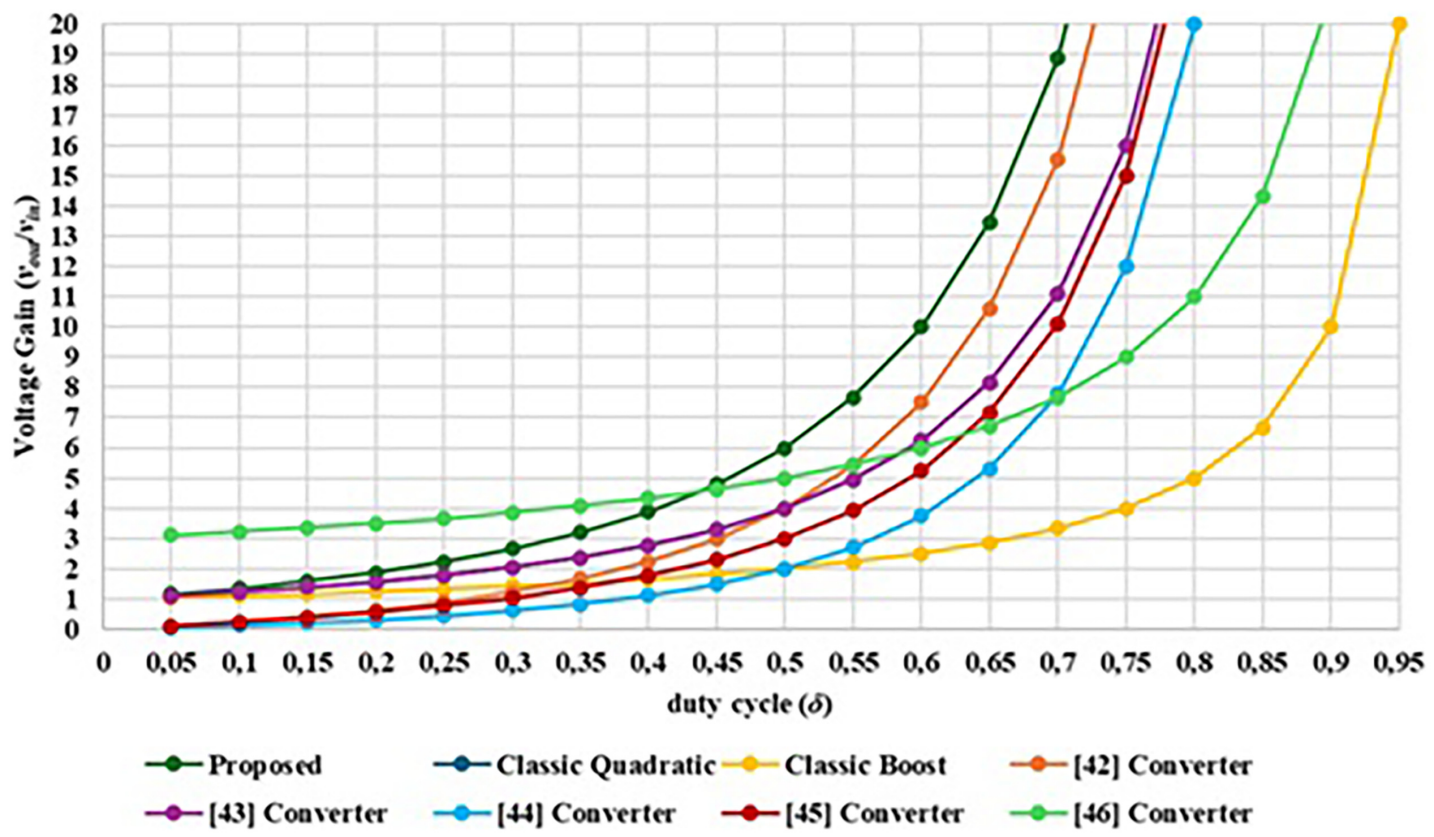
Comparison between the proposed interleaved quadratic DC-DC converter other similar topologies regarding the voltage gain versus the duty cycle.

Figure 10 compares the theoretical voltage gain characteristics of each DC-DC Boost converter topology. As shown in Figure 10, the proposed converter is able to provide a voltage gain output of six times the input voltage for δ = 0.5. The solution presented in 56 presents the best voltage gain below 0.45 but the voltage gain rate above 0.45 is smaller than the proposed topology.

### Laboratory validation

**Figure 11.**
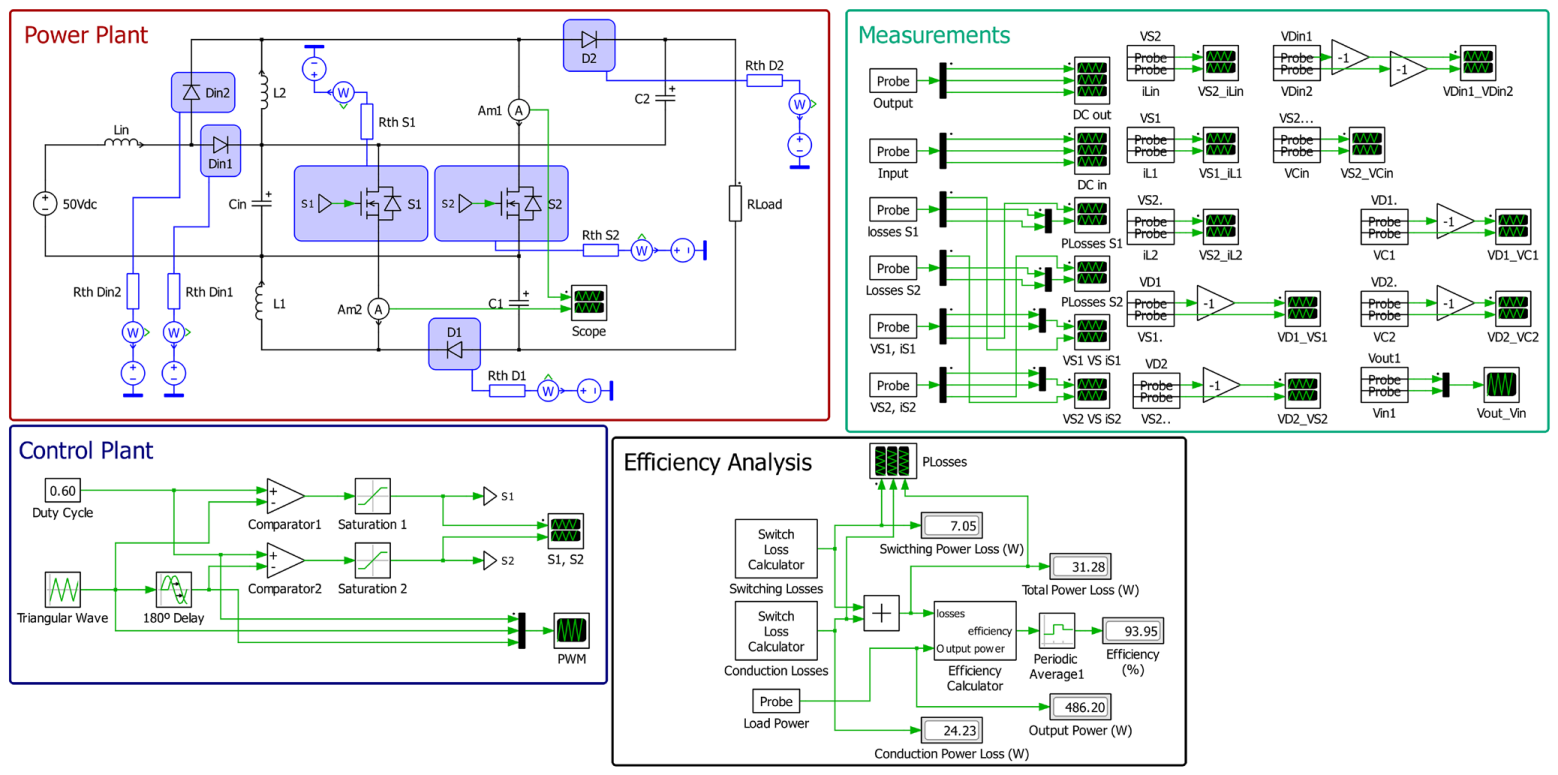
PLECS ^®^ schematic of the proposed DC/DC converter when applying an open-loop PWM control.

***Open-loop PWM Control (Simulation test setup).*** Figure 11 presents the schematic used in PLECS ^®^ to simulate the behavior of the proposed DC-DC converter when applying a simple open-loop PWM control. As seen in the figure, the schematic is divided into four boxes, the red one being the power plant, the blue one being the control plant, the green one dedicated to measurements and the black one to the efficiency analysis. All power devices were simulated using both their electrical and thermal datasheet information into the PLECS ^®^ environment. The efficiency was calculated using the dedicated PLECS ^®^ box available in the software, enabling the correct calculations of both conduction and switching losses.

***Open-loop PWM Control (Experimental test setup).*** The practical tests of the proposed converter prototype were carried out considering a maximum output power of 200 W, using a constant load resistance of 450 Ω, an input voltage of 50 V and a switching frequency ( *f _sw_*) of 50 kHz. The inductors are *L _1_=L _2_=2 mH* and *L _in_=1 mH*. The capacitors are *C _in_=22 μF* and *C _1_=C _2_=10 μF* (normalized values). It should be noted that all practical results were always compared with a computational validation of the converter circuit operation, employing the PLECS ^® ^software. Table 4 summarizes all the components used in the prototype converter.

**Table 4.** Components used in the prototype proposed DC/DC converter.

Component	Part Number (Rating)
Power Switches (S _1_, S _2_)	C2M0080120D (1200 V, 36 A – SiC MOSFET TO-247-3)
Diodes (D _in1_, D _in2_, D _1_, D _2_)	FFSP2065B-F085 (650 VDC, 20 A – TO-220-2L EliteSiC)
Inductor (L _in_)	1 mH (16.91 A)
Inductors (L _1_, L _2_)	2 mH (23.67 A)
Capacitor (C _in_)	22 µF (450 VDC - Electrolytic)
Capacitors (C _1_, C _2_)	10 µF (450 VDC - Electrolytic)

**Figure 12.**
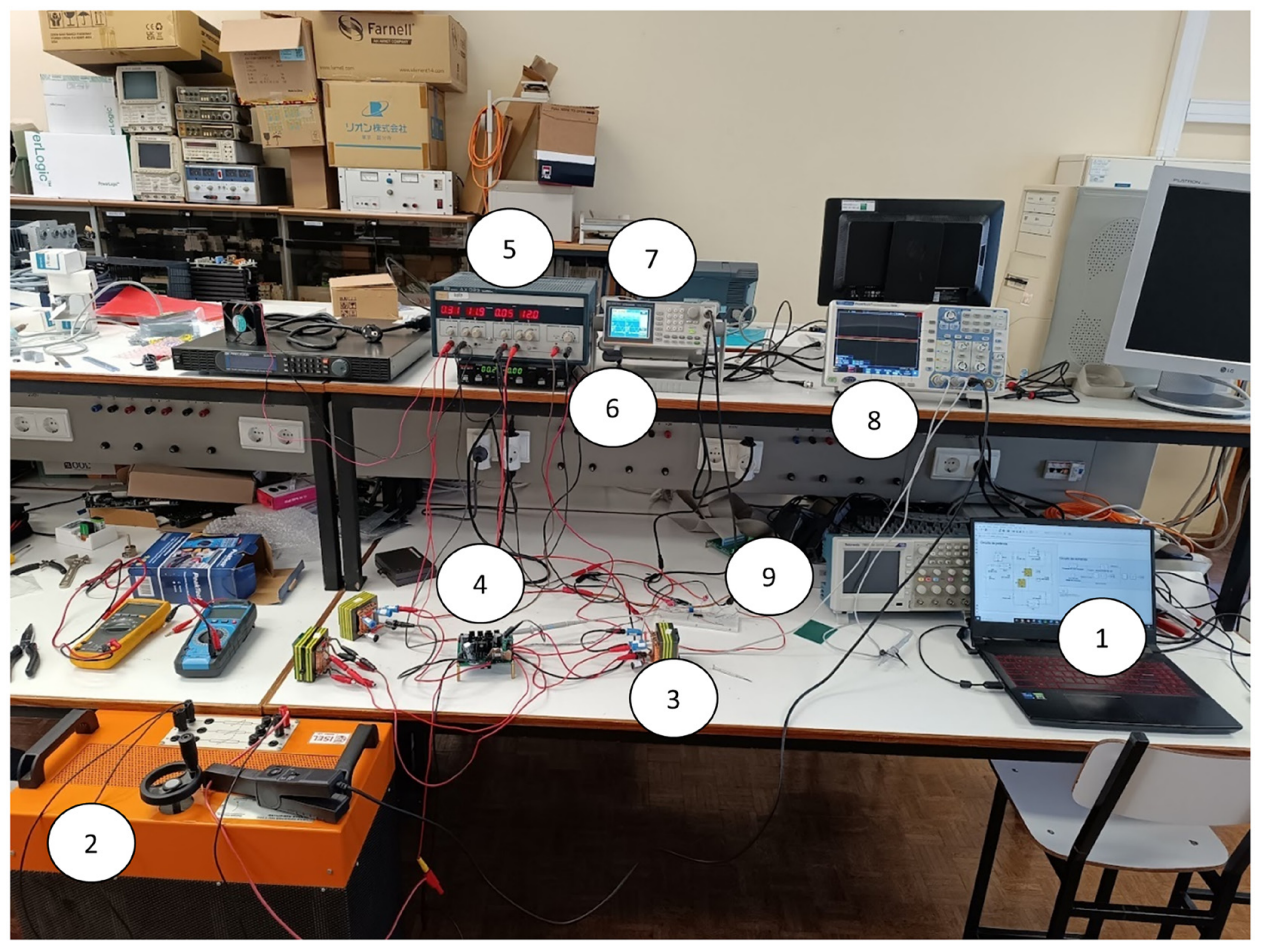
Workbench with the proposed DC-DC converter prototype (1–Laptop with the circuit simulation in PLECS®; 2 – Load resistor; 3 – Inductor; 4 – Printed Circuit Board (PCB) of the power circuit and gate drive circuit; 5 – Power supply for the control circuit; 6 – Power Supply for the power circuit; 7 – Signal generator; 8 – Oscilloscope; 9 – Breadboard with the PWM control circuit).

Figure 12 shows a wide-angle photogrph of the workbench with the proposed converter prototype and all the essential devices to test the solution.

### Inductors and power devices waveforms

**Figure 13.**
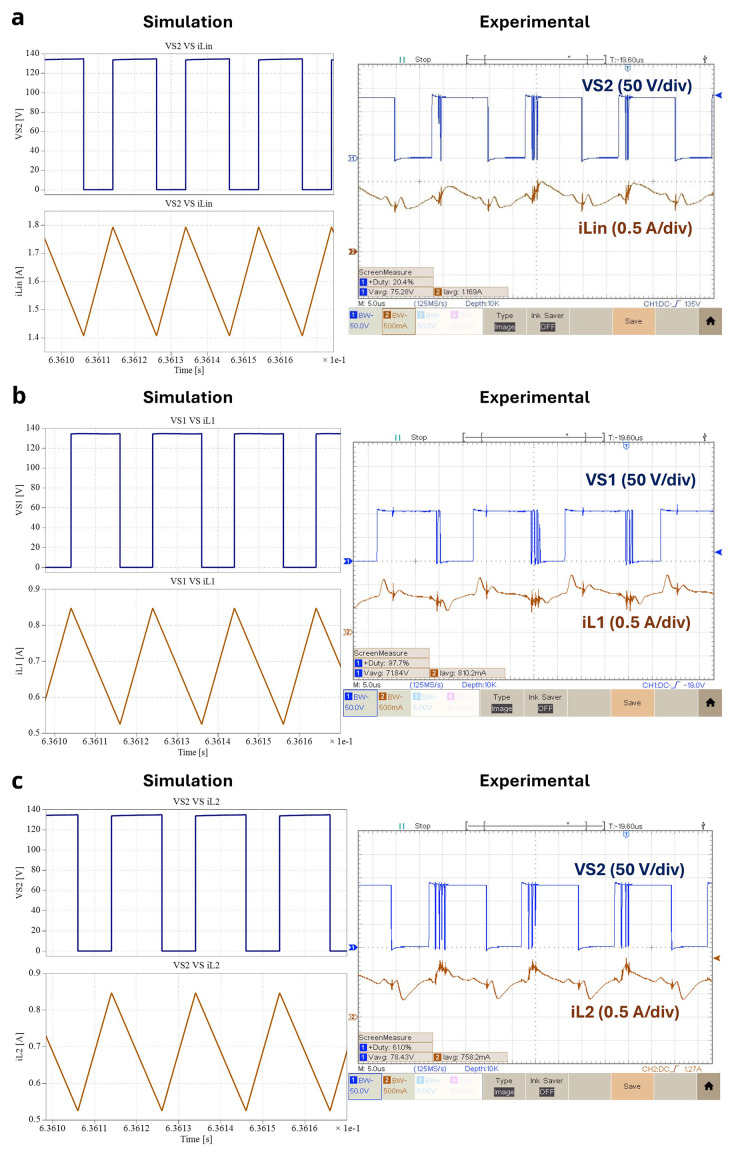
Inductor currents and power devices voltages for δ = 0.4: **a**) iLin and vS2; **b**) iL1 and vS1; **c**) iL2 and vS2.

Figure 13 shows the experimental result of the three inductor currents obtained from the power circuit of the proposed converter for δ = 0.4 and relation with the switching of the power devices. The results presented in Figure 13 confirm the dependence between each inductor and one of the power switches, specifically, the state of charge of *L _in_* and *L _2_* depends on the conduction state of S2, and the same situation occurs between *L _1_* and *S _1_*. The mean current values are: *iL _in_* = 1.169 A, *iL _1_* = 810.2 mA and *iL _2_* = 758.2 mA.

### Diodes and power devices waveforms

**Figure 14.**
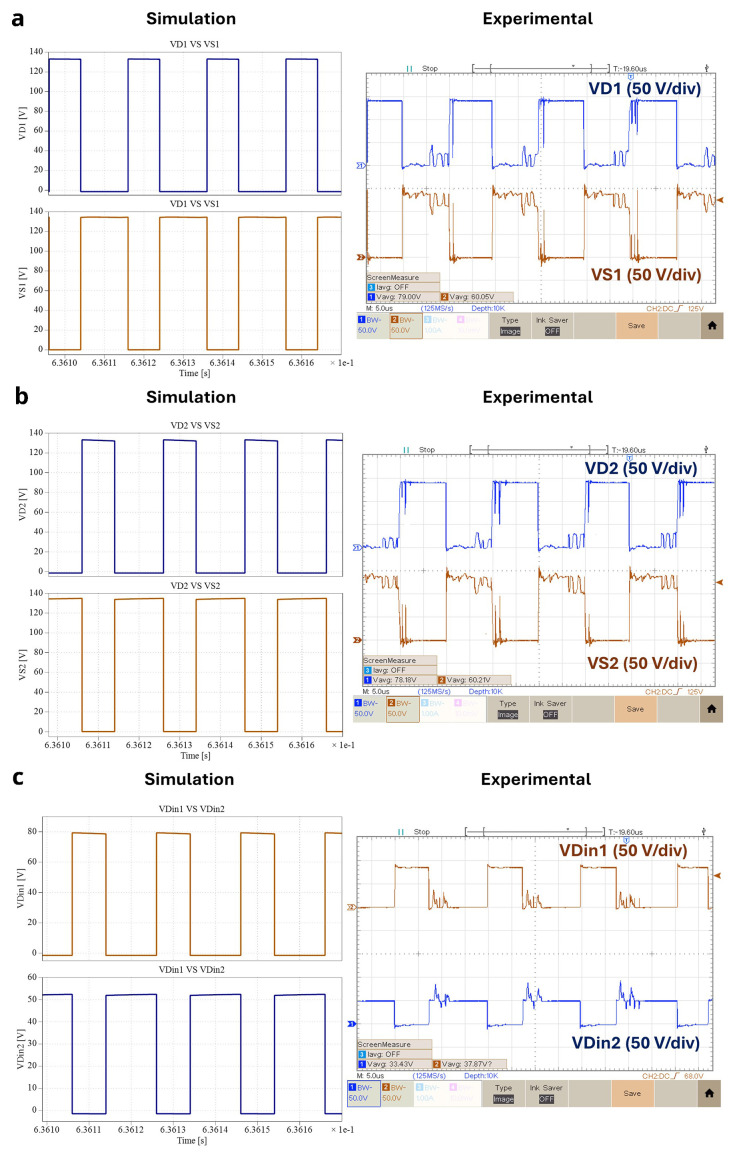
Diodes and power switches voltages for *δ* = 0.4: **a**) vD1 and vS1; **b**) vD2 and vS2; **c**) iLin , vDin1 and vDin2.

Figure 14 illustrates the experimental results of the relationship between the diodes and the power switches voltage for *δ* = 0.4. According to the results shown above ( Figure 13a) and Figure 13b), the conduction states of *D _1_* and *D _2_* are symmetric to the conduction state of *S _1_* and *S _2_*, respectively. On the other hand, the conduction states of both input diodes *D _in1_* and *D _in2_* are dependent on the *S _2_* (see Figure 14c) state, just like *i _Lin_*, as concluded before in Figure 13a). Notice that the power devices voltage waveforms are different depending on the experimental result for the same duty cycle, which is probably due to coupling of common mode noise, which is more intensive as the switching frequency increases.

### Capacitors and power devices waveforms

**Figure 15.**
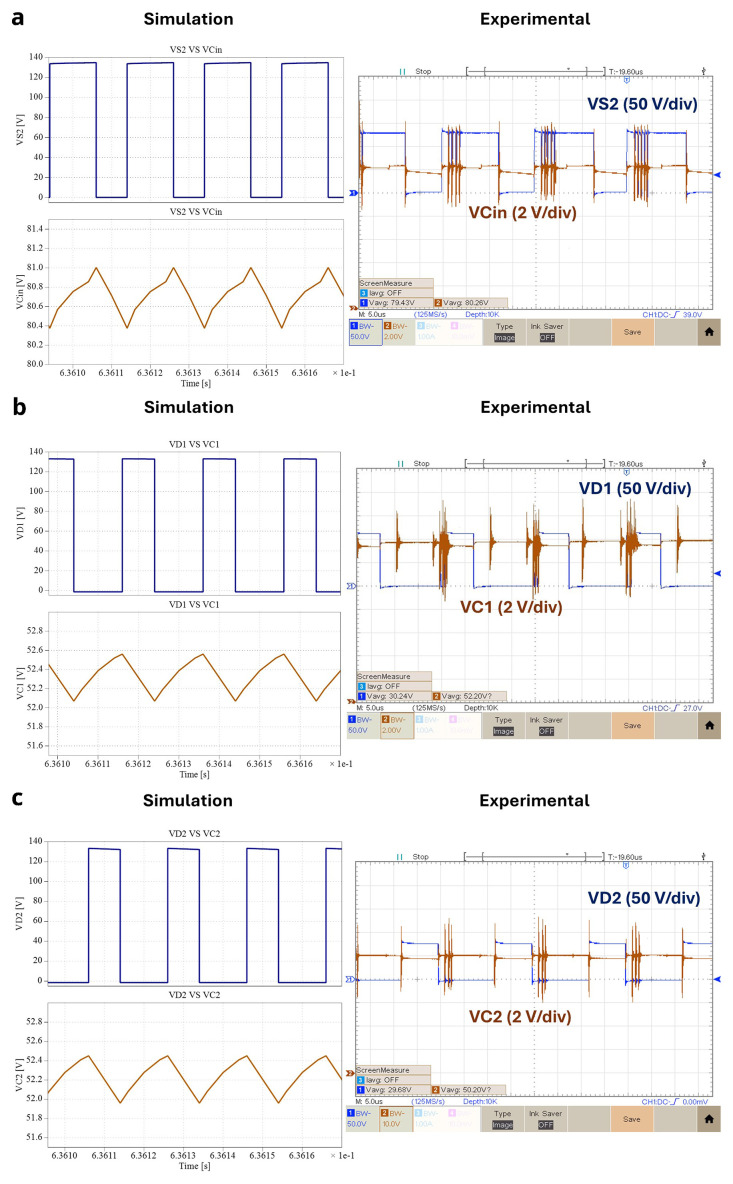
Capacitor and power switches voltages for *δ* = 0.4: **a**) vCin and vS2; **b**) vC1 and vD1; **c**) vC2 and vD2.

Figure 15 shows the experimental results of each capacitor and the power switches voltages for *δ* = 0.4. Interpretating the results shown in Figure 15 and remembering the symmetric relation between *D _1_* and *S*_*1*_, along with *D*_*2*_ and *S*_*2*_, it is clear the correspondence between the state of charge of both *C _i_*_*n*_ and *C*_*2*_ and *S*_*2*_, as well as, between *C*_*1*_ and *S*_*1*_. Making a parallel analysis between the results of Figure 13 and Figure 15, it is also possible to observe when an inductor is discharging the corresponding capacitor is charging and vice-versa. The mean voltage values are: *v _Ci_*_*n*_ = 80.26 V, *v _C_*_*1*_ = 52.20 V and *v _C_*_*2*_ = 50.20 V. Notice that in the following figure, the Ch2 voltage gain is only 2V/div, and the reference is virtually several divisions bellow the minimum visible at the screen (this Peaktech oscilloscope allows this configuration). Thus, the noise is not so high as it seems at first sight.

Concerning the comparison between the simulation and experimental results, it is shown in all the figures presented from Figure 13 to Figure 15 that the simulation behavior is correctly confirmed by the experimental results.

### Output voltage and voltage gain

**Figure 16.**
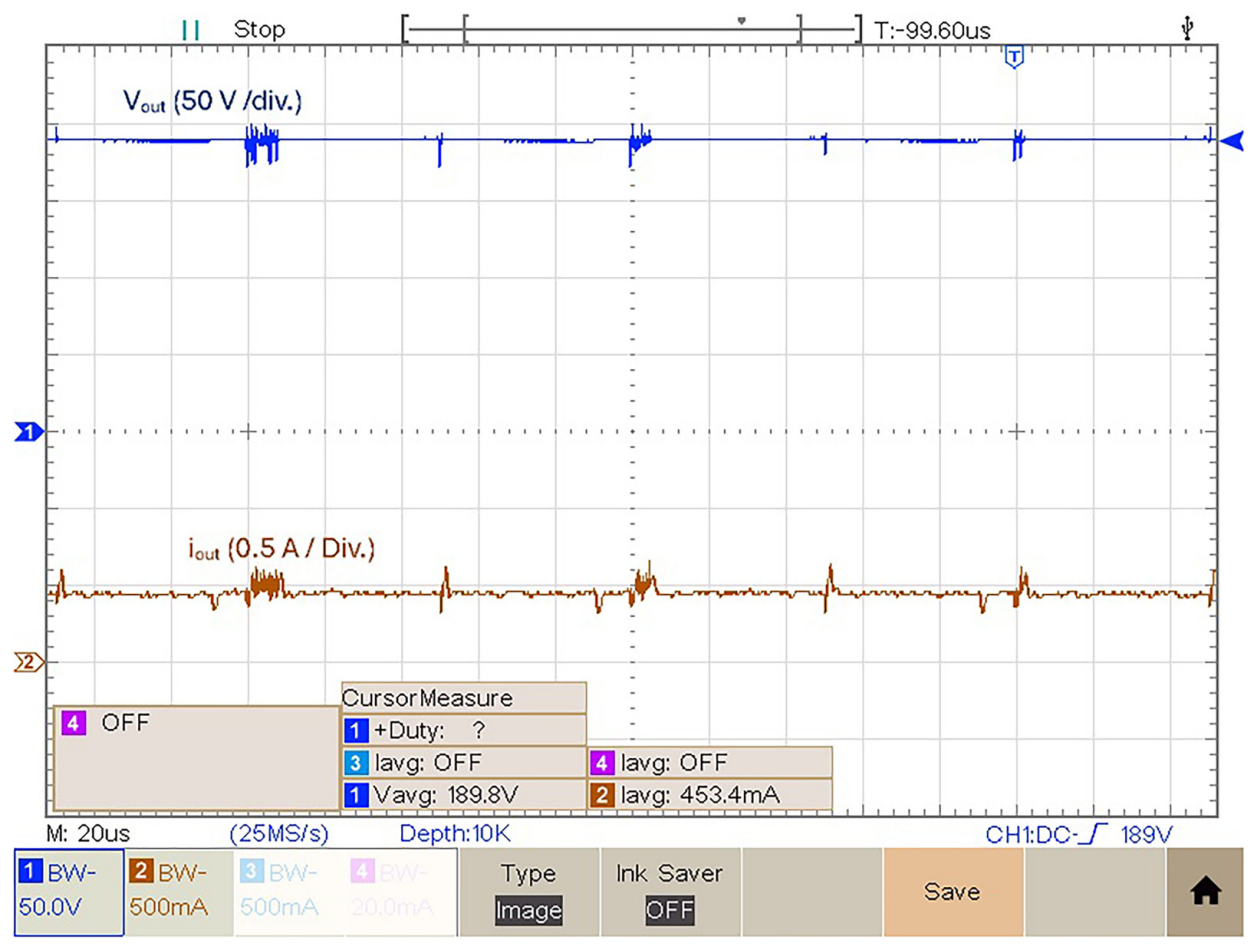
Output voltage and current for a duty cycle, *δ* = 0.4.

In this subsection several experimental results are presented of the output voltage, output current and a voltage gain comparison for different duty cycles. Figure 16 features the experimental voltage and current output result obtained with *δ* = 0.4. Observing Figure 16 it is possible to see that for *δ* = 0.4 the proposed prototype is able to produce an output voltage *v _out_* = 189.8 V, which translates to a voltage gain ( *v _out_/v _in_*) of 3.66. The mean value of the output current is equal to 453.4 mA.

**Figure 17.**
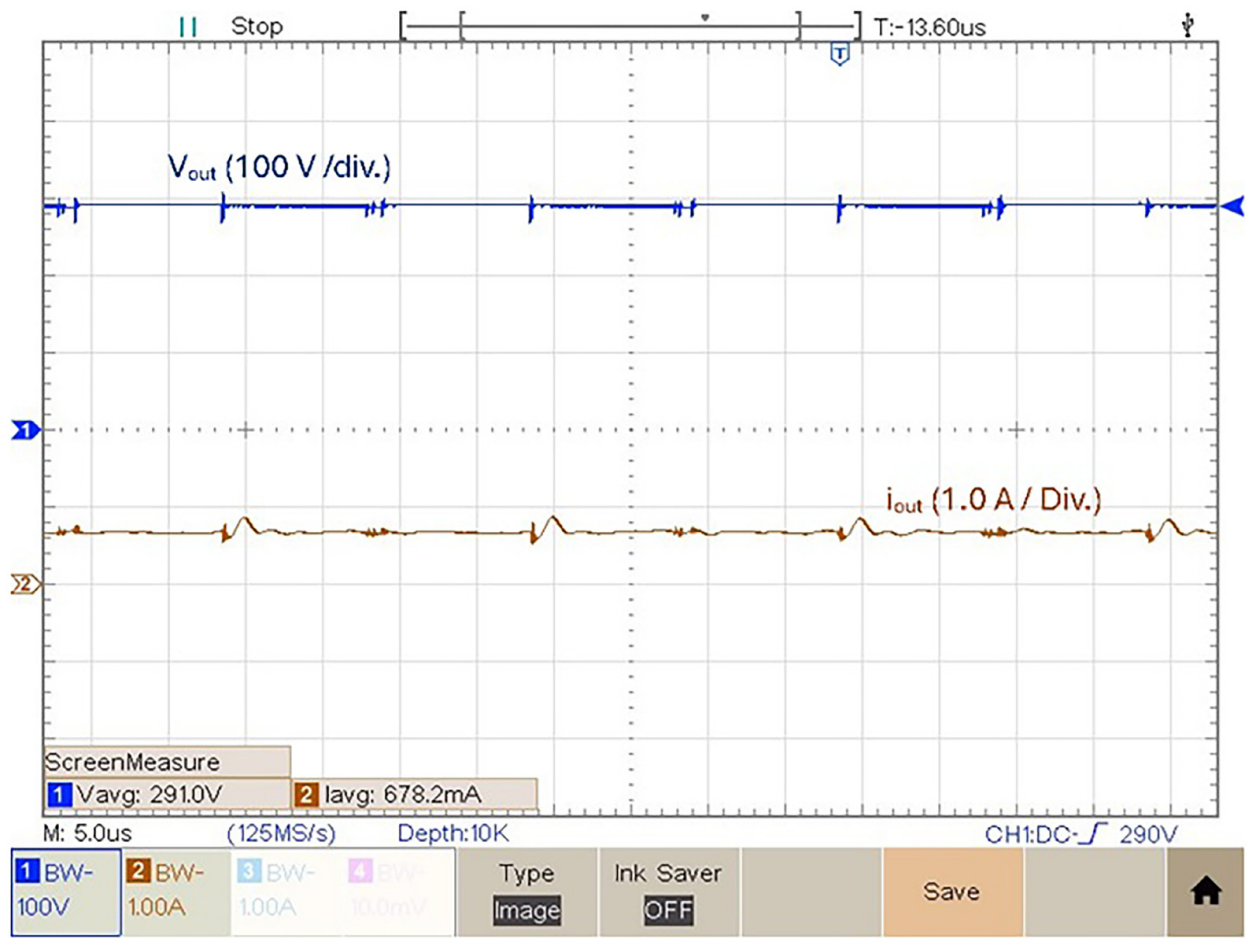
Output voltage and current for a duty cycle, *δ* = 0.5.

Figure 17 shows the experimental result of the output voltage and output current obtained in the prototype for *δ* = 0.5. Observing figure 17 with this duty-cycle is possible to observe an output voltage *v _out_* = 291.00 V, which translates to a voltage gain ( *v _out_/v _in_*) of 5.82. The mean value of the output current is equal to 690.6 mA, which translates into a *P _out_* ≈ 200 W.

**Figure 18.**
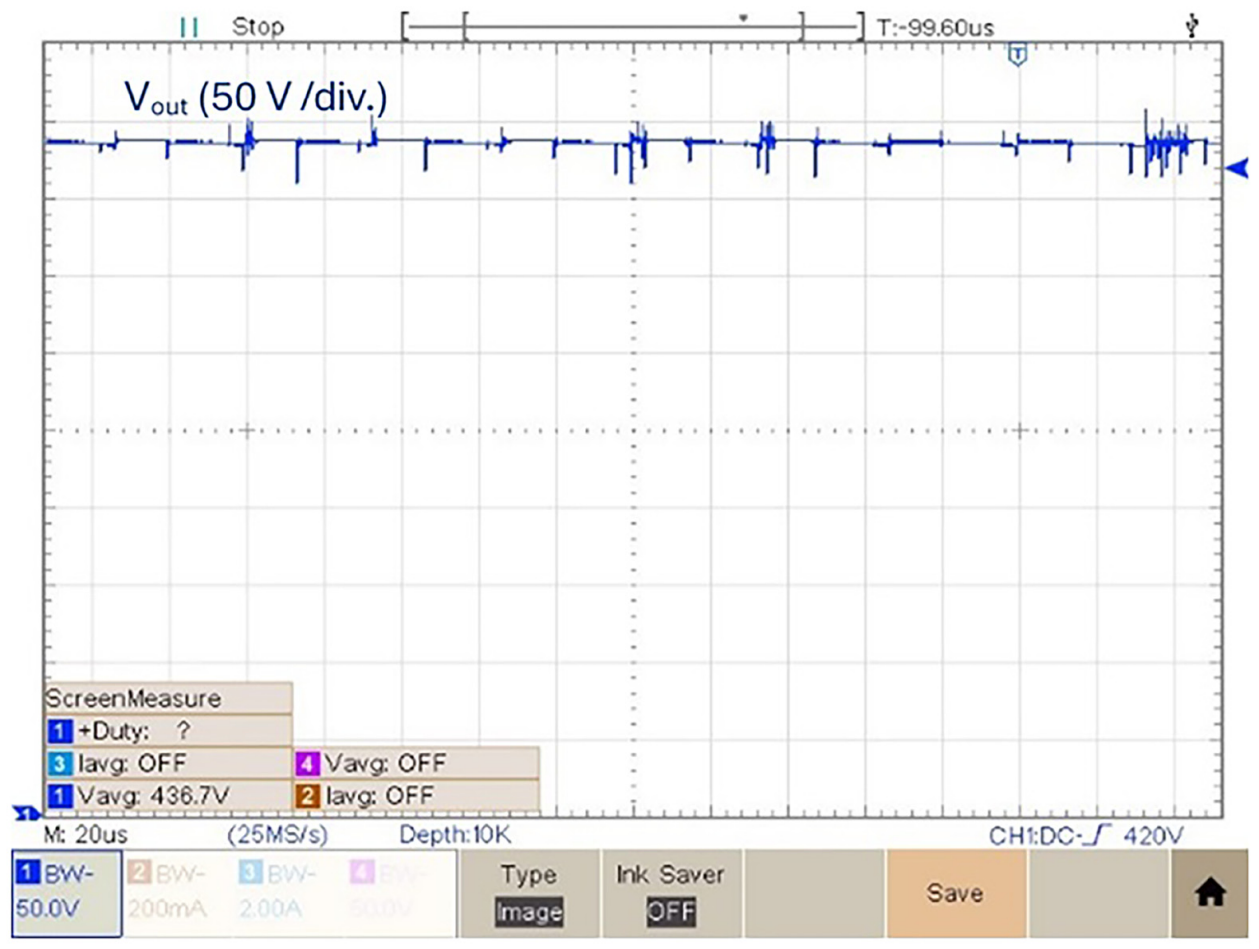
Output voltage for a duty cycle, *δ* = 0.6.

As a final experimental result, the prototype was tested with a duty-cycle value *δ* = 0.6. Figure 18 shows the output voltage in this condition. According to Figure 18, the experimental result obtained of the output voltage obtained with a duty-cycle *δ* = 0.6 is 436.7 V. Which means it is possible to get a voltage gain ( *v _out_/v _in_*) of 8.62. Above this duty-cycle is difficult to increase the voltage gain due to increased losses.

**Figure 19.**
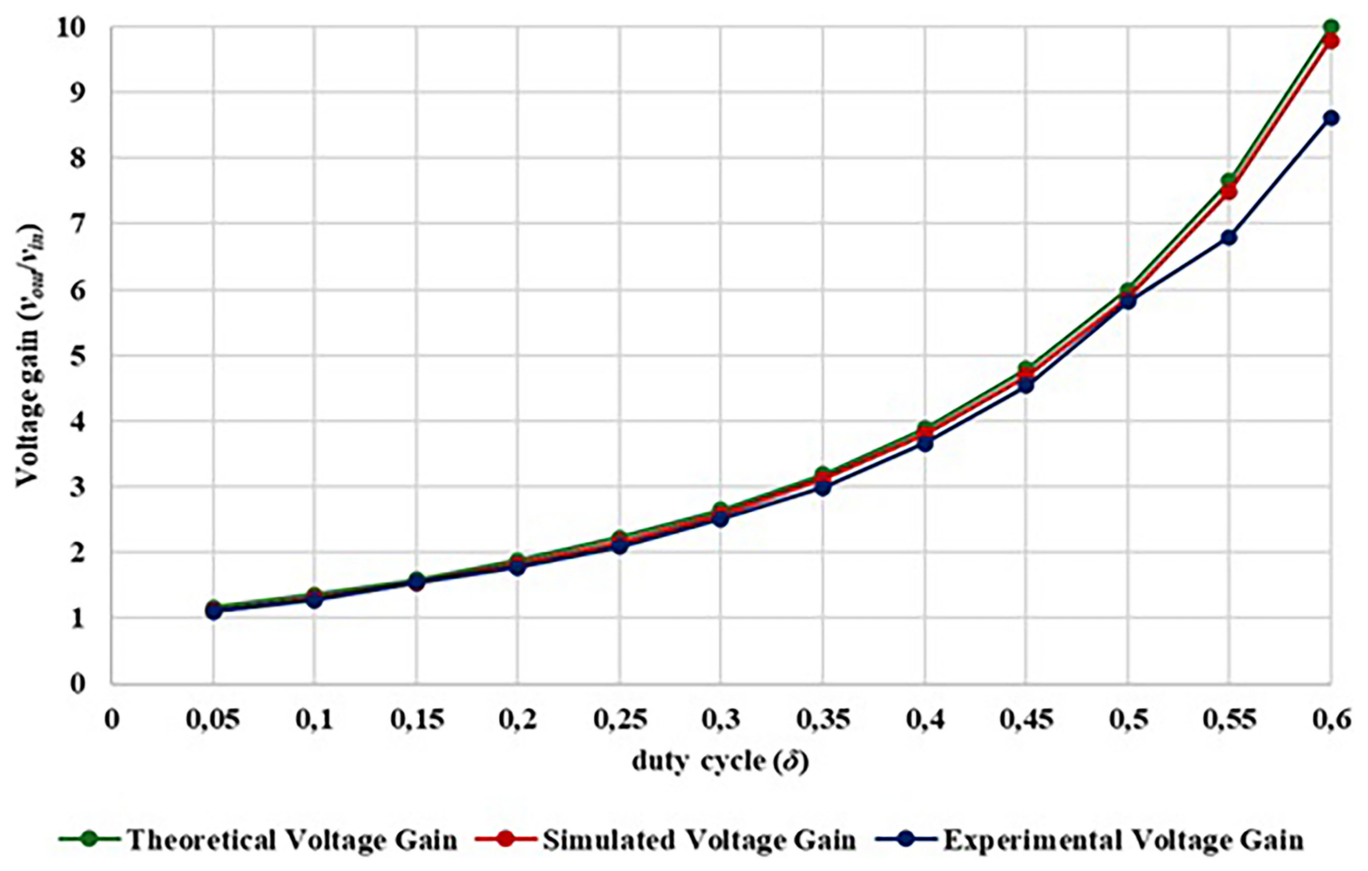
Comparison between theoretical, simulation and experimental voltage gain.

Figure 19 compares the theoretical voltage gain of the proposed converter, the computational simulation results (simulated in PLECS ^®^) and also the experimental voltage gain obtained.

Analyzing Figure 19 is possible to observe in the duty-cycle range from 0.05 to 0.60, the output voltage and voltage gain of the experimental prototype shows a high degree of similarity when compared with the simulation and theoretical calculations. Additionally, it is confirmed that with a duty-cycle of 0.20, a gain voltage greater than 1.7 is achieved, with a duty-cycle of 0.30, a voltage gains greater than 2.5, with a duty-cycle of 0.40, a voltage gains greater than 3.6 and with a duty-cycle of 0.50, a gain greater than 5.8 is achieved. A maximum gain of 8.62 was achieved with a duty-cycle of 0.60. Behind this duty-cycle is difficult to improve the voltage gain since the losses become extremely high.

### Efficiency analysis

This section discusses the results obtained based on experimental test observations, oscilloscope waveforms, and measured voltages and currents. The analysis aims to evaluate the converter’s overall efficiency and to identify the optimal operational point. Both simulated and experimental results are compared to assess the validity of the proposed design.

In experimental prototypes of DC-DC converters, the total efficiency depends not only on ideal power transfer but also on several non-ideal factors such as conduction, switching, and magnetic losses. Since the current and voltage waveforms vary with the duty-cycle ( *δ*), the efficiency and total losses are inherently functions of *δ*. The converter’s efficiency can be expressed as:


$$\;\eta (\delta )=\frac{P_{Output}(\delta )}{P_{Input}(\delta )}=\frac{V_{Out}(\delta )\cdot i_{Out}(\delta )}{V_{in}(\delta )\cdot i_{in}(\delta )}\;(22)$$

During the experimental measurements, the efficiency of the DC-DC converter prototype was evaluated using ( 22), based on the measured input and output voltages and currents.

However, in PLECS ^®^ environment, to better understand the internal loss mechanisms, an equivalent non-ideal circuit model was developed, including parasitic elements such as the MOSFET on-resistance ( *R _DS_*_( *on*)_), diode forward voltage ( *V _F_*), inductor series resistance ( *R _L_*), and capacitor equivalent series resistance ( *R _C_*). The total power loss can then be expressed as:


$$\;P_{Losses}(\delta )=P_{CL,S}(\delta )+P_{CL,D}(\delta )+P_{CL,L}(\delta )+P_{CL,C}(\delta )+P_{SL}(\delta )\;(23)$$

Where:

-
$P_{CL,S}(\delta )=i_{Switch,rms}^{2}(\delta )\cdot R_{DS(on)}$ : Conduction losses in MOSFETs
- *P _CL,D_* ( *δ*) = *V _F_* ⋅ *I _D,avg_* ( *δ*): Conduction losses in diodes
-
$P_{CL,L}(\delta )=i_{L,rms}^{2}(\delta )\cdot R_{L}$ : Conduction losses in inductors
-
$P_{CL,C}(\delta )=i_{C,rms}^{2}(\delta )\cdot R_{C}$ : Conduction losses in capacitors
- *P _SL_* ( *δ*) = 0.5 ⋅ *V _sw_* ( *δ*) ⋅ *I _sw_* ( *δ*) ⋅ ( *t _on_* + *t _off_*) ⋅ *f _sw_*: Switching losses

The current and voltage stress of the power devices are proportional to the duty cycle, which means that at higher *δ* values, both conduction and switching losses increase. Conversely, at lower *δ* values, current ripple and average currents dominate, leading to higher conduction losses in inductors and MOSFETs.

The analytical efficiency model was implemented in PLECS ^®^, using parasitic parameters obtained from datasheets. The simulated efficiency curve exhibits the same trend observed experimentally, where efficiency increases with *δ* up to an optimal operating point, then gradually decreases as both switching and conduction losses become more significant.

In the PLECS ^®^ simulation environment, two dedicated functional blocks were used, one for switching loss calculation and another for conduction loss calculation. The resulting losses were then summed up and applied in ( 24) to obtain overall converter efficiency.


$$\;\eta (\delta )=\frac{P_{Output}(\delta )}{P_{Output}(\delta )+P_{Losses}(\delta )}\;(24)$$

Figure 19 shows the graphical result of the efficiency obtained in both simulation and experimental tests, as function of the converter duty cycle.

**Figure 20.**
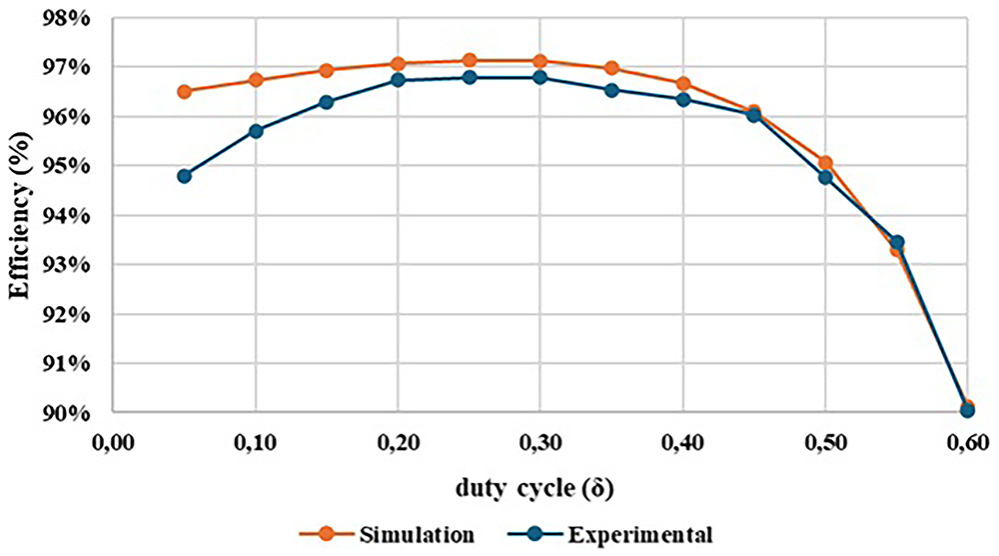
Comparison between the simulation and the experimental results concerning the converter efficiency.

After analysing the results presented in Figure 20, a close correlation is observed between the efficiency variation and the duty-cycle applied to the converter. For a duty-cycle variation from 0.05 to 0.60, an efficiency range around 97% to 90% was obtained in the simulation tests and an efficiency range around 96% to 90% was obtained in the experimental tests. It is also observed that a maximum efficiency of 96.79% was achieved for a duty-cycle δ = 0.25.

**Figure 21.**
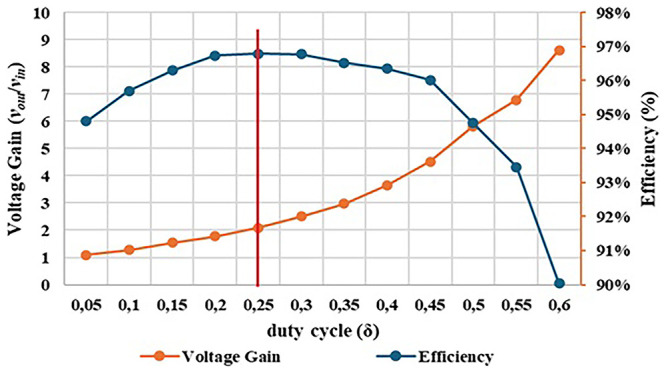
Comparison between experimental voltage gain result and experimental efficiency result. A maximum efficiency of 96.79% was achieved for a duty-cycle around δ = 0.25.

Figure 21 illustrates the evolution of experimental efficiency and voltage gain over the duty cycle. The purpose of this relationship is to evaluate at which output voltage gain value it is possible to achieve the best efficiency, helping us to identify an optimal operating point for the proposed DC-DC converter prototype.

Examining Figure 21 is possible to observe that the optimal operating point of the converter happens with a duty-cycle of δ =0.25 which results in a voltage gain of 2.09 (marked in red in the figure). However, it is clear that, up to a duty-cycle of 0.60, the converter maintains an efficiency between 90% and 96%, which can be considered quite satisfactory. At the maximum value for which it was designed, with a duty-cycle of 0.50, the converter presents a voltage gain of 5.82 and an efficiency of 94.76%. Additionally, there is very little variation in efficiency, as it remains between 96% and 95% until reaching a duty-cycle of 0.50.

**Figure 22.**
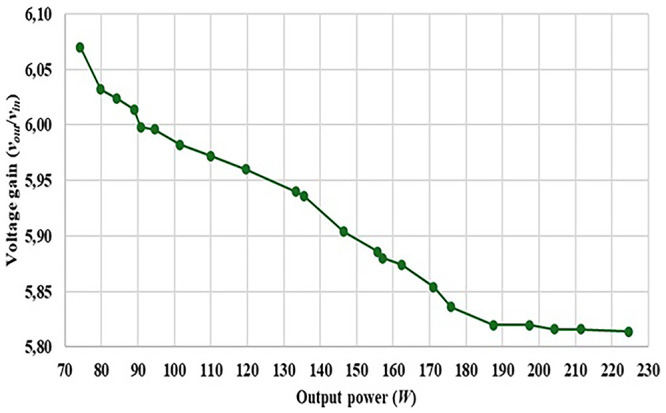
Output voltage gain versus Output Power, considering a fixed duty-cycle of 0.50.

Finally, Figure 22 shows the relation between the output power and the output voltage gain of the proposed topology considering a fixed duty-cycle of 0.50, showing certain limitations over the output gain due to several losses.

**Figure 23.**
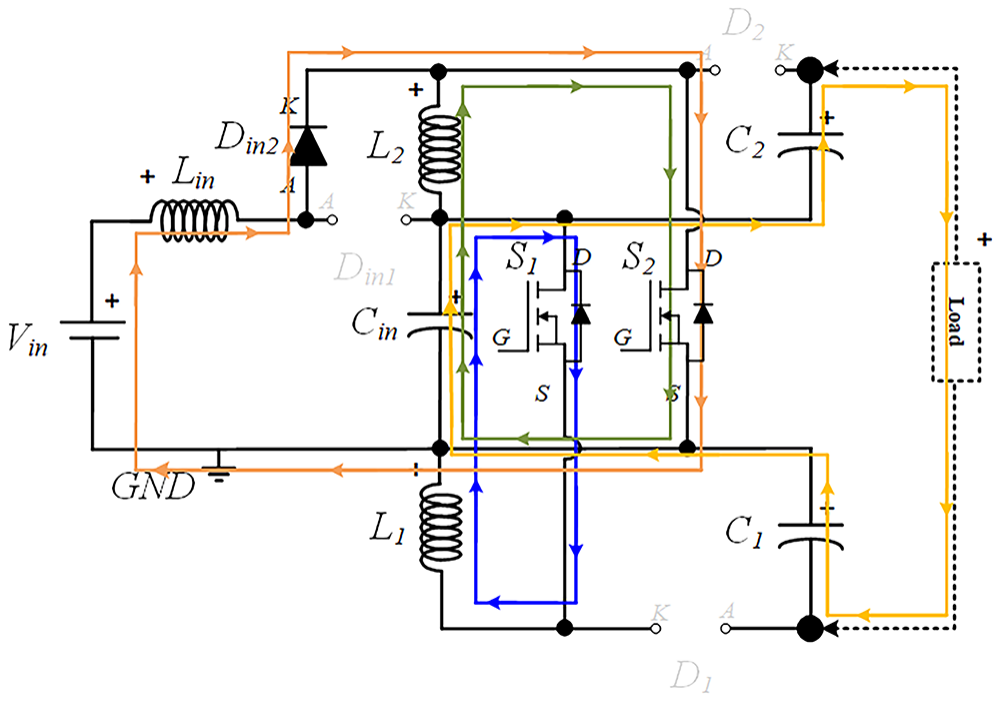
Current flow analysis when both S _1_ and S _2_ are turned ON.

For duty cycles ( *δ*) above 0.5, an additional operating condition appears in which both S1 and S2 conduct simultaneously. This fourth state, shown in Figure 23, creates an overlap between the interleaved channels. The current-loop analysis corresponding to this additional operating mode is illustrated in Figure 23. As observed, *D _1_*, *D _2_*, and *D _in1_* are turned off, whereas *D _in2_* conducts. During this interval, all three inductors store energy (charging mode), while the three capacitors release energy (discharging mode). Although this regime increases the converter’s voltage gain, it also raises the instantaneous current stress on the power devices and amplifies switching losses, as both switches share conduction intervals. Consequently, this overlapping mode is critical for understanding the efficiency degradation observed experimentally for *δ* > 0.5.

**Figure 24.**
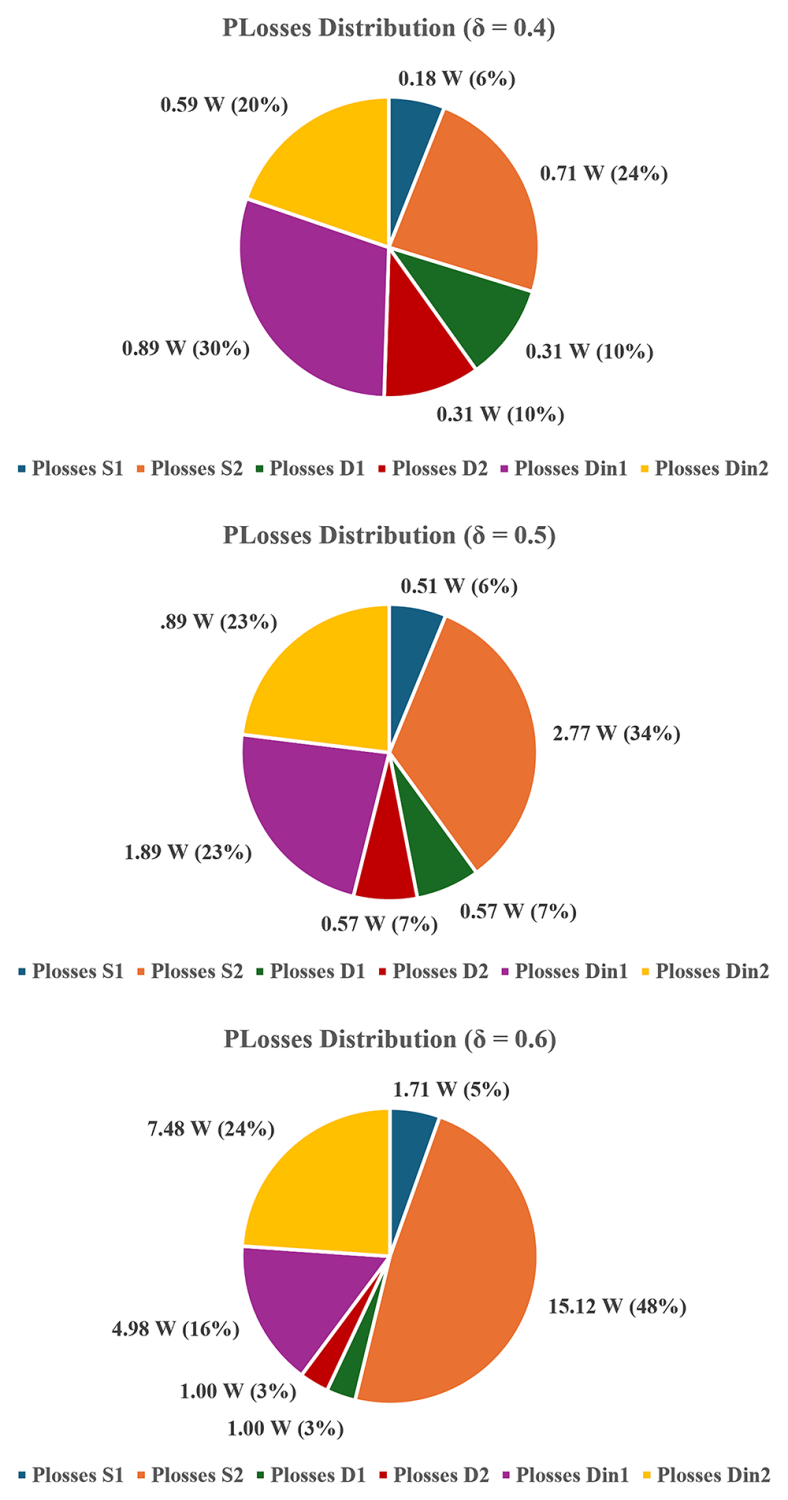
Simulated diodes and MOSFETs power losses (PLECS ^®^) for δ = 0.4–0.6.

**Figure 25.**
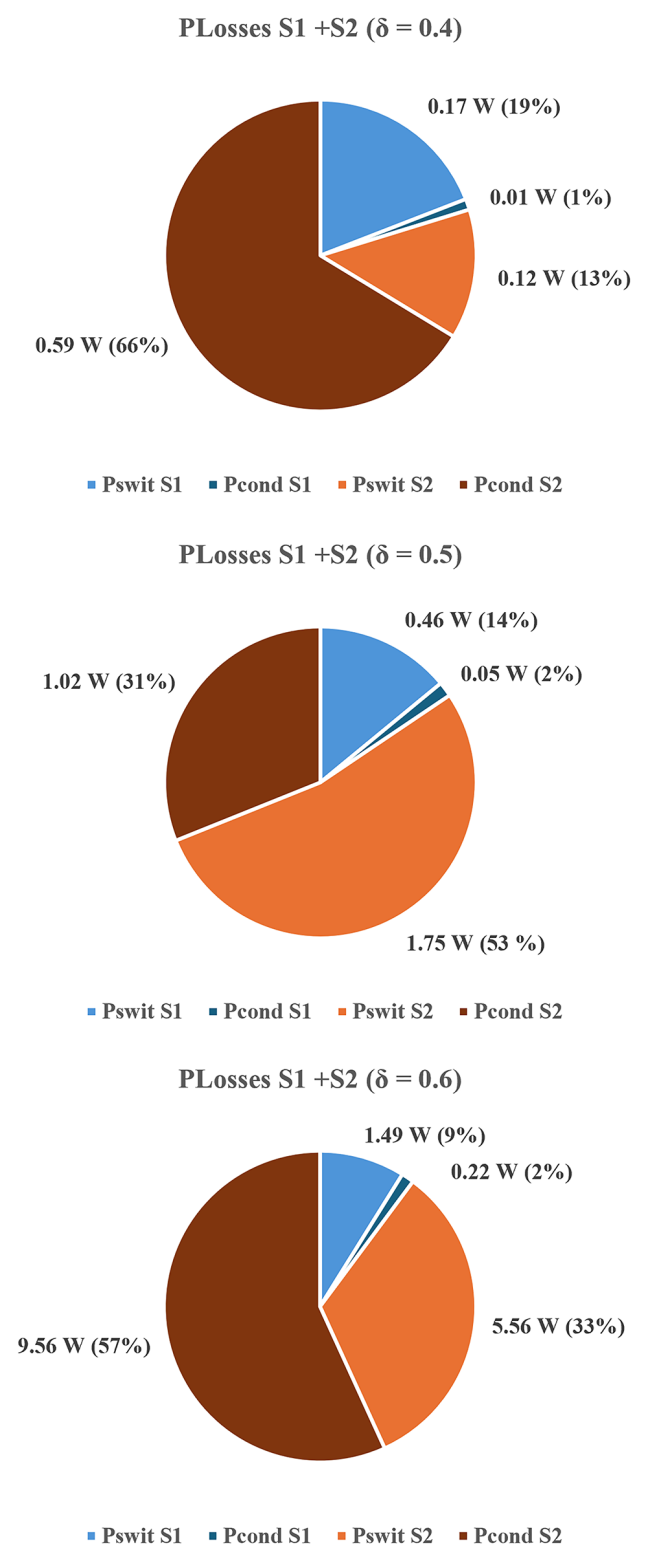
Switching and conduction losses in both MOSFETs (PLECS ^®^) for δ = 0.4–0.6.

To quantify these effects, Figure 24 and Figure 25 present the simulated loss distribution obtained from PLECS ^®^ for *δ* = 0.4, 0.5, and 0.6. Figure 24 shows the total losses per component ( *S _1_*, *S _2_*, *D _1_*, *D _2_*, *D _in1_*, *D _in2_*), where it details the switching and conduction losses of both MOSFETs. Two dedicated PLECS ^®^ functional blocks, one for switching losses estimation and another for conduction losses estimation were employed. As confirmed before in Figure 20, simulated efficiency curve follows the same pattern as the experimental curve, with slightly higher values due to idealized parasitic parameters and datasheet values used in the modeling. Although, the similarity between simulated and experimental efficiency results confirms the high accuracy of the simulation model and provides confidence in the reliability of the simulated data.

The results clearly indicate that losses increase with the duty-cycle and that their distribution becomes progressively unbalanced. At *δ* = 0.4, total power losses are relatively balanced between switches and diodes. At *δ* = 0.5, *S _2_* already exhibits higher conduction and switching losses, and at *δ* = 0.6 this imbalance becomes dominant, with S _2_ accounting for nearly half (48%) of the total dissipation. This asymmetry between *S _1_* and *S _2_* arises from differences in their respective current paths. As shown in Figure 3 ( *S _1_* ON) and Figure 5 ( *S _2_* ON), as well as in Figure 23 (both switches ON), the conduction path associated with *S _2_* consistently carries higher current levels, resulting in greater conduction losses. Regarding switching losses, *S _2_* also experiences higher voltage stress during turn-on and turn-off transitions due to capacitor interactions and interleaving effects.

The overlapping conduction mode depicted in Figure 23 reinforces these findings: simultaneous operation of *S _1_* and *S _2_* produces higher current peaks through both inductors and diodes, leading to increased switching and power losses. Although this mode extends the voltage gain, it compromises efficiency and thermal balance. Therefore, in practical implementations, operation beyond *δ* = 0.5 should be avoided.

In a nutshell, the converter achieves a maximum efficiency of approximately 96.8% at δ ≈ 0.25, where both conduction and switching losses are minimized. For δ > 0.5, the increased switching transitions and diode recovery effects contribute to a measurable reduction in efficiency.

The results confirm that the proposed interleaved quadratic boost converter maintains high efficiency and stable performance across a wide duty-cycle range, validating both the analytical and experimental findings. This reinforces its suitability for applications such as PV microgrids and other RES systems, where operation under varying input and duty conditions is common.

### Voltage gain and efficiency comparison with other topologies

This section aims to compare the experimental performance of the proposed converter with the same topologies previously reported in Table 3, specifically those presented in references ^52– 56^. Table 5 summarizes the main operating characteristics of each converter, including input voltage, maximum output voltage, corresponding duty-cycle, voltage gain, rated power, and efficiency. The comparison highlights the performance of the proposed converter relative to existing designs, particularly in terms of voltage gain, efficiency, and prototype rated power achieved.

**Table 5.** Experimental voltage gain and efficiency comparison between some different interleaved quadratic Boost DC-DC topologies.

	*Topologies*					
	*Proposed*	*52*	*53*	*54*	*55*	*56*
*Input Voltage*	*50 V*	*10 V*	*24 V*	*20 V*	*57 V*	*12 V*
*Maximum Output Voltage obtained*	*436.7 V* *(δ = 0.60)*	*40 V* *(δ = 0.40)*	*380 V* *(δ = 0.65)*	*240 V* *(δ = 0.50)*	*400 V* *(δ = 0.62)*	*120 V* *(δ = 0.70)*
*Maximum Voltage Gain obtained (v _out_/v _in_)*	*8.62* *(δ = 0.60)*	*4.00* *(δ = 0.40)*	*15.83* *(δ = 0.65)*	*12.00* *(δ = 0.50)*	*7.02* *(δ = 0.62)*	*10.00* *(δ = 0.70)*
*Converter’s rated Power*	*200 W*	*100 W*	*100 W*	*300 W*	*-*	*500 W*
*Rated power Efficiency obtained*	*94.76%*	*95.60%*	*92.50%*	*95.50%*	*-*	*91.2%*
*Maximum Efficiency obtained*	*96.79%* *(26.6 W)*	*-*	*-*	*96.25%* *(50 W)*	*-*	*96.80%* *(150 W)*

The comparison between the analyzed topologies reveals that the proposed converter does not achieve the highest experimental voltage gain, this is obtained in 53 with a gain of 15.83 at *δ* = 0.65, but with a lower rated power of 100 W and a lower efficiency of 92.50%. While the proposed prototype demonstrates a balanced trade-off between voltage gain, efficiency, and power capability. With an input voltage of 50 V, the proposed topology reaches a maximum output voltage of 436.7 V, with a voltage gain of 8.62 at *δ* = 0.60, delivering 200 W with a measured efficiency of 94.76% and a peak efficiency of 96.79% at 26.6 W.

Compared to other converters, the proposed converter operates with a higher input voltage and still maintains high efficiency across a wide duty-cycle range. In contrast, converters in 52– 54 and 56 achieve their voltage gains at lower input voltages (10 to 24 V). The topology in 55 is limited to simulation results, and 56 achieves similar maximum efficiency (96.80%) under a higher power of 150 W.

Overall, the proposed converter exhibits a competitive performance, combining relatively high voltage gain and efficiency with moderate duty-cycle operation. This balance between efficiency, voltage gain, and practical scalability makes the proposed topology a suitable converter to use in RES and DC microgrid applications where both voltages gain, and efficiency are required.

### PI output voltage control (Simulation test setup)

**Figure 26.**
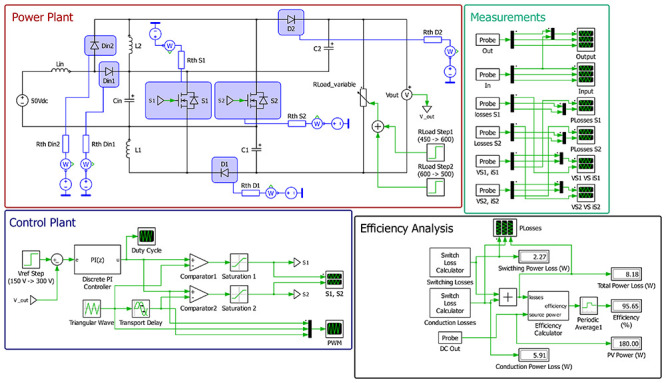
PLECS ^®^ schematic of the proposed DC/DC converter applying a closed-loop PI output voltage control.

To evaluate the closed-loop dynamic performance of the proposed interleaved quadratic boost converter, a PI output voltage control scheme was implemented in the PLECS ^®^ simulation environment. The objective of this control stage is to regulate the DC output voltage under varying load and reference conditions, ensuring stability and fast transient response. Figure 26 presents the integral scheme of the PLECS ^®^ system created for the tests.

As illustrated in Figure 26, the control strategy employs a proportional–integral (PI) controller in a classical feedback configuration, where the output voltage is continuously compared with a predefined reference. The resulting error is processed by the PI compensator, which adjusts the duty-cycle of both switches to maintain the output voltage at the desired level. The reference voltage and load conditions were varied over time to assess the controller’s robustness and adaptability.

To evaluate the converter’s dynamic performance under closed-loop control, the simulation begins at 0.0 s to 0.4 s, where the voltage reference is initially set to 150 V with a 450 Ω load (50 W). Then, from 0.4 s to 0.8 s, the reference voltage is increased from 150 V to 300 V while maintaining the same load of 450 Ω (200 W). From 0.8 s to 1.2 s, the load is changed from 450 Ω to 600 Ω, corresponding to a power reduction from 200 W to 150 W. Finally, from 1.2 s to 1.6 s, the load varies again from 600 Ω to 500 Ω, resulting in an increase in power from 150 W to 180 W.

These test conditions were designed to correctly emulate typical operating scenarios encountered in RES systems, where both the output voltage reference and the load demand may change dynamically. Both proportional and integral controller parameters were tuned to achieve minimal overshoot and a fast-settling time while maintaining stability across all operating conditions.

**Figure 27.**
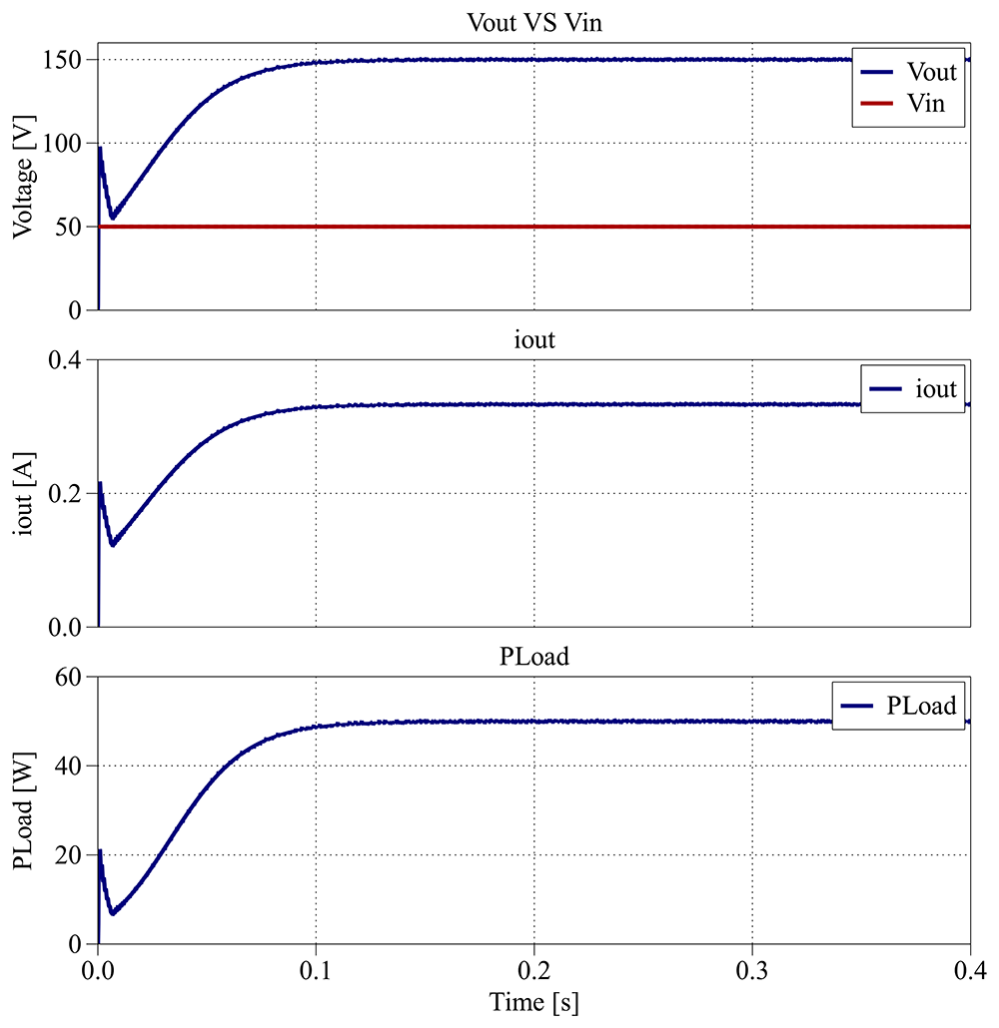
PLECS ^®^ result for closed-loop control between 0.0 s and 0.4 s.

Figure 27 presents the input voltage ( *v _in_*), output voltage ( *v _out_*), output current ( *i _out_*) and the output load power ( *P _Load_*) results during the interval between 0.0 s and 0.4 s.

As shown in Figure 27, the output voltage accurately follows the 150 V reference defined in the PI compensator, settling within about 0.1 s.

**Figure 28.**
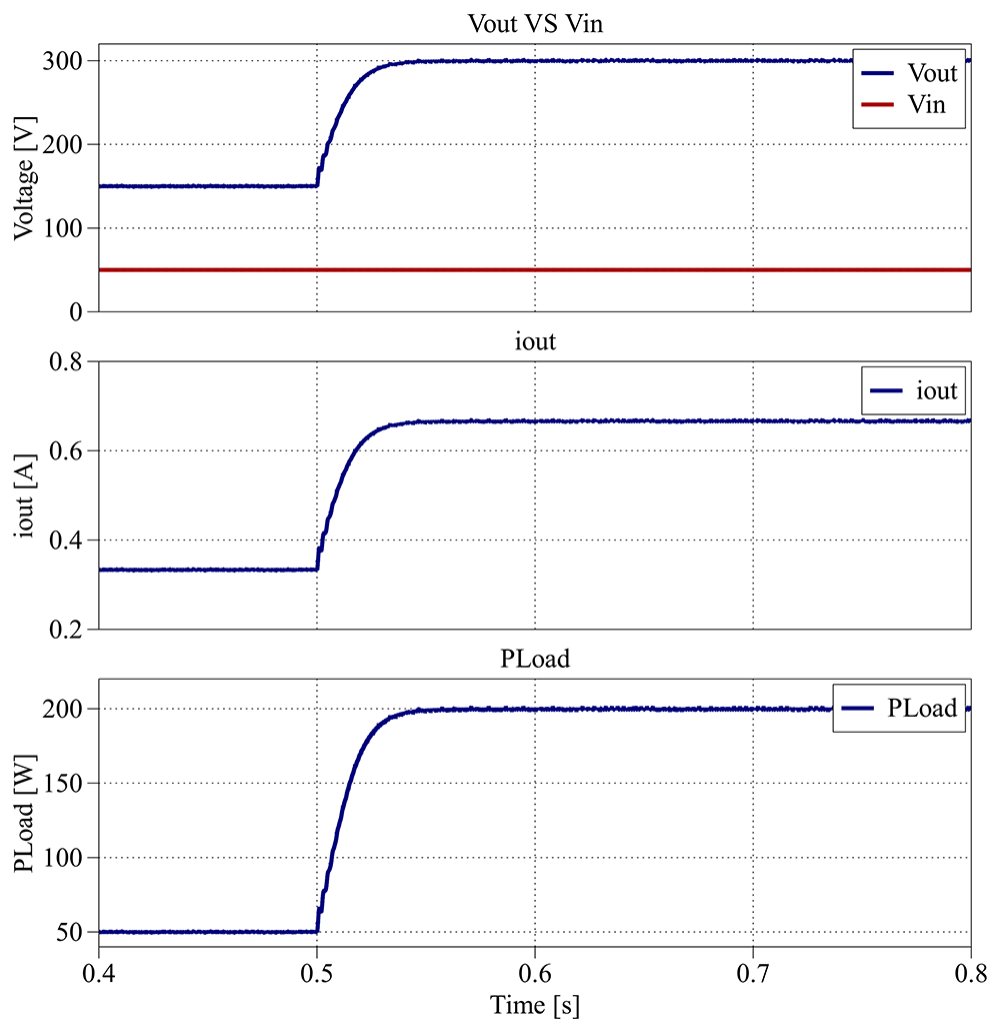
PLECS ^®^ result for closed-loop control between 0.4 s and 0.8 s.

Figure 28 illustrates the dynamic response of the proposed converter between the interval from 0.4 s to 0.8 s, when the voltage reference is increased to 300 V while maintaining the same load.

The output voltage again follows the new reference within 0.1 s, with no observable overshoot or oscillation. Both Figure 27 and Figure 28 demonstrate that the converter achieves a smooth transient response and robust reference tracking.

**Figure 29.**
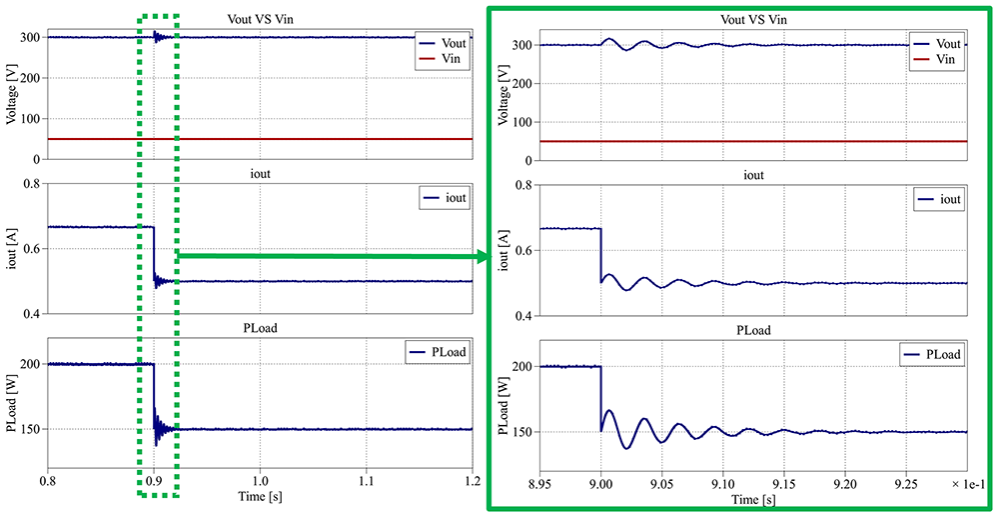
PLECS ^®^ result for closed-loop control between 0.8 s and 1.2 s.

Figure 29 depicts the converter behavior between 0.8 s and 1.2 s, when the load decreases from 450 Ω (200 W) to 600 Ω (150 W).

Despite the reduction in load, the output voltage remains regulated around 300 V, with a maximum deviation between 285 V and 315 V (±5%) and a settling time of less than 0.25 s. The output current exhibits a slight variation between 0.48 A and 0.53 A (±5%), consistent with the load change.

**Figure 30.**
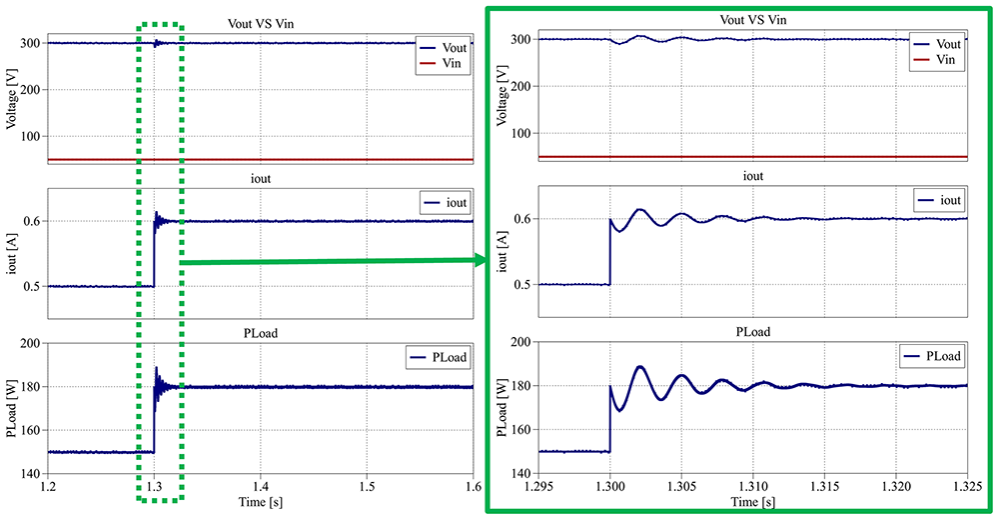
PLECS ^®^ result for closed-loop control between 1.2 s and 1.6 s.

Figure 30 provides the converter’s main parameters for the interval from 1.2 s to 1.6 s, where the load increases from 600 Ω (150 W) to 500 Ω (180 W).

Once again, in Figure 30, the output voltage remains stable around the 300 V reference, with a smaller variation of about ±3% (290 V to 308 V) and a settling time of approximately 0.20 s. The corresponding output current varies between 0.58 A and 0.62 A (±3%), reflecting smooth transient behavior.

Throughout all simulation intervals, the converter maintains an efficiency between 95.6% and 96.5%, confirming the effectiveness of the closed-loop PI control in preserving both dynamic stability and high efficiency under varying reference and load conditions.

### MPPT PV Control (Simulation test setup)

To evaluate the converter’s performance when supplied by a photovoltaic (PV) source, an MPPT (Maximum Power Point Tracking) control algorithm was implemented in the PLECS ^®^ simulation environment. The objective of this test is to assess the converter’s ability to dynamically extract the maximum available power from the PV array (input voltage) while maintaining stable voltage regulation under varying irradiance and temperature conditions.

**Figure 31.**
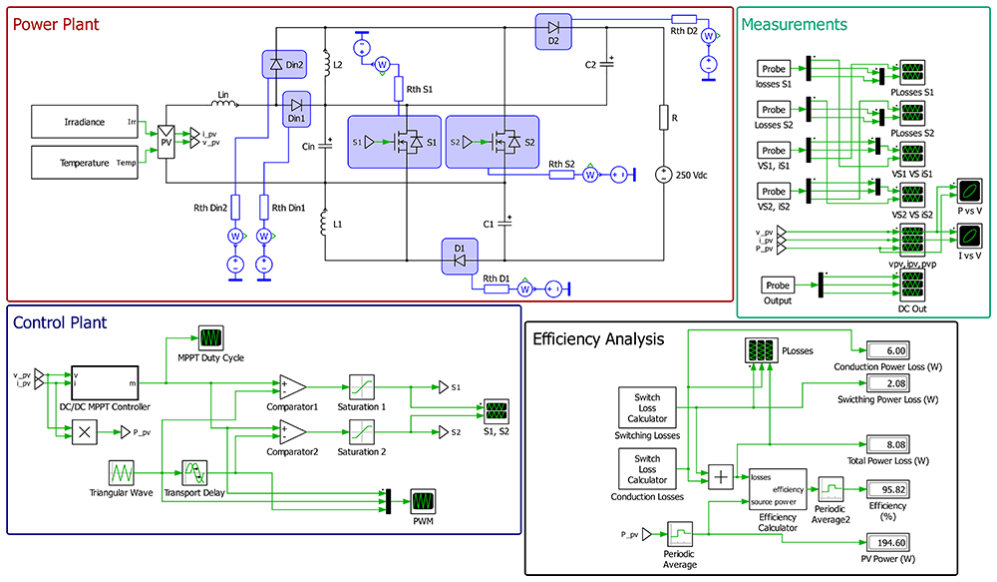
PLECS ^®^ schematic of the proposed DC/DC converter applying a MPPT PV control.

The simulation setup consists of a PV source modeled according to manufacturer datasheet parameters, connected to the input of the proposed converter. The converter operates under a single closed-loop control stage, where the MPPT algorithm directly generates the duty-cycle applied to both switches. Two common MPPT algorithms, Perturb and Observe (P&O) and Incremental Conductance (IC), were tested under identical conditions, both demonstrating nearly identical performance in terms of tracking speed, accuracy, and stability. Therefore, only the P&O algorithm results are presented in this section for clarity and conciseness. Figure 31 presents the integral scheme of the PLECS ^®^ system created for the tests.

The PV array modeled in PLECS ^®^ consists of a string of three BP 365 photovoltaic modules connected in series. Each module has a maximum power point (MPP) power of 65 W, with an MPP voltage of 17.6 V and an MPP current of 3.69 A. The resulting string produces a total power of approximately 195 W at an MPP voltage of 52.8 V under standard test conditions (STC: 1000 W/m ^2^, 25°C).

A 250 V voltage source was also connected at the converter output to emulate a DC-link bus typically controlled by another converter stage. This setup allows evaluation of the proposed converter’s MPPT performance independently from the DC-link voltage control, focusing only on the solar PV energy extraction and dynamic response.

During the simulation, the PV irradiance and temperature were dynamically changed to emulate realistic environmental conditions. From 0.0 s to 0.4 s, the irradiance was set to 1000 W/m ^2^ with a temperature of 25°C, representing standard test conditions. Between 0.4 s and 0.8 s, the irradiance was reduced to 700 W/m ^2^ while maintaining the same temperature. From 0.8 s to 1.2 s, the irradiance returned to 1000 W/m ^2^ at 25°C, and finally, during the last interval, from 1.2 s to 1.6 s, the irradiance remained constant at 1000 W/m ^2^, but the temperature increased to 40°C.

**Figure 32.**
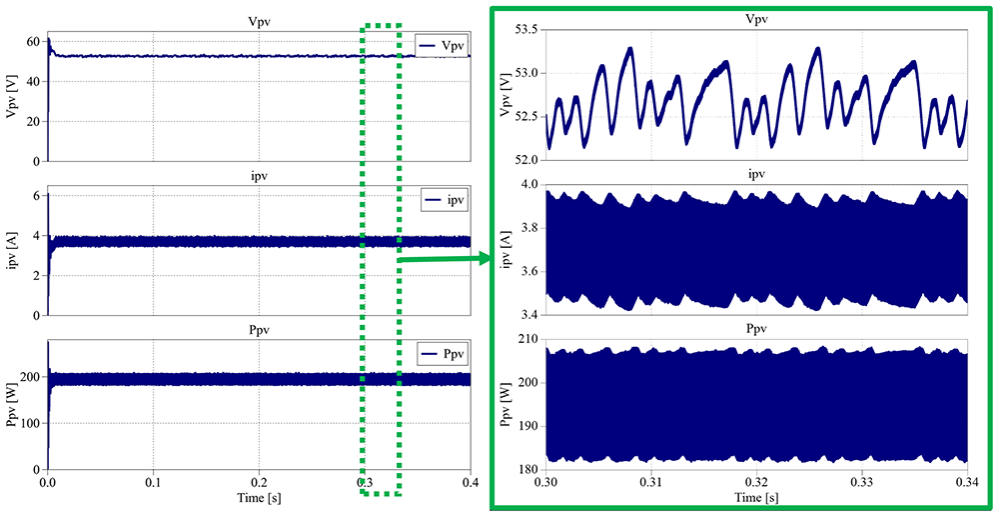
PLECS ^®^ result for MPPT PV control between 0.0 s and 0.4 s.

The first simulation interval, between 0.0 s and 0.4 s, is presented in Figure 32. In this section, each result will show the PV voltage ( *v _pv_*), PV current ( *i _pv_*) and the PV power ( *P _pv_*).

**Figure 33.**
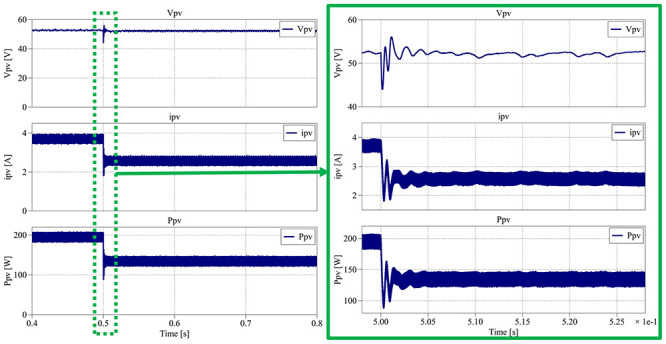
PLECS ^®^ result for MPPT PV control between 0.4 s and 0.8 s.

The results obtained in Figure 32 show that the PV voltage is placed between 53.3 V and 52.2 V, resulting in an average voltage of 52.8 V (±1%), the same value as the MPP voltage presented in the datasheet (17.6 V × 3). The PV current varies between 3.43 A and 3.95 A, translating in an average current of 3.69 A (±7%), matching the MPP voltage presented in the datasheet. The PV power waveform is between 182 V and 208 V, which means an average power of 195 W (±7%), exactly same as the datasheet. The next simulation interval is between 0.4 s and 0.8 s, where the irradiance decreases from 1000 W/m ^2^ to 700 W/m ^2^, as depicted in Figure 33.

According to the results in Figure 33, following the irradiance step-down, the PV power decreases from 195 W to approximately 134.5 W and stabilizes in less than 0.05 s. The PV voltage remains nearly constant at 52.2 V, while the current decreases to an average of 2.58 A, consistent with the expected irradiance reduction.

**Figure 34.**
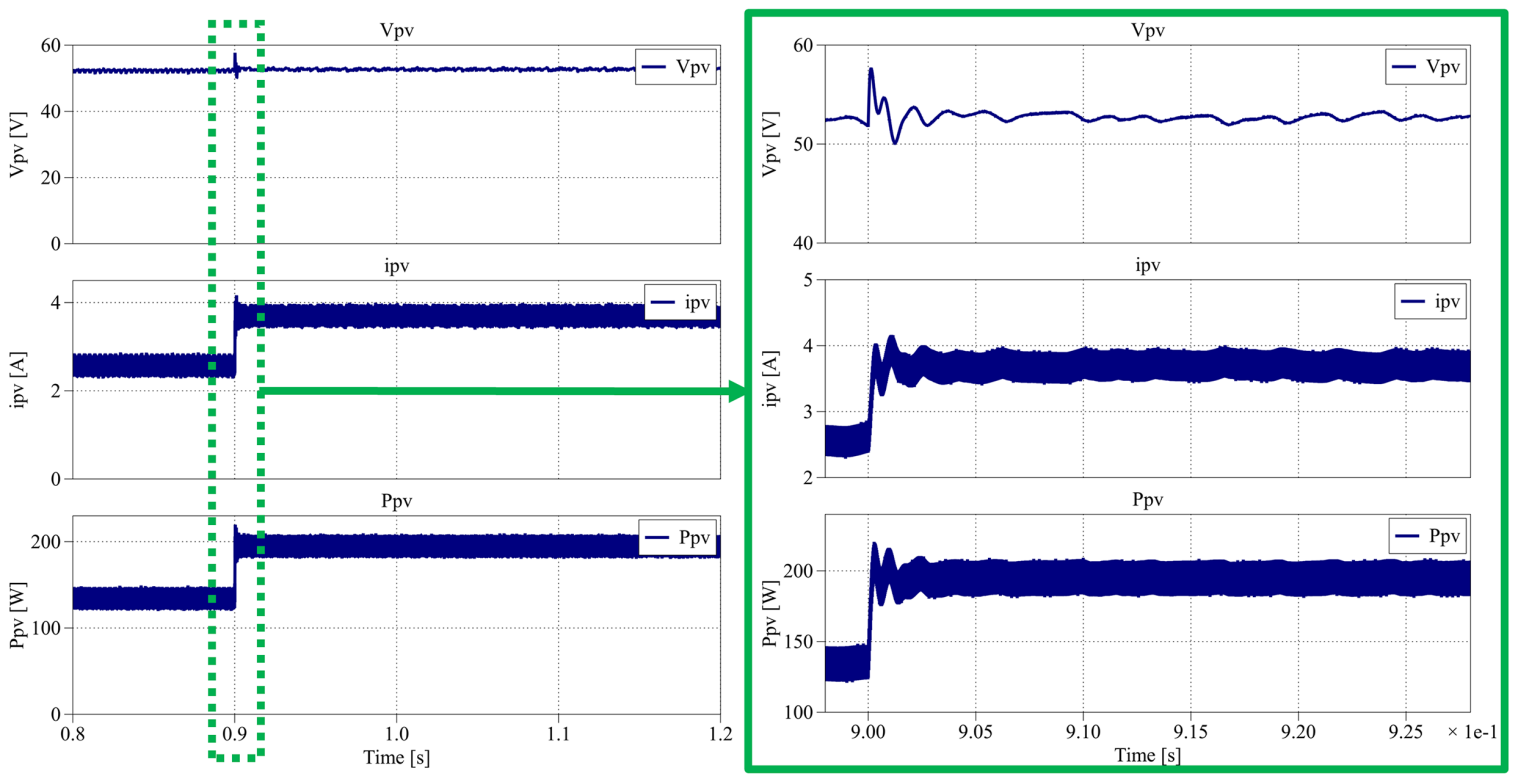
PLECS ^®^ result for MPPT PV control between 0.8 s and 1.2 s.

Figure 34 presents the waveform results from 0.8 s to 1.2 s, when the irradiance returns from 700 W/m ^2^ back to the initial 1000 W/m ^2^.

As before, the converter successfully tracks the maximum power point after the irradiance increase, recovering the PV generation to its nominal level. The PV voltage stabilizes at an average of 52.8 V, and the current returns to 3.69 A, both within 0.05 s, demonstrating the fast and stable response of the MPPT controller.

**Figure 35.**
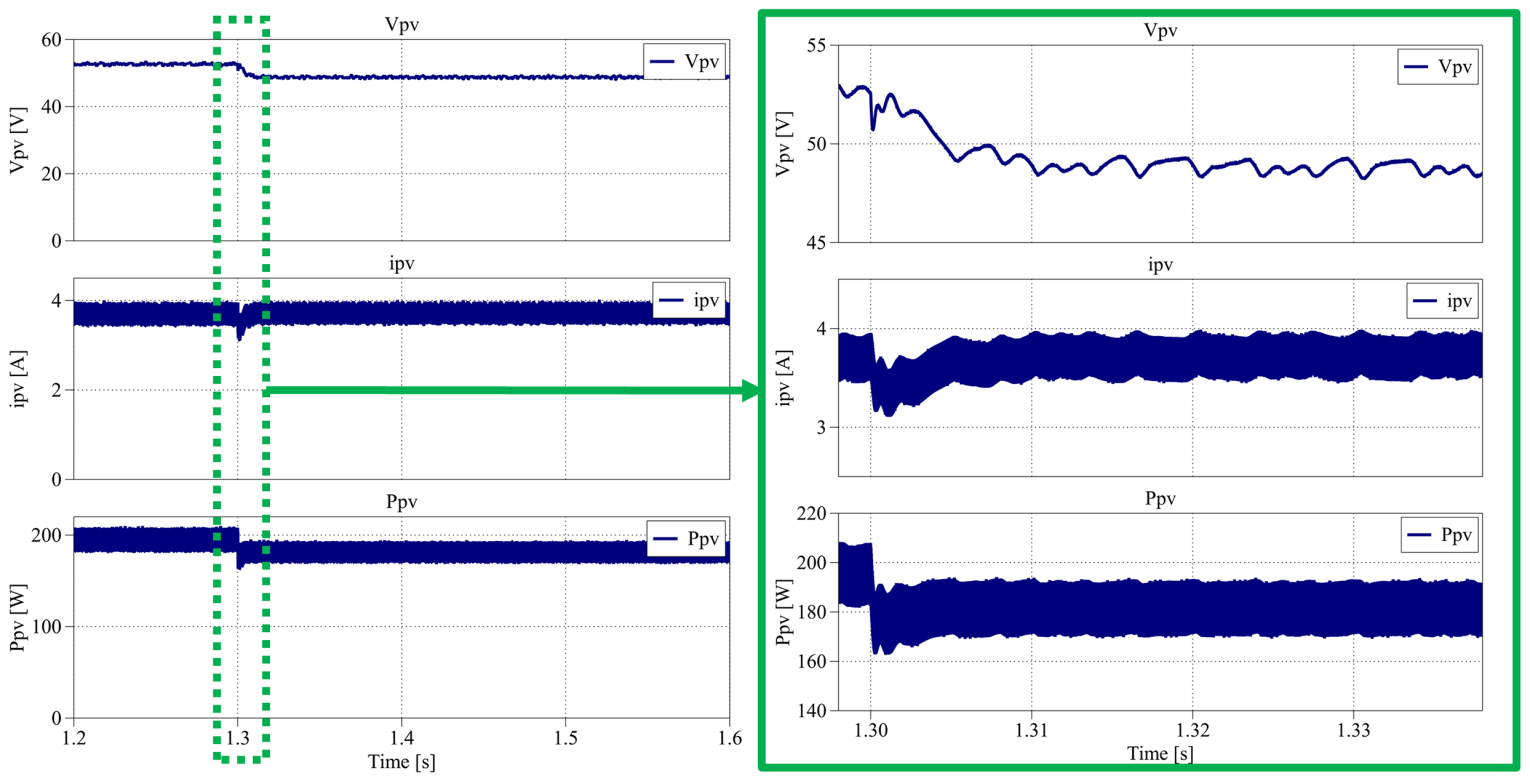
PLECS ^®^ result for MPPT PV control between 1.2 s and 1.6 s.

Finally, Figure 35 shows the results obtained between 1.2 s and 1.6 s, where the irradiance remains constant in the 1000 W/m ^2^ while the temperature is increased to 40 ºC.

As shown in Figure 34, the increase in temperature causes the PV voltage to decrease to an average of 48.7 V (approximately –8% compared to STC), while the current slightly increases to 3.72 A (+1% compared to STC). These variations are consistent with the well-known temperature dependence of PV cells, where higher temperatures reduce the open-circuit voltage and slightly increase the short-circuit current. 

Across all simulated intervals, the converter consistently achieved efficiencies ranging from 95.6% to 96.1%, demonstrating the MPPT PV control’s capability to maintain dynamic stability and high efficiency under fluctuating irradiance and temperature conditions.

## Discussion

The proposed interleaved quadratic boost DC-DC converter demonstrated robust performance and consistent behavior across simulation and experimental stages. The component stress analysis confirmed that the voltage stress over each power switch was reduced compared to other similar proposed topologies in the literature, with the maximum voltage stress over power devices *S _1_* and *S _2_* as *v _Cin_ + v _C1_* and *v _Cin_ + v _C2_*, respectively.

There is still constant research regarding the design of new DC-DC converter topologies with high-voltage gain ratio and boost ability to extend the operation of RES, and other sources, all over the available voltage ranges, extracting efficiently as much energy as possible. In this article, it was made a brief research about other types of DC-DC converters and it was decided to create a new interleaved quadratic DC-DC converter topology.

Quadratic DC-DC converters are some of the topologies that can achieve high voltage gains and are the most suitable for several RES applications due to the variability of most of them, which are dependent on weather conditions, location, distribution system and other aspects. When compared with other topologies in the literature, especially other quadratic DC-DC converters, the proposed topology is the one with higher voltage gain but presents a reduced number of components and the interleaved solution allows to reduce the voltage and current stress over power devices which allows to increase the reliability of the solution.

The laboratory prototype was tested in several conditions during several days to evaluate the overall performance, namely the voltage and current stress, robustness, overheating issues, hot spots, electromagnetic noise, efficiency, sensitivity to parameters variation and other aspects.

Regarding the electromagnetic noise, some adjustments need to be made to future work on the printed circuit board and components, but the overall performance is quite acceptable. This will allow us to improve some waveform and interference due to electromagnetic noise.

Efficiency was measured in several conditions and real values between 90% (worst conditions) and 96% (best condition) were achieved. Its analysis revealed that conduction and switching losses increase with duty-cycle, especially when both switches overlap in conduction for *δ* > 0.5. Loss modeling in PLECS ^® ^confirmed that *S _2_* experiences higher conduction and switching stress due to its current path characteristics. Nevertheless, the correlation between simulated and experimental data was high, confirming the precision of the analytical model.

Another relevant aspect is the reduced capacitance of capacitors, due to the interleaved operation. This leads to reduced stress over capacitors and distributed voltage. Notice that the output voltage is the sum of the voltage over the three capacitors.

A low power laboratory prototype was developed but it is possible to develop a similar converter with higher power density.

Dynamic performance tests under closed-loop PI control demonstrated good voltage regulation and transient response. For output reference steps from 150 V to 300 V, the converter exhibited fast settling (around 0.1 s), minimal overshoot (<5%), and maintained efficiency above 95%. Under load variations, voltage deviations remained within ±5%, ensuring operational stability and adaptability. Furthermore, the MPPT PV control simulations confirmed the converter’s ability to effectively extract maximum power from photovoltaic arrays under varying irradiance and temperature. The Perturb and Observe (P&O) algorithm maintained the PV voltage within 1% of its MPP reference and achieved full recovery to MPPT stage within 0.05 s after irradiance changes, confirming the converter’s capability to operate in PV systems.

## Conclusions

This article proposed a new interleaved quadratic DC-DC boost converter topology with high-voltage gain, including the first theoretical operation analysis, design and further experimental validation, using a 200 W laboratory prototype operating at 50 kHz. The experimental results demonstrate that the proposed topology allows higher voltage gains than most well-known quadratic topologies, reaching a voltage gain from six to more than eight in a real prototype without compromising the efficiency significantly. The proposed converter provides continuous input and output current and a simple PWM control strategy. It was possible to optimize the converter's efficiency by adjusting the duty cycle, which is important when we want to minimize conduction and switching losses.

The results obtained indicate that there is an optimal operating point where efficiency is maximized, achieving an efficient balance between voltage gain and associated losses. Additionally, there is no significant variation in efficiency, which remains between 95% and 96% up to a duty-cycle of 0.50, where a voltage gain of 5.82 is achieved in a real setup. However, when the voltage gains increase behind this point, efficiency began to decrease, resulting in an evident trade-off between maximizing voltage gain and energy efficiency. This trade-off is particularly relevant in solar photovoltaic applications, where it is necessary to find an appropriate compromise between voltage gain and efficiency based on the specific requirements of the system.

Furthermore, the converter can effectively combine a high step-up ratio with simple control and robust dynamic response, as confirmed by both PI output voltage control and MPPT PLECS ^®^ simulations. Under varying irradiance and temperature conditions, the converter maintained accurate power point tracking and stable output voltage, confirming its suitability for photovoltaic integration and energy management in DC powered systems.

## Ethics and consent

Ethical approval and consent were not required.

## Data Availability

No data associated with this article.
