# Supplementary material for: Interleaved Quadratic Boost DC-DC Converter with Extended Voltage Gain and Reduced Switch Voltage Stress for Photovoltaic Applications
Source: Open Res Eur. 2025 Oct 28;5:55. Originally published 2025 Feb 24. [Version 2] doi: 10.12688/openreseurope.19625.2 (PMC12614317; doi:10.12688/openreseurope.19625.2)
Supplement: Peer review history

# Reviewer report: Reviewer response for version 2

**DOI:** 10.21956/openreseurope.23466.r62941

**Published:** 2025-11-06

**Authors:** Siva Asapu

**Affiliations:** 1 Shri Vishnu Engineering College for Women, Bhimavaram, Andhra Pradesh, India

I am satisfied with the revised version of the article. The authors have adequately addressed the previous comments and incorporated the necessary revisions into the manuscript. I have no further comments to make.

Is the study design appropriate and does the work have academic merit?

Yes

Is the work clearly and accurately presented and does it cite the current literature?

Partly

If applicable, is the statistical analysis and its interpretation appropriate?

Yes

Are all the source data underlying the results available to ensure full reproducibility?

Yes

Are the conclusions drawn adequately supported by the results?

Yes

Are sufficient details of methods and analysis provided to allow replication by others?

Partly

Reviewer Expertise:

DC - DC converters, MPPT  and multilevel inverters

I confirm that I have read this submission and believe that I have an appropriate level of expertise to confirm that it is of an acceptable scientific standard.

# Reviewer report: Reviewer response for version 1

**DOI:** 10.21956/openreseurope.21228.r55982

**Published:** 2025-08-08

**Authors:** Rachananjali K

**Affiliations:** 1 Vignan Foundation for Science Technology & Research, Guntur, Andhra Pradesh, India

Authors have addressed the issues and focused on extended voltage gain and reduced switch count.

 Topology is being implemented for photovoltaic application. Their investigation method was based on an initial theoretical approach using mathematical equations to describe the operation of the electrical circuit and evaluate the performance compared to other topologies, followed by the validation through some computational simulations using MATLAB/SIMULINK software.

 Next, the operation of the proposed converter was also confirmed by several experimental tests using a laboratory prototype developed exclusively for these tests.

Is the study design appropriate and does the work have academic merit?

Yes

Is the work clearly and accurately presented and does it cite the current literature?

Yes

If applicable, is the statistical analysis and its interpretation appropriate?

Yes

Are all the source data underlying the results available to ensure full reproducibility?

Yes

Are the conclusions drawn adequately supported by the results?

Yes

Are sufficient details of methods and analysis provided to allow replication by others?

Yes

Reviewer Expertise:

Renewable and converters

I confirm that I have read this submission and believe that I have an appropriate level of expertise to confirm that it is of an acceptable scientific standard.

# Reviewer report: Reviewer response for version 1

**DOI:** 10.21956/openreseurope.21228.r54070

**Published:** 2025-06-12

**Authors:** Siva Asapu

**Affiliations:** 1 Shri Vishnu Engineering College for Women, Bhimavaram, Andhra Pradesh, India

The paper describes an Interleaved Quadratic Boost DC-DC Converter. The topic of Interleaved Quadratic Boost Converters has been widely explored in the literature over the past few decades. Various studies have discussed different topologies and designs aimed at achieving high-gain conversion and high efficiency. Therefore, determining the novelty of the proposed circuit is challenging.

 In the abstract, the author mentions the converter's efficiency values, but in the body of the article, the efficiency analysis is missing. Including the converter’s efficiency formulas, derivations, and equivalent circuit under non-ideal conditions would greatly enhance the reader's understanding of the converter's dynamic performance.

 While efficiency is a critical metric for DC-DC converters, its discussion in the manuscript is minimal. Please elaborate on how the efficiency was calculated, and provide deeper insights into switching and conduction losses.

 The authors are encouraged to include a discussion on voltage and current stress on key components, which would help evaluate the reliability of the proposed converter.

 The comparison section is incomplete and requires significant improvement. It should convincingly compare the proposed converter with existing topologies. Specifically, comparisons should include power losses (both switching and conduction), power density, efficiency, and other relevant performance metrics.

 In the manuscript, hardware results are presented and explained. However, the implementation details using MATLAB software are lacking—particularly with respect to the voltage and current waveforms of inductors, capacitors, and switches.

 There is no step response analysis presented for the converter. Including this analysis would significantly improve the understanding of the converter's dynamic performance. Detailed information on how the gate drivers are integrated into the circuit and the component selection criteria would also be beneficial.

 The quality of the figures throughout the manuscript is substandard. Most figures are low-resolution and appear pixelated, which hampers clarity and readability. It is recommended to provide high-resolution vector graphics (preferably in PDF or EPS format) to ensure clear visualization of plots and circuit diagrams.

Is the study design appropriate and does the work have academic merit?

Yes

Is the work clearly and accurately presented and does it cite the current literature?

Partly

If applicable, is the statistical analysis and its interpretation appropriate?

Yes

Are all the source data underlying the results available to ensure full reproducibility?

Yes

Are the conclusions drawn adequately supported by the results?

Yes

Are sufficient details of methods and analysis provided to allow replication by others?

Partly

Reviewer Expertise:

DC - DC converters, MPPT  and multilevel inverters

I confirm that I have read this submission and believe that I have an appropriate level of expertise to confirm that it is of an acceptable scientific standard, however I have significant reservations, as outlined above.
